# Probiotic normalization of systemic inflammation in siblings of type 1 diabetes patients: an open-label pilot study

**DOI:** 10.1038/s41598-022-07203-6

**Published:** 2022-02-28

**Authors:** Susanne M. Cabrera, Alison T. Coren, Tarun Pant, Ashley E. Ciecko, Shuang Jia, Mark F. Roethle, Pippa M. Simpson, Samantha N. Atkinson, Nita H. Salzman, Yi-Guang Chen, Martin J. Hessner

**Affiliations:** 1 The Max McGee Research Center for Juvenile Diabetes, Children’s Research Institute of Children’s Wisconsin, Milwaukee, WI USA; 2 Division of Endocrinology, Department of Pediatrics, Section of Endocrinology, The Medical College of Wisconsin, 8701 Watertown Plank Road, Milwaukee, WI 53226 USA; 3 Division of Quantitative Health Sciences, Department of Pediatrics, Medical College of Wisconsin, Milwaukee, WI 53226 USA; 4 Center for Microbiome Research, Medical College of Wisconsin, Milwaukee, WI 53226 USA; 5 Department of Microbiology & Immunology, Medical College of Wisconsin, Milwaukee, WI 53226 USA; 6 Division of Gastroenterology, Department of Pediatrics, The Medical College of Wisconsin, Milwaukee, WI USA

**Keywords:** Immunology, Diseases, Medical research, Pathogenesis

## Abstract

The incidence of type 1 diabetes (T1D) has increased, coinciding with lifestyle changes that have likely altered the gut microbiota. Dysbiosis, gut barrier dysfunction, and elevated systemic inflammation consistent with microbial antigen exposure, have been associated with T1D susceptibility and progression. A 6-week, single-arm, open-label pilot trial was conducted to investigate whether daily multi-strain probiotic supplementation could reduce this familial inflammation in 25 unaffected siblings of T1D patients. Probiotic supplementation was well-tolerated as reflected by high participant adherence and no adverse events. Community alpha and beta diversity were not altered between the pre- and post-supplement stool samplings. However, LEfSe analyses identified post-supplement enrichment of the family *Lachnospiraceae*, producers of the anti-inflammatory short chain fatty acid butyrate. Systemic inflammation was measured by plasma-induced transcription and quantified with a gene ontology-based composite inflammatory index (*I.I.*_*com*_). Post-supplement *I.I.*_com_ was significantly reduced and pathway analysis predicted inhibition of numerous inflammatory mediators and activation of IL10RA. Subjects with the greatest post-supplement reduction in *I.I.*_com_ exhibited significantly lower CD4+ CD45RO+ (memory):CD4+ CD45RA+ (naïve) T-cell ratios after supplementation. Post-supplement IL-12p40, IL-13, IL-15, IL-18, CCL2, and CCL24 plasma levels were significantly reduced, while post-supplement butyrate levels trended 1.4-fold higher. Probiotic supplementation may modify T1D susceptibility and progression and warrants further study.

## Introduction

Type 1 diabetes (T1D) arises through the immune-mediated destruction of insulin-producing pancreatic β-cells and results in dependence on life-long exogenous insulin replacement therapy. Adaptive immune responses are involved in T1D pathogenesis, as T-cells comprise a significant proportion of the islet infiltrate^1^, and T-cell targeted therapies delay disease progression^2^.

While less understood, many studies have also suggested a role for altered innate immune activity in T1D pathogenesis (reviewed in Refs.^3,4^). Monocytes isolated from T1D patients spontaneously secrete IL-1β and IL-6 and induce more IL-17-secreting memory T-cells, suggesting that innate immune activity may drive adaptive responses during T1D pathogenesis by expanding the effector Th17 cell population^5^. Transcriptomic studies of unstimulated peripheral blood mononuclear cells (PBMC) drawn from individuals at high genetic risk of developing T1D have identified a type I interferon (IFN-1) signature prior to the development of islet autoantibodies^6,7^. Further, ex vivo whole blood stimulation with IFN-β or polyinosinic:polycytidylic acid, a synthetic dsRNA analog and activator of Toll-like receptor 3 (TLR3), has revealed an IFN-1 hyper-responsiveness in T1D patients compared to healthy controls^8^. Finally, independent of high-risk HLA (human leukocyte antigen) haplotypes or autoantibody status, peripheral blood mononuclear cells of T1D patients and their healthy first-degree relatives hyper-secrete cytokines (IL-1α, IFN-1, TNF-α) after stimulation with Toll-like receptor (TLR) ligands, including endotoxin and CpG oligodeoxynucleotides^9–11^. This heightened responsiveness likely contributes to an elevated baseline inflammatory state and more robust inflammatory excursions during infections that together may foster breaks in immune tolerance.

The increasing incidence of T1D in recent decades has been accompanied by a reduction in the age of onset and a lower percentage of individuals possessing high-risk HLA haplotypes^12–14^. These rapid changes cannot be explained by genetic shifts and suggest the presence of greater environmental pressure. Over this same time frame, there has been greater use of antibiotics and more widespread consumption of the low-fiber and highly processed Western diet^15–17^. Notably, the gut microbiota of modern urban humans has been found distinct from that of West African hunter-gatherers, ancient humans, and great apes^18–21^, exhibiting lower community diversity and lower abundances of potentially beneficial taxa, including short-chain fatty acid (SCFA) producers. Together, these changes are hypothesized to contribute to higher gut permeability, greater microbial antigen exposure, and increased systemic inflammation^17^. Such changes would likely have the greatest impact on those with an inherited hyper-responsiveness to innate stimuli.

Notably, gut barrier dysfunction, altered gut ultra-structure and permeability, inflammation of the duodenal mucosa, and dysbiosis are associated with human T1D^22–25^. Environmental exposures early in life may influence abnormal gut colonization and diabetes progression. While evidence is mixed, this possibility is supported by associations between neonatal probiotic usage and reduced islet autoimmunity in children at high genetic risk for T1D^26^ and associations between Cesarean birth and increased risk for T1D development^27^. Importantly, lactic acid bacteria belonging to the genera *Bifidobacterium* and *Lactobacillus* are passed from mother to child during natural birth and are important for catabolizing milk oligosaccharides^28–30^. These taxa are also known to support intestinal barrier integrity, produce anti-inflammatory SCFA, and are often used as probiotic supplements^31,32^.

Using a transcription-based bioassay, we have identified an elevated innate inflammatory state that is consistent with pattern recognition receptor (PRR) activation in T1D families and the BioBreeding (BB) rat model^33–35^. This state is associated with diabetes susceptibility yet is independent of the HLA/MHC and diabetes progression. In both species, non-progression is associated with temporal induction of IL-10/TGFβ-dependent endogenous regulatory processes that override the underlying inflammatory state. In BB rats, establishment of counter-regulation coincides with the inability of viral infections to trigger T1D^34,35^.

Modulation of the gut microbiota in BB rats can augment immunoregulatory polarization and prevent diabetes progression^36^. Modulation of the gut microbiota also influences T1D progression in non-obese diabetic (NOD) mice^37^. NOD mice with impaired microbial sensing due to MyD88 deficiency, an adapter molecule downstream of multiple TLRs, are completely protected from diabetes^38^. T1D progression in rodent models appears to be fostered in part, by an exaggerated, MyD88-dependent immune response to the commensal microbiota.

While successful prevention of T1D through probiotic supplementation has been limited to rodent models, emerging studies indicate that probiotics may favorably modulate human autoimmune disease^39–41^. Safe, broadly applicable approaches are needed to reduce the risk of triggering T1D and slowing its progression. Therefore, we conducted a pilot trial to (1) assess the feasibility of successful implementation of the study protocol, and (2) investigate the impact of daily probiotic supplement on the composition of the gut microbiota, circulating anti-inflammatory SCFA, and the familial inflammatory state associated with T1D susceptibility in unaffected siblings of diabetes patients.

## Results

### Probiotic supplementation, compliance, and validation

Twenty-five participants were enrolled to receive a 6-week course of probiotic supplementation between April 23, 2018 and November 12, 2018. Participants were not related to each other and did not have clinical T1D. Baseline characteristics are outlined in Table 1. Participants had a mean age of 12.3 years (range 6.2–17.7 years) and were 64% female. All participants had a full sibling diagnosed with T1D and 12% also had a parent with T1D. Forty percent of participants had a high-risk HLA haplotype, defined as possession of DR3 and/or DR4 (termed high-risk siblings, HRS). Sixty percent had neither DR3 nor DR4 and are termed low-risk siblings (LRS). While 76% of participants had no diabetes autoantibodies, 8% (n = 2) had a single diabetes autoantibody and 16% (n = 4) had ≥ 2 autoantibodies. Participants were generally healthy without chronic medical problems, but 8 participants had mild atopic conditions such as eczema or seasonal allergies, for which no medications were required during the study course, and 3 participants had anxiety disorder for which they took a selective serotonin reuptake inhibitor.

**Table 1:** Baseline characteristics of the 25 participants.

	N	Mean (SD) or frequency (%)
Age—years	25	12.3 (3.5)
Female sex	16	64%
White race	24	96%
Non-Hispanic ethnicity	20	80%
Sibling age at T1D diagnosis—years	25	7.4 (3.8)
**Number of first-degree relatives with T1D**		
1	22	88%
≥ 2	3	12%
BMI Z-score	25	0.43 (1.09)
**Pubertal stage**^**A**^		
Pre-pubertal	10	40%
Early/mid-pubertal	5	20%
Late/post-pubertal	10	40%
**HLA genotype**		
Non-DR3 and non-DR4	15	60%
DR3 and/or DR4	10	40%
**Number of positive autoantibodies**		
0	19	76%
1	2	8%
≥ 2	4	16%
**Positive autoantibodies**		
GAD65	6	24%
IA-2	2	8%
IAA	1	4%
ZnT8	4	16%

Pre-pubertal is defined as Tanner stage 1; mid-pubertal is defined as Tanner stage 2–3; late/post-pubertal is defined as Tanner stage 4–5.

^A^Pubertal staging was performed by a pediatric endocrinologist.

No adverse events were observed or reported; however, a third of participants reported poor palatability of the probiotic powder. Based on the number of returned probiotic sachets at the post-supplementation visit, the overall adherence was 92% (median 99%, range 52–100%).

The composition of the probiotic supplement provided to participants was analyzed through sequencing the V4 region of the 16S rDNA gene and was confirmed to consist of *Bifidobacteria*, *Lactobacillus, and Streptococcus* genera (data not shown).

### Alterations to gut microbiota

**Figure 1:**
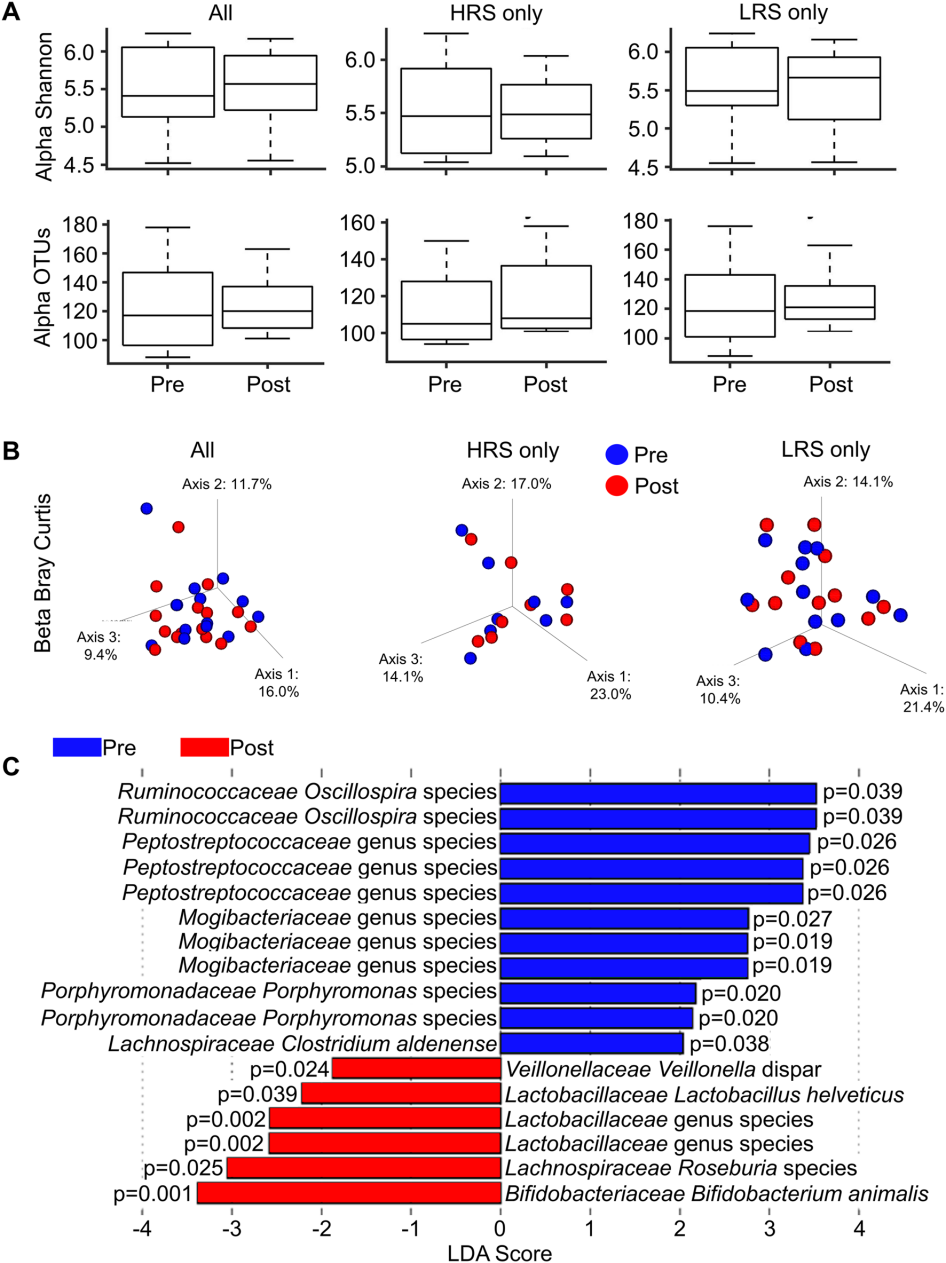
Influence of probiotic supplementation on the composition of the fecal microbiota. Total DNA was extracted from fecal samples collected from the 25 participants prior to and after probiotic supplement and the composition of the microbiota was assessed through 16 s rDNA sequencing. (**A**) Alpha diversity measures. Box- and-whisker plots comparing the Shannon index and OTU-level richness. The top and bottom of the boxes show the 75th and 25th percentile and the ends of the whiskers show the maximum and minimum values. Lines within the boxes represent median values (50th percentile). (**B**) Pre- and post-supplement beta diversity was assessed among experimental conditions using the Bray–Curtis dissimilarity index and displayed as Principal Coordinate Analysis plots. (**C**) LDA effect size of the taxa that significantly differentiate the pre- and post- supplement fecal microbiota. The LEfSe package was used to generate the LDA effect size. The following thresholds were deemed significant: LDA cut-off = |2.0|; Wilcoxon p-value = 0.05.

For the 25 participants, composition of the fecal microbiota was assessed through sequencing of the V4 region of the bacterial 16S rDNA gene, before and after the 6-week supplement period. A total of 1,030,825 quality reads were obtained from the 50 samples; among these 1,029,905 (99.91%) were mapped to at least the family level. The sequences were collapsed into operational taxonomic units (OTUs) based upon sequence identity ≥ 99%; these represented a total of 13 phyla, 53 families, and 173 unique OTUs. In terms of alpha (within sample) diversity, neither the Shannon index nor OTU richness were significantly different between the pre- and post-supplement samplings (Fig. 1A). Further, beta (between sample) diversity was not significantly different between the pre- and post-supplement communities when assessed by the Bray–Curtis dissimilarity index. Principal Coordinate Analysis showed that most of the pre-/post-probiotic sample pairs grouped with each other in a manner independent of HLA haplotype (Fig. 1B). After adjustment for a False Discovery Rate (FDR) of 5%, none of the OTUs exhibited significantly different abundances when comparing the pre- and post-supplement samplings. In addition to considering differences between HRS and LRS, we did not detect any sex or auto-antibody-dependent community differences within or between the pre- and post-supplement samplings (data not shown).

We identified differentiating features between the pre- and post-supplement communities using the Linear Discriminant Analysis (LDA)-based LEfSe approach (Fig. 1C)^42^. A total of 11 differentiating features were associated with the pre-supplement samples that possessed an absolute LDA score > 2; these included *Mogibacteriaceae*, *Porphyromonadaceae*, and *Peptostreptococcaceae* family members. A total of 5 differentiating features were associated with the post-supplement samples (LDA score > 2). Among these were *Bifidobacteriaceae* and *Lactobacillaceae* family members; these taxa were among those identified in the probiotic supplement, further supporting participant adherence. *Lachnospiraceae* (LDA score = 3.1, p = 0.025) was also identified by the post-supplement LEfSe analysis. Notably, this family (and *Veillonellaceae*, LDA score = 1.9, p = 0.024) has been reported to show decreased abundance among T1D progressors^43^.

### Probiotic supplement and systemic inflammation in siblings of T1D probands

**Figure 2:**
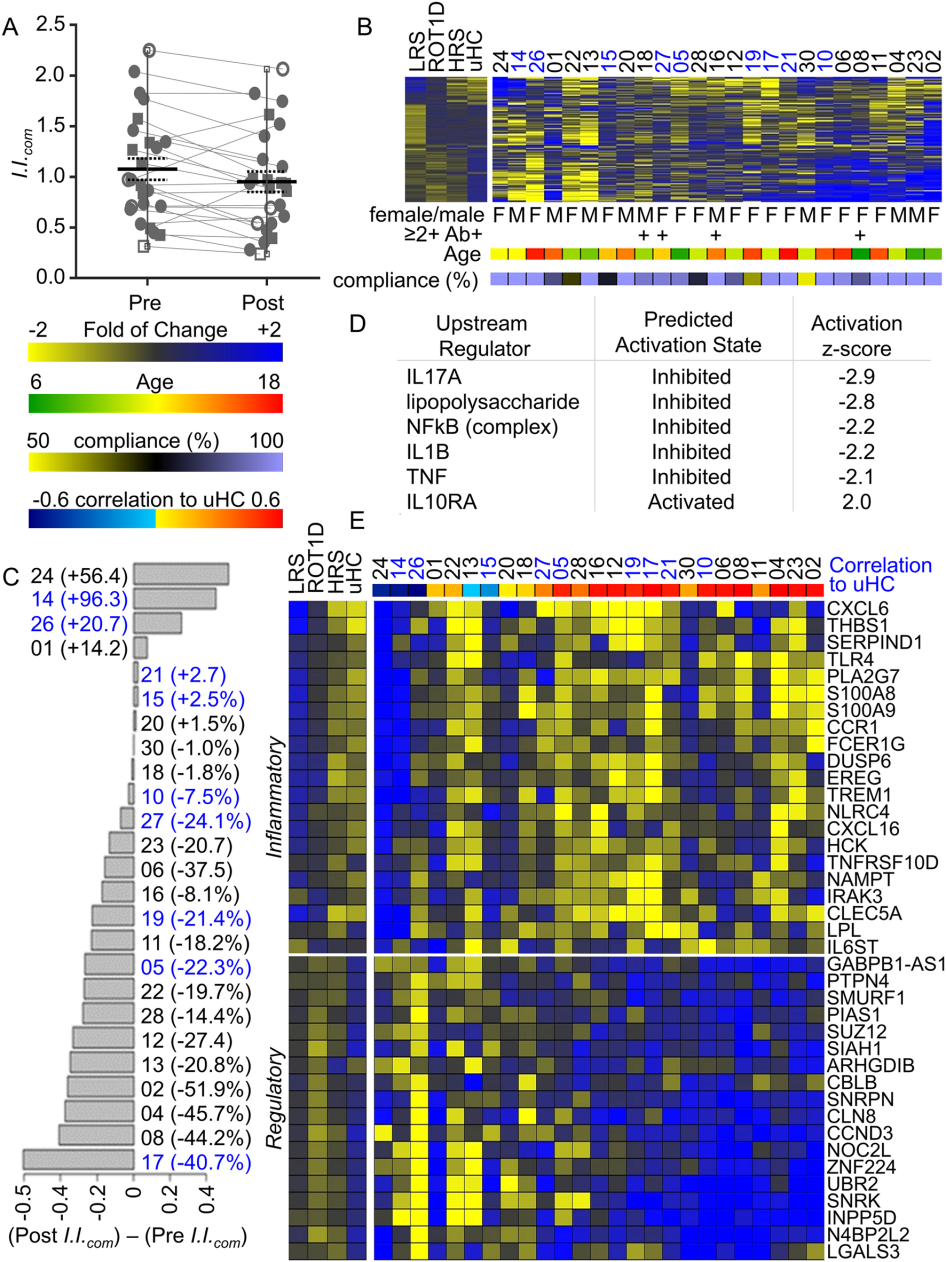
Assessment of systemic inflammation by plasma-induced transcription. Ontology-based scoring was conducted as described^33^. In Ref.^33^, inflammatory activity was associated with transcripts upregulated by LRS and RO T1D plasma and downregulated by HRS and uHC plasma; regulatory activity was associated with transcripts downregulated by LRS and RO T1D plasma and upregulated by HRS and uHC plasma. This formed the basis of *I.I.*_com_, which is determined by calculating an average ratio between the mean log intensity of the induced inflammatory genes (307) versus the mean log intensity of the induced regulatory genes (1067) of the four data subsets. (**A**) The mean *I.I.*_com_ of the 25 subjects prior to supplement was higher (1.08 ± 0.53) than that observed after supplement (0.95 ± 0.50; paired T-test, 1-tail: p = 0.017). Subjects with low-risk HLA haplotypes are represented by circles, subjects with high-risk HLA haplotypes are represented by squares. Subjects with ≥ 2 anti-islet antibodies are represented by open symbols; the significant reduction in *I.I.*_com_ remained after exclusion of these subjects (pre-supplement: 1.08 ± 0.48; post-supplement 0.97 ± 0.45; paired T-test, 1-tail: p = 0.045). *I.I.*_com_ was also reduced among the four antibody positive subjects, however the difference did not reach statistical significance (pre-supplement 1.06 ± 0.84; post-supplement 0.88 ± 0.81; paired T-test, 1-tail: p = 0.076). (**B**) Expression levels of the 1374 probe sets used to calculate *I.I.*_com_. Left panel: mean response of the LRS, ROT1D, HRS and uHC cohorts described in Ref.^33^. Right panel: supplemented siblings. Each column is a subject. Data are expressed as fold-change post- vs pre-supplement. Subject identifiers are provided, blue font indicates high-risk HLA, black font indicates low-risk HLA. Indicated are sex and autoantibody status. Color bars indicate age and percent compliance based on returned sachets. (**C**) Bar graph indicating absolute change (and percent change) in *I.I.*_com_. Subject identifiers are provided, blue indicates high risk-HLA, black indicates low-risk HLA. (**D**) Among the 1374 ttranscripts used to calculate *I.I.*_*com*_, 422 were differentially induced between the pre- and post-supplement samplings at a false discovery rate < 20% after exclusion of subjects 24, 14, 26, and 1. These were analyzed with the IPA upstream analysis tool. A z-score > 2.0 is significantly activated; a z-score >  − 2.0 is significantly inhibited. (**E**) Expression levels of well-annotated transcripts selected from the 422 transcripts showing significant differential induction. A color bar indicates Pearson’s correlation of the post-supplement 422 probe set signature to that of the uHC data set.

Plasma-induced transcription, utilizing samples drawn immediately before and after the 6-week supplement period, was used to assess how probiotics altered the familial inflammatory state that we have previously described in T1D families versus unrelated healthy controls (uHC)^33^. The subjects here were compared to our previous analysis where we identified 1,374 differentially induced probe sets among four cross-sectional cohorts: recent onset T1D (ROT1D) patients, uHC lacking family history of autoimmunity, autoantibody-negative HRS and LRS^33^. To quantify immune activity, we previously developed a gene ontology-based composite inflammatory index (*I.I.*_*com*_)^33^ based on the 1,374 transcripts in which *I.I.*_com_ is determined by calculating the ratio between the mean intensity of induced transcripts annotated as being “inflammatory” versus those annotated as being “regulatory”. High scores reflect greater inflammatory bias, while low scores reflect greater regulatory bias. On average, *I.I.*_com_ was significantly reduced after 6-weeks of probiotic supplementation (p = 0.017; Fig. 2A,B), indicating that the familial inflammatory state was lowered. No significant differences in *I.I.*_com_ based upon sex, HLA haplotype, autoantibody status, age or BMI z-score were detected among the participants pre- or post-supplement, and the degree to which *I.I.*_com_ was lowered after supplement was not dependent on these variables.

After probiotic supplement, 16/25 (64%) subjects experienced a reduction in *I.I.*_com_ of greater than 5%, while an increase of more than 5% was observed in 4/25 (16%) subjects (Fig. 2C). The four non-responders were not distinct from the remainder of the cohort by sex, age, HLA, autoantibody status, BMI z-score, or adherence to the probiotic. After exclusion of these four non-responders from the analyses, 422/1,374 (30.7%, χ^2^ p < 10E−4) of the transcripts used to calculate *I.I.*_*com*_ were differentially induced between the pre- and post-supplement samplings at a false discovery rate < 20% (Supplemental Table 1). Ingenuity Pathway Analysis (IPA) was then used to identify candidate regulators of these 422 probe sets (Fig. 2D). Consistent with a reduction in systemic inflammation, IL17A, lipopolysaccharide, NFkB, IL1B, and TNF were found significantly inhibited (Z-scores ≤ − 2.0) while IL10RA was found significantly activated (Z-score = 2.0) after probiotic supplementation. Well annotated inflammatory and immunoregulatory transcripts associated with these mediators are illustrated in Fig. 2E. In individuals with either high- or low-risk HLA haplotypes, the plasma-induced signatures showed a lower inflammatory bias post-supplement, exhibited reduced induction of inflammatory transcripts (*CXCL6*, *CXCL16*, *CCR1*, *IL6ST*, *IRAK3*, *TREM1*, *S100A8*, *S100A9*, *TNFRSF10D*) and increased induction of IL-10-dependent regulatory transcripts (*CBLB*, *PIAS1*, *PTPN4*, *INPP5D*, *SMURF1*) resulting in signatures that exhibited higher correlation to that of uHC (Fig. 2E).

### The relationship between dietary fiber intake and *I.I.*_*com*_

Participants did not receive nutritional education or instruction to alter their normal dietary patterns. No subjects reported fiber or nutritional supplement use immediately before or during the study. Twenty-four of 25 participants completed the Block 2014 Food Frequency Questionnaire (FFQ), a validated dietary self-assessment tool^44,45^. On average, participants reported daily dietary consumption of 1722 kcal (range 742–2744 kcal/day), 69 g fat (range 24–119 g/day), and 18 g of dietary fiber (range 5–37 g/day). Given the potential role of dietary fiber on the microbiota and as a prebiotic that may enhance probiotic function^46^, daily dietary fiber intake was specifically assessed in the context of guidelines from the Institutes of Medicine (IOM), which states there should be 14 g of dietary fiber per 1000 kcal/day^47^. It is known that dietary fiber intake is inadequate in most American children and < 10% achieve the IOM’s target^48^. In this study, 13% reached or exceeded the IOM target. To assess whether dietary fiber intake was associated with the degree of the probiotic-induced change in systemic inflammation, Pearson correlation coefficients were used to examine the linear relationships between variables in the 24 participants who completed the Block 2014 FFQ. For these analyses and to account for variable caloric intake across a pediatric cohort, dietary fiber was standardized as a percentage of the IOM targets achieved. A near significant relationship between dietary fiber intake and the percent change in *I.I.*_com_ was observed in that greater reported dietary fiber intake was correlated with greater reductions in *I.I.*_com_ (Pearson correlation coefficient − 0.401; p = 0.052). This suggests that dietary fiber may enhance probiotic-induced reductions in systemic inflammation. There was no association between change in *I.I.*_com_ and daily consumption of several other dietary metrics of potential interest such as the grams of soluble fiber/day or volume of daily legumes, whole grains, or yogurt consumption.

### Direct measurement of cytokines, chemokines and SCFAs

**Figure 3:**
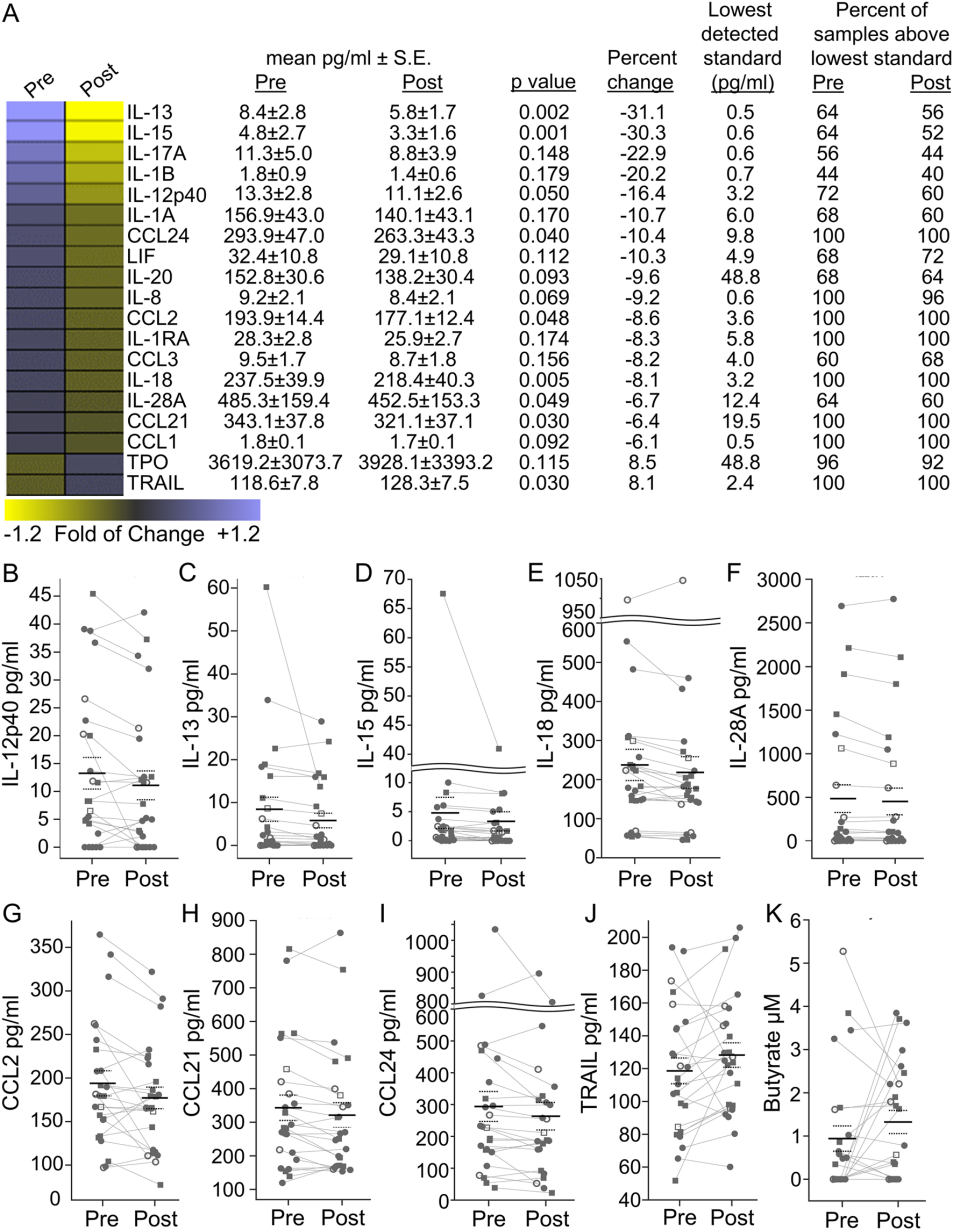
Levels of plasma borne mediators before and after probiotic supplementation. (**A–J**) Plasma samples of 25 sibling participants were assayed in duplicate before and after supplement by ELISA. In (**A**) mediators that exhibited an absolute change of more than 5% after supplement with a p-value < 0.2 are tabulated. Significant reductions in IL-12p40, IL-13, IL-15, IL-18, IL-28A, CCL2/MCP1, CCL21/C6kine, and CCL24/eotaxin 2 were observed, while TRAIL exhibited a significant increase (paired Wilcoxon rank sum test). These significantly modulated mediators are plotted in (**B–J**). Additional analytes were included in the panel (CRP, EGF, CCL11, FGF-2, Flt-3 ligand, fractalkine, G-CSF, GM-CSF, GRO, IFN-α2, IFN-γ, IL-10, IL-12p70, IL-17A, IL-1ra, IL-1α, IL-1β, IL-2, IL-3, IL-4, IL-5, IL-6, IL-7, IL-8, IL-9, IP-10, MCP-3, CCL22, MIP-1α, MIP-1β, PDGF-AA, PDGF-AB/BB, RANTES, TGFα, TNF-α, TNF-β, VEGF, sCD40L, MCP-2, BCA-1, MCP-4, I-309, IL-16, TARC, CCL26, LIF, TPO, SCF, TSLP, IL-33, IL-20, IL-21, IL-23, TRAIL, CTACK, SDF-1, ENA-78, MIP-1d, TGFB-1, TGFB-2, TGFB-3) but significant differences between the pre- and post-supplement samplings were not detected (data not shown). (**K**) Impact of supplementation on circulating butyrate levels.

We have previously measured and reported significantly elevated plasma levels of IL-12p40, IL-1α, CCL2, CCL3, and CCL4 in RO T1D patients, HRS, and LRS relative to uHC^33^. Here, we examined levels of 69 analytes in the plasma of the probiotic supplemented participants. Many proinflammatory mediators exhibited modest reductions after the supplement period (Fig. 3A); statistically significant reductions were observed for IL12p40 (− 16.4%), IL-13 (− 31.1%), IL-15 (− 30.3%), IL-18 (− 8.1%), IL-28A (− 6.7%), CCL2 (− 8.6%), CCL21/C6kine (− 6.4%), and CCL24/eotaxin 2 (− 10.4%) (Fig. 3B–I). Only TRAIL, which has both pro- and anti-inflammatory activities, showed a significant increase post-supplement (+ 8.1%) (Fig. 3J). The differences in cytokine levels were not driven by subjects that had experienced seroconversion, as the altered IL-13, IL-15, IL-18, and TRAIL levels remained significant after exclusion of the four participants possessing ≥ 2 autoantibodies.

SCFAs are the end-products of microbial fermentation of dietary fiber. Importantly, in Tregs, SCFAs activate free fatty acid receptor 2 (FFAR2) inducing differentiation and activation^49,50^. In monocytes, SCFAs activate FFAR2 and FFAR3 inducing a profound anti-inflammatory effect^51^. For these reasons, we investigated the impact of probiotic supplementation on major circulating SCFAs. While levels of acetate, propionate, valerate, and hexanoate were unchanged, on average butyrate levels trended 1.4-fold higher after probiotic supplementation (0.94 ± 1.46 μM versus 1.32 ± 1.34 μM, p = 0.11, paired t-test, 1-tailed). It has been reported that children with at least two diabetes-associated autoantibodies exhibit a dysbiotic state, characterized by a low abundance of lactate-producing and butyrate-producing species^52^. Notably, after exclusion of the four participants possessing ≥ 2 autoantibodies the increase in butyrate levels post-supplement became statistically significant (0.79 ± 1.22 μM versus 1.36 ± 1.41 μM and p = 0.045, paired t-test, 1-tailed) (Fig. 3K).

### CD4 T cell phenotypes before and after supplement

**Figure 4:**
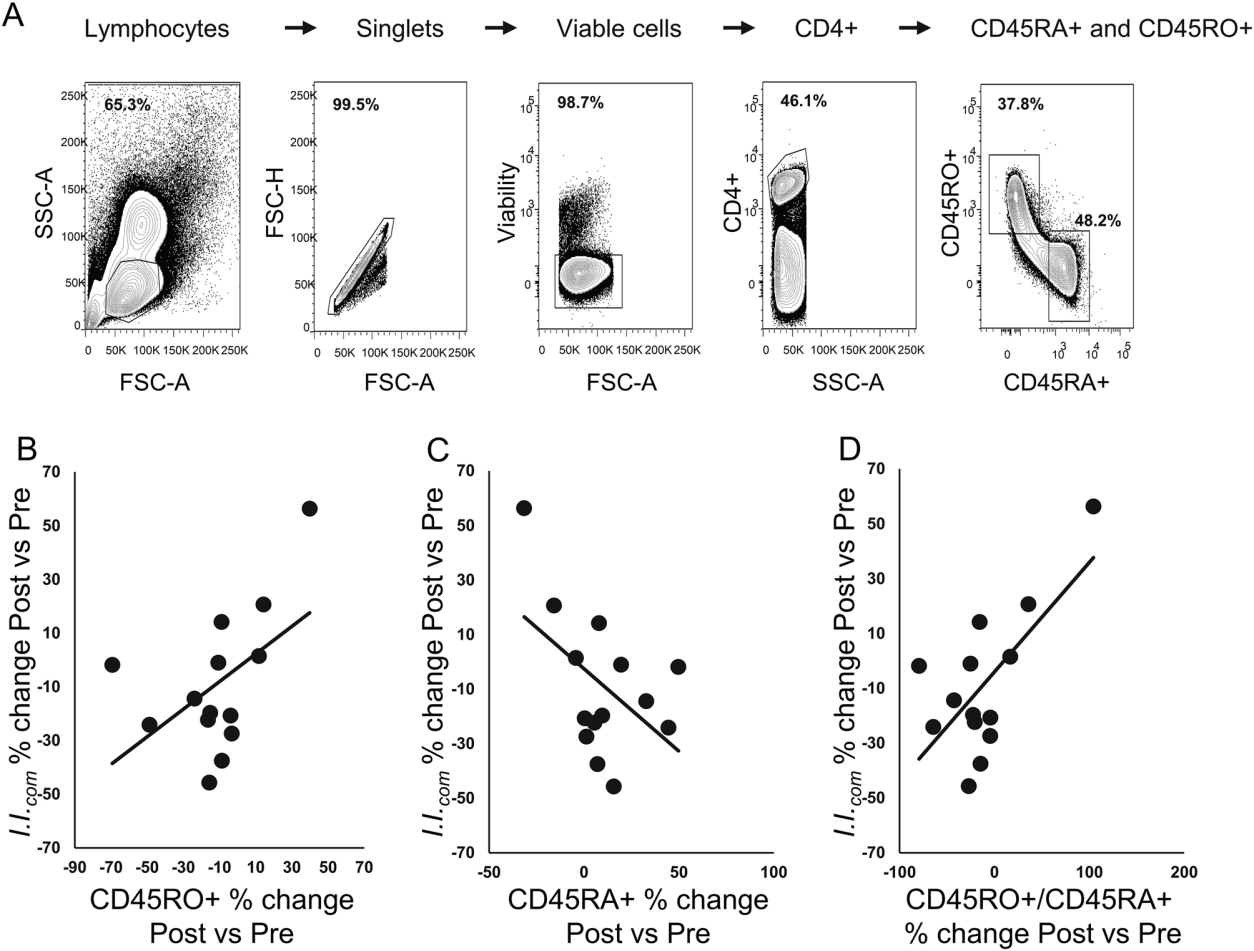
Relationship between *I.I.*_com_ and circulating naïve and memory CD4+ T-cell abundances before and after probiotic supplement. (**A**) Representative flow cytometry profiles showing the gating strategy for the naïve and memory CD4 T-cells. Naïve and memory CD4+ T-cells were respectively defined as CD45RA+/CD45RO− and CD45RA−/CD45RO+. (**B**) Relationship between percent change in *I.I.*_com_ post- versus pre-supplement plotted against the percent change in CD4+ CD45RO+ T-cells post- versus pre-supplement (Pearson’s correlation = 0.51, p = 0.061). (**C**) Relationship between percent change in *I.I.*_com_ post- versus pre-supplement plotted against the percent change in CD4+ CD45RA+ T-cells post- versus pre-supplement Pearson’s correlation =  − 0.50, p = 0.060. (**D**) Relationship between percent change in *I.I.*_com_ post- versus pre-supplement plotted against the percent change in CD4+ CD45RO+: CD4+ CD45RA+ T-cell ratio post- versus pre-supplement.

In Ref.^33^, we determined that T1D progressors do not exhibit increases in circulating activated Tregs over time. Notably, this contrasted with longitudinally studied HRS non-progressors, where we observed robust temporal increases in activated Treg, as well as a significant negative relationship between *I.I.*_com_ and the percentage of activated Treg^33^. Given these prior observations and the known effect of SCFAs on Treg differentiation and activation^49,50^, we analyzed the abundances of CD4+/CD45RA-/FOXP3^high^ activated Treg among total Treg in cryopreserved PBMC samples collected prior to and immediately after the probiotic supplement period. Significant changes in the abundances of Treg or activated Treg were not detected (data not shown) in the 14 subjects (HRS n = 3, LRS n = 11 and 2Ab + n = 2) for which samples were available. Since most participants showed a reduction in *I.I.*_com_ post-supplement, we also investigated whether those subjects experiencing the greatest reduction in *I.I.*_com_ experienced the greatest increase in activated Treg among total Treg. Again, a significant relationship was not detected. However, the analysis revealed that probiotic treatment may influence the relative abundances of naïve and memory CD4+ T-cells (Fig. 4). Notably, those subjects with the greatest reductions in *I.I.*_com_ after supplementation showed a decreasing trend in the percent CD4+ CD45RO+ memory T-cells post-supplement (Fig. 4B, Pearson’s correlation = 0.51, p = 0.061), and an increasing trend in the percent CD4+ CD45RA+ naïve T-cells post-supplement (Fig. 4C, Pearson’s correlation = 0.50, p = 0.060), and a significantly lower CD4+ CD45RO+: CD4+ CD45RA+ T-cell ratio after supplementation (Fig. 4D, Pearson’s correlation = 0.67, p = 0.008). It has been established that CD45RA expression decreases as a function of age with a concomitant increase in CD45RO+ memory T-cells^53^, possibly reflecting reductions in thymic output and the acquisition of antigen exposures over time. Notably, the CD45RO+ memory T-cell population is expanded in children with recently diagnosed T1D compared to healthy controls^54^. Here, the probiotic-associated decrease in the ratio of memory : naïve CD4+ cells is consistent with lowering of systemic inflammation.

## Discussion

Previous studies have identified an elevated inflammatory state in T1D families that is consistent with PRR activation^3,4^. In this pilot study, we provided a multi-strain probiotic to nondiabetic siblings of T1D patients with the goal of lowering this innate state. Overall, probiotic supplement was found safe and well-tolerated, as reflected by the lack of adverse events and high participant adherence rate. Immunological studies were conducted that together indicated that systemic inflammation was modestly reduced.

Plasma-induced transcription and targeted follow-up studies have successfully measured changes in immune activity associated with T1D progression. In previous longitudinal studies of T1D progressors, we observed temporal increases in *I.I.*_com_, reflecting increases in inflammatory bias^33^. Conversely, in longitudinal studies of non-progressors, especially those possessing high-risk HLA haplotypes, we observed temporal decreases in *I.I.*_com_, indicative of IL-10/TGFβ mediated regulatory polarization^33^. This overriding regulated state was associated with increases in peripheral abundances of activated regulatory T-cells (Treg, CD4^+^/CD45RA^−^/FoxP3^high^), a subpopulation possessing high suppressive capacity^33^. We have also found that newly diagnosed T1D patients are highly heterogeneous in terms of inflammatory activity^55^. Notably, those with a lower *I.I.*_com_ near onset possess higher peripheral abundances of activated Treg and experience a slower rate of C-peptide decline during the post-onset period^55^. It is known that exposure of Tregs to inflammatory inputs impairs suppressive capacity by promoting FOXP3 proteasomal degradation^56,57^, while microbiome-derived SCFAs induce Treg differentiation^49,50^. Given that (1) dysbiosis and intestinal hyper-permeability have been associated with T1D^22–25^; and (2) modulation of the gut microbiota normalizes the endogenous innate state, lowers plasma TLR4 ligand levels, and delays/prevents diabetes in BB rats^36^, we reasoned that probiotic supplementation may lower systemic inflammation in unaffected siblings of T1D patients. While significant increases in circulating activated Treg were not observed, a modest but significant reduction in *I.I.*_*com*_, our primary outcome, was observed after the six-week supplement, and reductions in *I.I.*_*com*_ significantly correlated with reductions in the CD4+ CD45RO+ (memory) : CD4+ CD45RA+ (naïve) T-helper cell ratio (p = 0.008). While not significant (p = 0.052), the greatest reductions in *I.I.*_*com*_ also correlated with the highest percentages of dietary fiber per daily caloric intake, suggesting that fiber or other prebiotics may represent a means of augmenting probiotic effect. The Block 2014 Food Frequency Questionnaire is not specifically tailored to quantify fermentable dietary fibers, and this raises the possibility of unrecognized relationships between diet and probiotic effect. Since completion of this trial, there have been efforts to develop and validate dietary indices that relate host diet to microbiome^58,59^ and these should be leveraged in future studies.

The post-supplement transcriptional signature was consistent with reduced NF-κB activation. This pathway can be triggered by cytokines, including IFN-1 and IL-1 which have been implicated in the pathogenesis of T1D and other autoimmune diseases^60,61^, as well as through PRR ligand exposure. Because plasma-induced signatures of T1D family members are consistent with PRR ligation^33^, and the plasma collected after probiotic supplement exhibited signatures consistent with reduced lipopolysaccharide exposure, we indirectly examined lipopolysaccharide levels of pre- and post-supplement plasma with reporter cells that express TLR4 and an NF-κB-inducible secreted alkaline phosphatase^36^. While TLR4 activation levels were 19.2% lower in the post- (96.1 ± 36.6 pg/ml; mean ± standard error) versus pre-supplement (119.0 ± 22.3 pg/ml) samples, this difference did not reach significance (p = 0.13) with the number of subjects studied (data not shown).

In this study we determined that probiotic supplementation did not overtly alter the gut microbiota. A limitation of this analysis was that stool samples for the second visit were collected up to a week after the last probiotic supplement. A greater change in the gut microbiota may have been observed if stool samples were collected and analyzed while the participants were still taking the supplement. Regardless, LEfSe analyses revealed enrichment of several bacterial families in the post-supplement stool samples. Among these were *Lachnospiraceae*, important anaerobic butyrate producers that are reported to induce Treg-suppression of the colonic inflammatory response^62^, as well as *Lactobacillaceae* and *Bifidobacteriaceae*, some of which directly produce butyrate, propionate, and/or acetate (e.g. *L. acidophilus* and *B. longum,* constituents of the supplement) that also promote the growth of butyrogenic taxa through bacterial cross-feeding^63^. Notably we found butyrate levels trended higher in the participants after probiotic supplement. Consistent with lowered plasma cytokine levels, butyrate supplementation has been reported to reduce responsiveness to LPS, coinciding with lowered lipopolysaccharide-induced IL-6 and IL-12 secretion in intestinal macrophages, promoting tolerance towards the intestinal microbiota^64^. In vitro studies have shown that butyrate may improve intestinal barrier function by upregulating expression of tight junction proteins^65^ and butyrate-yielding diets protect NOD mice from T1D^66^. However, de Groot et al.^67^, reported that butyrate supplement in longstanding T1D patients did not alter innate or adaptive immune cell phenotypes or alter any clinical metrics in long-standing T1D patients. It must be mentioned that these authors did not measure circulating butyrate levels, and that the effects observed in our study may be independent of changes in systemic butyrate levels.

**Figure 5:**
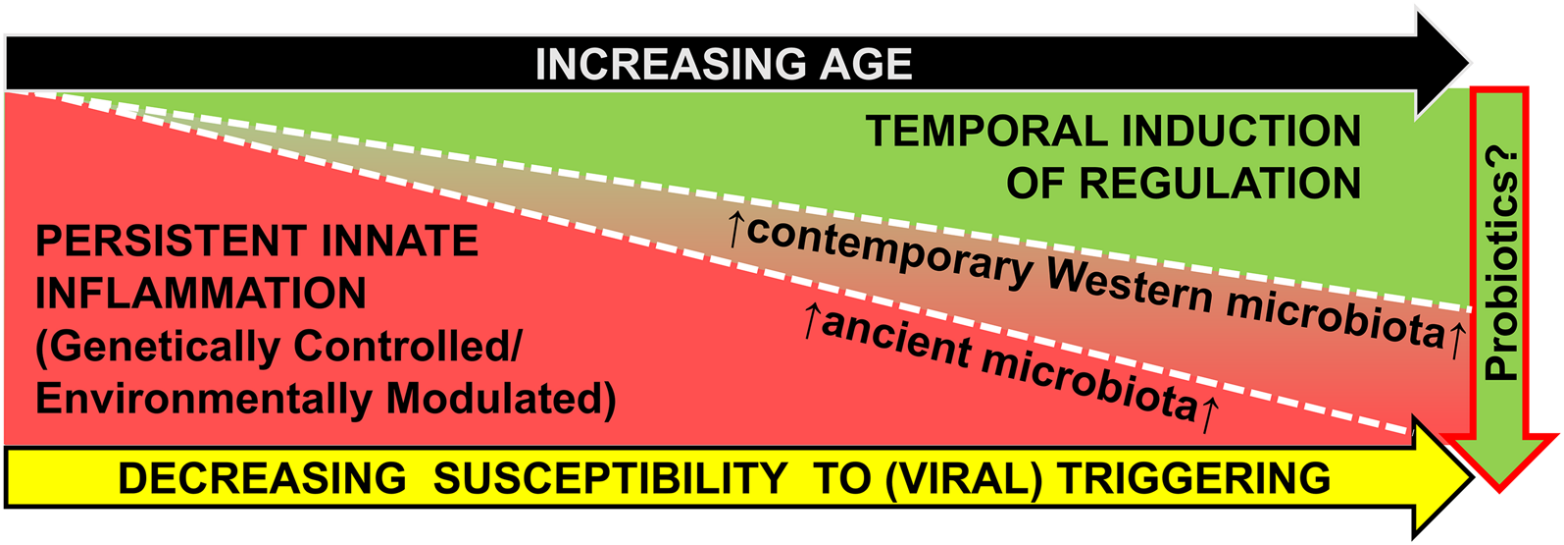
Model mechanism for how a contemporary microbiota influences the age-dependent decline in T1D susceptibility. An elevated innate inflammatory state, that includes hyper-responsiveness to TLR stimulation, is associated T1D susceptibility in human T1D families and diabetic rat models. In human T1D families, this state is independent of the HLA, presence of anti-islet antibodies, and progression of diabetes. In BioBreeding DR rats, this state is independent of insulitis, and disease progression, but is associated with the ability of Kilham’s rat virus to trigger disease progression^35^. This inflammatory state may be the consequence of genetics, diet, and intestinal microbiome. We hypothesize that this heightened inflammatory state represents a ‘‘fertile field’’ where inflammatory excursions mediated through viral infection lead to the breaking of immunologic tolerance and the progression of autoimmunity in susceptible hosts^86^. This underlying inflammatory state is subsequently supplanted by induction of an immunoregulatory state over time. As these endogenous regulatory processes become more robust, the immune balance makes environmental triggering of T1D progression less likely^33,35^. We further hypothesize modern lifestyles foster the growth of a suboptimal gut microbiota, promoting intestinal barrier leakage, increased microbial antigen exposure and systemic inflammation, while impairing induction of robust counter-regulatory mechanisms.

The results presented here suggest that the intestinal microbiota is mechanistically linked to the systemic inflammation present in T1D families. Figure 5 models how a modern low-fiber diet and associated proinflammatory microbiota may impair temporal induction of counter regulation in individuals with an inherited hyper-responsiveness to innate stimuli. Under either illustrated scenario, genetically at-risk younger children have a higher level of baseline inflammation and are more susceptible to tolerance breaking inflammatory excursions, consistent with (1) the age-related decline in the risk of multiple antibody seroconversion during childhood^68^, and (2) the commonly observed pediatric onset of T1D. In the absence of a high fiber diet and protective microbiota, evidence suggests that the intestinal mucus barrier can be compromised^69^, promoting bacterial translocation and systemic inflammation, while impairing Treg differentiation and proliferation. Consequently, temporal induction of counter regulation is slower and less robust, fostering a higher incidence of T1D and younger age of onset.

This pilot study suggests that it feasible to modify the inflammatory state associated with T1D susceptibility through probiotic supplement. It provides a needed framework for future studies that encompasses a basis for the estimation of appropriate sample sizes. This pilot study also has several limitations. These include its modest sample size, lack of a placebo group, analysis of only two timepoints, a heterogeneous study population in terms of HLA and autoantibody status, and modest measured effects. Further, the probiotic was not assessed for viability and analysis of the microbiota was limited to amplicon sequencing. Finally, the absence of a non-T1D family comparator group did not allow us to determine if these measured effects would be observed to the same degree in a non-susceptible healthy control population. There is a substantial need for safe, broadly applicable therapies to reduce the risk in susceptible individuals and slow the progression of T1D both before and after clinical onset of disease. This study indicates that additional investigations of prebiotic and probiotic strategies are warranted as they may be efficacious either alone or in combination with other therapeutic agents. Future studies are aimed at associating probiotic-induced immunologic changes with measures of β-cell function, as this may suggest a prevention strategy for those with underlying T1D susceptibility.

## Materials and methods

### Study participants

Twenty-five children were recruited through the Children’s Wisconsin (CW) Diabetes Clinic between April 2018 and November 2018. Inclusion criteria for participants were healthy children aged 5–17 years who had a full sibling diagnosed with T1D and were treatment naïve of any immunomodulatory, antibiotic, or probiotic agents within the past 3 months. Only one sibling per family could participate. Exclusion criteria were the presence of celiac disease, other chronic inflammatory/autoimmune disease besides hypothyroidism well-controlled with levothyroxine, or the use of medications known to affect gastrointestinal function or glucose metabolism (such as antibiotics) within the past month.

### Ethical statement

All participants and their guardian(s) provided written informed consent and those aged 7–14 years of age also provided informed assent. Study procedures were approved by the Children’s Wisconsin Institutional Research Board (IRB #1171017-6) and were consistent with the Declaration of Helsinki. The study was prospectively registered with the National Institutes of Health (clinicaltrials.gov NCT03423589; 06/02/2018).

### Study procedures

During this single-arm, open label trial, all participants received a six-week course of a multi-strain probiotic supplement containing *Bifidobacteria longum*, *B. infantis*, *B. breve*, *Lactobacillus acidophilus*, *L. casei*, *L. delbrueckii subspecies Bulgaricus*, *L. plantarum*, and *Streptococcus salivarius subspecies thermophilus* (Alfasigma USA, Inc. Covington, LA, USA).

Similar formulations have been found safe in pediatric patients^70–73^ and able to delay/prevent T1D progression in the NOD mouse^37^. The probiotic was purchased from a wholesale pharmacy distributor and the supplement manufacturer was not involved in any aspect of the study concept, design, or execution. Dosing, which was based upon the weight-dependent scheme described by Miele et al.^71^, consisted of 450 billion colony forming units (CFU) by mouth daily for those aged < 11 years and 900 billion CFU by mouth day for those aged ≥ 11 years. Participants were asked to store the probiotic sachets in the refrigerator until use and were instructed to sprinkle the contents on cold or room temperature food or suspend into non-carbonated drinks. Before starting the probiotic, participants provided a medical history and had a physical examination performed by a pediatric endocrinologist, including Tanner pubertal staging. Participants also completed the Block 2014 Food Frequency Questionnaire (NutritionQuest, Berkeley, CA, USA), a validated instrument of dietary intake^44,74,75^ over the previous 12 months, with the assistance of their guardian(s).

Peripheral blood was collected within 24 h before starting the probiotic and within 1 week after completing the six-week probiotic supplementation. Blood was drawn into K + EDTA or acid citrate dextrose solution A anti-coagulant and components were immediately separated by Ficoll-Histopaque (Sigma Aldrich, St. Louis, MO, USA) density gradient centrifugation. PBMC were cryopreserved and plasma was stored at − 80 °C until use. Measurement of autoantibodies towards glutamic acid decarboxylase (GAD), insulinoma antigen 2 (IA-2), insulin (INS) and zinc transporter 8 (ZnT8) was conducted as described^76^. HLA genotyping was conducted as described^77^. Subjects returned unused sachets at study end and the adherence rate was calculated by Children’s Wisconsin Investigational Drug Services.

### Analysis of the fecal microbiota

Using a kit supplied to participant families, fresh stool was collected at home and immediately preserved in RNAlater (ThermoFisher, Waltham, MA, USA). Participants were instructed to perform this procedure no more than 3 days before the study visit and keep the stool sample in their home refrigerator until transport to the research center. Upon arrival to the laboratory, stool samples were stored at − 80 °C. Stool was collected ≤ 72 h before starting the probiotic and within 1 week after completing the 6-week probiotic supplementation. DNA was extracted from stool using the MoBIO PowerSoil Isolation Kit (Qiagen, Germantown, MD, USA). The V4 region of the 16S rDNA gene was amplified by PCR and sequenced (Diversigen, Baylor College of Medicine, Waco, TX, USA) on the MiSeq platform (Illumina, San Diego, CA, USA) using the 2 × 250-bp protocol, yielding pair-end reads^78^. QIIME2 (v. 2019.7) was used to analyze the paired-end 16S rDNA sequencing reads^79^. Sequences were imported and summarized to check quality and chimeric sequences removed with DADA2^80^. The representative sequences were aligned^81^, masked for hypervariable regions, and then phylogenetic trees were produced. A classifier was generated to assign taxonomy to the reads using the 99% similarity files of the GreenGenes database v. 13_8 and the 515–806 region (V4) of the 16S gene^82^. Taxonomy was assigned to the feature table to generate relative abundance tables and make taxonomy bar plots. Alpha and beta diversity metrics were analyzed using QIIME2. LEfSe, Linear Discriminant Analysis (LDA) effect size, was run to determine enriched organisms in the pre- and post-supplement samples^42^. Sequencing data files have been deposited at The National Center for Biotechnology Information Sequence Read Archive (Accession Number: PRJNA714090). The probiotic supplement provided to participants was analyzed by sequencing the V4 region of the16S rDNA region, metagenomic sequencing to assess its composition was not conducted. These analyzed sachets had the same lot number and were purchased at the same time as those dispensed to the participants.

### Transcriptional analyses

Plasma-induced transcription assays, the primary outcome measure, utilized cryopreserved “responder” PBMC of a single healthy blood donor (Cellular Technology Ltd., Shaker Heights, OH, USA) cultured with 200 μl participant plasma in 300 μl RPMI 1640 medium. After a 9-h culture, RNA was extracted and induced transcription was measured using Affymetrix GeneChip Human Genome U133 plus 2.0 arrays (Affymetrix, Santa Clara, CA, USA) as described^33^. Array data was subjected to global median normalization with Bioconductor Robust Multi-array Analysis^83^.

### Statistics

The significance of gene expression measurements was determined by ANOVA and the rate of type I errors in multiple testing was assessed by false discovery rate (FDR) determined with Partek Genomics Suite 6.6 (Partek, Saint Louis, MO, USA). Ontological analysis utilized IPA (QIAGEN, Redwood City, CA, USA). Hierarchical clustering was conducted with Genesis^84^. All gene expression data files have been deposited at The National Center for Biotechnology Information Gene Expression Omnibus (Accession Number: GSE162622).

### Analysis of plasma mediators

Plasma samples were analyzed in duplicate using the Human Cytokine/Human Chemokine Array 65-plex Panel (HD65; Eve Technologies, Calgary, AB. Canada). Plasma C reactive protein (CRP) levels were measured by ELISA (ABCAM, Cambridge, MA, USA).

### Measurement of circulating short-chain fatty acids

Plasma SCFA levels were measured before and after supplementation by the Mayo Clinic Metabolomics Research Core using a targeted mass spectrometry approach that utilized 13C or 15N isotope labeled reference compounds^85^.

### Analysis of circulating T-cell subsets

PBMCs were stained with the fixable Live/Dead Violet dye (Life Technologies, Grand Island, NY) for 30 min on ice, followed by surface staining for anti-CD4 (clone RPA-T4), anti-CD25 (clone M-A251), anti-CD45RO (clone UCHL1), anti-CD45RA (clone HI100), and anti-CD127 (clone HIR-7R-M21) (BD Bioscience, San Jose, CA) on ice for 30 min, followed by intracellular staining with anti-FOXP3 (clone PCH101) (eBioscience, San Diego, CA). Stained cells were analyzed on a LSR II flow cytometer (BD Bioscience). Data were analyzed using FlowJo software 9.0 (TreeStar, Ashland, OR, USA).

## Supplementary Information

Supplementary Table 1.
