# Supplementary material for: Probiotic normalization of systemic inflammation in siblings of type 1 diabetes patients: an open-label pilot study
Source: Sci Rep. 2022 Feb 28;12:3306. doi: 10.1038/s41598-022-07203-6 (PMC8885673; doi:10.1038/s41598-022-07203-6)
Supplement: Supplementary file 1 — Supplementary Table 1. [file 41598_2022_7203_MOESM1_ESM.pdf]

Probeset ID	Gene Symbol	Gene Title	Mean log2 intensity for 21 POST	Mean log2 intensity for 21 PRE	422 FDR <0.2 1=YES 0=NO	Log2 Ratio 21POST vs21PRE	Fold-Change 21POST vs21PRE	p value	FDR
217546_at	MT1M	metallothionein 1M	4.82	5.36	1	-0.53	-1.45	0.00	0.060
203980_at	FABP4	fatty acid binding protein 4, adipocyte	7.54	7.87	1	-0.33	-1.26	0.11	0.199
219607_s_at	MS4A4A	membrane-spanning 4-domains, subfamily A, member 4A	5.06	5.26	1	-0.20	-1.15	0.10	0.189
206336_at	CXCL6	chemokine (C-X-C motif) ligand 6	5.56	5.84	1	-0.27	-1.21	0.10	0.187
235086_at	THBS1	thrombospondin 1	8.18	8.56	1	-0.38	-1.30	0.02	0.094
1558404_at	LINC00622	long intergenic non-protein coding RNA 622	2.83	3.21	1	-0.37	-1.29	0.01	0.085
239519_at	NRP1	---	4.42	4.68	1	-0.27	-1.20	0.11	0.198
234932_s_at	CDCP1	CUB domain containing protein 1	3.89	4.22	1	-0.33	-1.26	0.02	0.092
201109_s_at	THBS1	thrombospondin 1	9.38	9.66	1	-0.28	-1.21	0.02	0.092
201108_s_at	THBS1	thrombospondin 1	8.57	8.83	1	-0.26	-1.20	0.03	0.121
239336_at	THBS1	thrombospondin 1(THBS1)	4.81	5.12	1	-0.31	-1.24	0.04	0.125
205576_at	SERPIND1	serpin peptidase inhibitor, clade D (heparin cofactor), member 1	3.08	3.25	1	-0.17	-1.12	0.09	0.179
1569203_at	CXCL2	chemokine (C-X-C motif) ligand 2	6.07	6.30	1	-0.23	-1.17	0.08	0.170
231897_at	PTGR1	prostaglandin reductase 1	4.84	5.11	1	-0.27	-1.21	0.01	0.086
217678_at	SLC7A11	solute carrier family 7 (anionic amino acid transporter light chain, xc- system), member 11	9.29	9.51	1	-0.22	-1.16	0.03	0.117
1565162_s_at	MGST1	microsomal glutathione S-transferase 1	6.96	7.13	1	-0.17	-1.13	0.09	0.179
204058_at	ME1	malic enzyme 1, NADP(+)-dependent, cytosolic	3.19	3.50	1	-0.31	-1.24	0.01	0.085
211571_s_at	VCAN	versican	4.09	4.33	1	-0.24	-1.18	0.02	0.094
226281_at	DNER	delta/notch like EGF repeat containing	2.98	3.18	1	-0.19	-1.14	0.04	0.125
220416_at	ATP8B4	ATPase, class I, type 8B, member 4	5.47	5.78	1	-0.31	-1.24	0.00	0.064
210152_at	LILRB4	leukocyte immunoglobulin-like receptor, subfamily B (with TM and ITIM domains), member 4	4.21	4.37	1	-0.15	-1.11	0.08	0.170
204864_s_at	IL6ST	interleukin 6 signal transducer	5.24	5.42	1	-0.18	-1.13	0.04	0.121
244774_at	PHACTR2	phosphatase and actin regulator 2	5.32	5.52	1	-0.20	-1.15	0.11	0.197
204112_s_at	HNMT	histamine N-methyltransferase	5.98	6.20	1	-0.22	-1.16	0.09	0.181
202877_s_at	CD93	CD93 molecule	7.11	7.31	1	-0.19	-1.14	0.08	0.171
212158_at	SDC2	syndecan 2	7.41	7.59	1	-0.18	-1.13	0.11	0.199
212154_at	SDC2	syndecan 2	7.35	7.57	1	-0.22	-1.17	0.02	0.095
215646_s_at	VCAN	versican	4.13	4.39	1	-0.26	-1.20	0.03	0.113
204619_s_at	VCAN	versican	4.08	4.35	1	-0.27	-1.20	0.05	0.137
204620_s_at	VCAN	versican	6.17	6.43	1	-0.27	-1.20	0.03	0.115
212157_at	SDC2	syndecan 2	4.81	5.01	1	-0.20	-1.15	0.02	0.100
206026_s_at	TNFAIP6	tumor necrosis factor, alpha-induced protein 6	8.75	8.91	1	-0.16	-1.12	0.05	0.141
204971_at	CSTA	cystatin A (stefin A)	8.52	8.67	1	-0.16	-1.11	0.05	0.138
235740_at	MCTP1	multiple C2 domains, transmembrane 1	5.13	5.30	1	-0.17	-1.13	0.10	0.191
206214_at	PLA2G7	phospholipase A2, group VII (platelet-activating factor acetylhydrolase, plasma)	7.42	7.67	1	-0.24	-1.18	0.01	0.086
202917_s_at	S100A8	S100 calcium binding protein A8	8.49	8.81	1	-0.31	-1.24	0.01	0.077
203535_at	S100A9	S100 calcium binding protein A9	8.05	8.34	1	-0.29	-1.22	0.00	0.056
212298_at	NRP1	neuropilin 1	5.51	5.81	1	-0.31	-1.24	0.02	0.093
226066_at	MITF	microphthalmia-associated transcription factor	5.92	6.16	1	-0.24	-1.18	0.01	0.086
219890_at	CLEC5A	C-type lectin domain family 5, member A	6.66	6.95	1	-0.29	-1.22	0.02	0.098
230748_at	SLC16A6	solute carrier family 16, member 6	6.76	6.97	1	-0.21	-1.16	0.03	0.114
202435_s_at	CYP1B1	cytochrome P450, family 1, subfamily B, polypeptide 1	9.36	9.65	1	-0.29	-1.23	0.00	0.064
202436_s_at	CYP1B1	cytochrome P450, family 1, subfamily B, polypeptide 1	9.97	10.24	1	-0.26	-1.20	0.02	0.092
202437_s_at	CYP1B1	cytochrome P450, family 1, subfamily B, polypeptide 1	9.11	9.38	1	-0.26	-1.20	0.01	0.092
219434_at	TREM1	triggering receptor expressed on myeloid cells 1	6.31	6.52	1	-0.20	-1.15	0.01	0.088
205767_at	EREG	epiregulin	10.27	10.42	1	-0.15	-1.11	0.07	0.164
212681_at	EPB41L3	erythrocyte membrane protein band 4.1-like 3	6.79	6.92	1	-0.13	-1.09	0.11	0.199
206710_s_at	EPB41L3	erythrocyte membrane protein band 4.1-like 3	7.70	7.94	1	-0.25	-1.19	0.01	0.083
213338_at	TMEM158	transmembrane protein 158 (gene/pseudogene)	6.97	7.16	1	-0.19	-1.14	0.06	0.147
227080_at	ZNF697	zinc finger protein 697	7.20	7.42	1	-0.22	-1.17	0.01	0.088
203548_s_at	LPL	lipoprotein lipase	6.62	6.79	1	-0.17	-1.13	0.07	0.169
221060_s_at	TLR4	toll-like receptor 4	5.23	5.44	1	-0.21	-1.16	0.04	0.122
214560_at	FPR3	formyl peptide receptor 3	4.33	4.54	1	-0.22	-1.16	0.05	0.137
230422_at	FPR3	formyl peptide receptor 3	4.59	4.89	1	-0.30	-1.23	0.01	0.085
205237_at	FCN1	ficolin (collagen/fibrinogen domain containing) 1	6.08	6.25	1	-0.17	-1.12	0.03	0.121
204059_s_at	ME1	malic enzyme 1, NADP(+)-dependent, cytosolic	5.68	5.94	1	-0.25	-1.19	0.04	0.127
221731_x_at	VCAN	versican	6.05	6.26	1	-0.21	-1.15	0.06	0.144
218559_s_at	MAFB	v-maf avian musculoaponeurotic fibrosarcoma oncogene homolog B	9.46	9.67	1	-0.21	-1.16	0.02	0.094
220088_at	C5AR1	complement component 5a receptor 1	6.67	6.82	1	-0.15	-1.11	0.08	0.173
201506_at	TGFBI	transforming growth factor, beta-induced, 68kDa	5.97	6.14	1	-0.17	-1.12	0.07	0.169
1552553_a_at	NLRC4	NLR family, CARD domain containing 4	5.10	5.26	1	-0.16	-1.12	0.08	0.170
211067_s_at	GAS7	growth arrest-specific 7	4.97	5.14	1	-0.17	-1.12	0.03	0.113
223454_at	CXCL16	chemokine (C-X-C motif) ligand 16	7.94	8.08	1	-0.14	-1.10	0.04	0.123
222876_s_at	ADAP2	ArfGAP with dual PH domains 2	4.90	5.06	1	-0.16	-1.12	0.04	0.125
208018_s_at	HCK	HCK proto-oncogene, Src family tyrosine kinase	8.55	8.68	1	-0.13	-1.10	0.03	0.113
214211_at	FTH1	ferritin, heavy polypeptide 1	9.72	9.88	1	-0.16	-1.12	0.00	0.056
210146_x_at	LILRB2	leukocyte immunoglobulin-like receptor, subfamily B (with TM and ITIM domains), member 2	6.34	6.49	1	-0.14	-1.10	0.05	0.132
206881_s_at	LILRA3	leukocyte immunoglobulin-like receptor, subfamily A (without TM domain), member 3	5.82	5.97	1	-0.15	-1.11	0.02	0.108
210422_x_at	SLC11A1	solute carrier family 11 (proton-coupled divalent metal ion transporter), member 1	5.11	5.26	1	-0.15	-1.11	0.01	0.086
205098_at	CCR1	chemokine (C-C motif) receptor 1	8.65	8.82	1	-0.17	-1.13	0.02	0.098
210784_x_at	102725029 //	leukocyte immunoglobulin-like receptor, subfamily A (with TM domain), member 6 /// leukocyte immunoglobulin-lik	6.83	6.93	1	-0.10	-1.07	0.04	0.131
211336_x_at	LILRB1	leukocyte immunoglobulin-like receptor, subfamily B (with TM and ITIM domains), member 1	6.48	6.57	1	-0.10	-1.07	0.07	0.168
222218_s_at	PILRA	paired immunoglobin-like type 2 receptor alpha	6.84	6.96	1	-0.11	-1.08	0.03	0.114
204122_at	TYROBP	TYRO protein tyrosine kinase binding protein	10.03	10.18	1	-0.15	-1.11	0.00	0.056
205126_at	VRK2	vaccinia related kinase 2	8.20	8.34	1	-0.14	-1.10	0.00	0.056
204232_at	FCER1G	Fc fragment of IgE, high affinity I, receptor for; gamma polypeptide	8.66	8.79	1	-0.13	-1.10	0.03	0.117
1554899_s_at	FCER1G	Fc fragment of IgE, high affinity I, receptor for; gamma polypeptide	8.50	8.59	1	-0.09	-1.07	0.11	0.199
228937_at	LACC1	laccase (multicopper oxidoreductase) domain containing 1	7.25	7.41	1	-0.16	-1.12	0.05	0.137
212820_at	DMXL2	Dmx-like 2	8.65	8.80	1	-0.15	-1.11	0.02	0.094
202381_at	ADAM9	ADAM metallopeptidase domain 9	8.06	8.23	1	-0.17	-1.13	0.02	0.093
225283_at	ARRDC4	arrestin domain containing 4	6.19	6.38	1	-0.19	-1.14	0.01	0.085
201785_at	RNASE1	ribonuclease, RNase A family, 1 (pancreatic)	4.54	4.72	1	-0.18	-1.14	0.01	0.085
223344_s_at	MS4A7	membrane-spanning 4-domains, subfamily A, member 7	9.26	9.39	1	-0.13	-1.09	0.04	0.123
223343_at	MS4A7	membrane-spanning 4-domains, subfamily A, member 7	8.63	8.76	1	-0.13	-1.09	0.03	0.117
224358_s_at	MS4A7	membrane-spanning 4-domains, subfamily A, member 7	5.98	6.14	1	-0.16	-1.12	0.05	0.138
211135_x_at	102725029 //	leukocyte immunoglobulin-like receptor, subfamily A (with TM domain), member 6 /// leukocyte immunoglobulin-lik	6.45	6.62	1	-0.17	-1.12	0.01	0.085
210225_x_at	102725029 //	leukocyte immunoglobulin-like receptor, subfamily A (with TM domain), member 6 /// leukocyte immunoglobulin-lik	6.91	7.07	1	-0.16	-1.12	0.01	0.085
228181_at	SLC30A1	solute carrier family 30 (zinc transporter), member 1	6.51	6.71	1	-0.20	-1.14	0.00	0.056
229900_at	CD109	CD109 molecule	6.18	6.34	1	-0.16	-1.12	0.01	0.092
204151_x_at	AKR1C1	aldo-keto reductase family 1, member C1	6.79	6.95	1	-0.16	-1.12	0.05	0.132
224480_s_at	GPAT3	glycerol-3-phosphate acyltransferase 3	8.23	8.38	1	-0.15	-1.11	0.08	0.171
224829_at	CPEB4	cytoplasmic polyadenylation element binding protein 4	7.32	7.46	1	-0.14	-1.10	0.01	0.085
224414_s_at	CARD6	caspase recruitment domain family, member 6	6.82	6.97	1	-0.15	-1.11	0.01	0.088
201266_at	TXNRD1	thioredoxin reductase 1	9.93	10.07	1	-0.14	-1.10	0.00	0.072
207528_s_at	SLC7A11	solute carrier family 7 (anionic amino acid transporter light chain, xc- system), member 11	7.61	7.79	1	-0.18	-1.13	0.01	0.077
202912_at	ADM	adrenomedullin	7.47	7.66	1	-0.18	-1.14	0.00	0.056
226545_at	CD109	CD109 molecule	6.27	6.37	1	-0.10	-1.07	0.06	0.148
227345_at	TNFRSF10D	tumor necrosis factor receptor superfamily, member 10d, decoy with truncated death domain	5.79	5.93	1	-0.14	-1.10	0.08	0.170
217853_at	TNS3	tensin 3	6.98	7.17	1	-0.19	-1.14	0.03	0.113
209921_at	SLC7A11	solute carrier family 7 (anionic amino acid transporter light chain, xc- system), member 11	9.83	10.02	1	-0.20	-1.15	0.01	0.077
225188_at	RAPH1	Ras association (RalGDS/AF-6) and pleckstrin homology domains 1	6.06	6.23	1	-0.16	-1.12	0.04	0.125
1568830_at	IRAK3	interleukin 1 receptor associated kinase 3	4.84	4.99	1	-0.15	-1.11	0.09	0.175
224595_at	SLC44A1	solute carrier family 44 (choline transporter), member 1	7.73	7.88	1	-0.15	-1.11	0.01	0.088
228573_at	ANTXR2	anthrax toxin receptor 2	6.97	7.11	1	-0.14	-1.10	0.01	0.077
218854_at	DSE	dermatan sulfate epimerase	9.11	9.20	1	-0.09	-1.07	0.08	0.170
243296_at	NAMPT	nicotinamide phosphoribosyltransferase	9.26	9.43	1	-0.17	-1.13	0.07	0.157
222692_s_at	FNDC3B	fibronectin type III domain containing 3B	7.30	7.43	1	-0.13	-1.09	0.11	0.198
222877_at	NRP2	neuropilin 2	5.83	6.01	1	-0.18	-1.13	0.05	0.138
209135_at	ASPH	aspartate beta-hydroxylase	7.05	7.18	1	-0.12	-1.09	0.11	0.198
208892_s_at	DUSP6	dual specificity phosphatase 6	8.76	8.92	1	-0.17	-1.12	0.04	0.125
208891_at	DUSP6	dual specificity phosphatase 6	9.45	9.59	1	-0.14	-1.10	0.07	0.157
236495_at	---	---	4.48	4.72	1	-0.24	-1.18	0.05	0.132
226364_at	HIP1	huntingtin interacting protein 1	4.31	4.50	1	-0.20	-1.15	0.05	0.132
212944_at	SLC5A3	solute carrier family 5 (sodium/myo-inositol cotransporter), member 3	10.05	10.20	1	-0.15	-1.11	0.07	0.164
241315_at	---	---	3.22	3.36	1	-0.14	-1.10	0.03	0.117
241894_at	VMO1	vitelline membrane outer layer 1 homolog (chicken)	4.38	4.53	1	-0.14	-1.11	0.03	0.117
235751_s_at	VMO1	vitelline membrane outer layer 1 homolog (chicken)	6.15	6.28	1	-0.13	-1.09	0.10	0.187
209057_x_at	CDC5L	cell division cycle 5-like	5.46	5.64	1	-0.17	-1.13	0.03	0.121
226036_x_at	CASP2	caspase 2	3.43	3.54	1	-0.11	-1.08	0.11	0.199
235645_at	ESCO1	establishment of sister chromatid cohesion N-acetyltransferase 1	7.11	7.25	1	-0.14	-1.10	0.08	0.175
240544_at	---	---	2.71	2.81	1	-0.09	-1.07	0.11	0.199
223999_at	PPIL2	peptidylprolyl isomerase (cyclophilin)-like 2	2.61	2.74	1	-0.12	-1.09	0.02	0.092
230265_at	---	---	5.55	5.63	1	-0.09	-1.06	0.11	0.199
242429_at	ZNF567	zinc finger protein 567	5.90	6.01	1	-0.12	-1.09	0.09	0.181
211000_s_at	IL6ST	interleukin 6 signal transducer	7.06	7.26	1	-0.20	-1.15	0.01	0.085
240105_at	---	---	6.80	6.94	1	-0.14	-1.10	0.09	0.179
222576_s_at	EIF2C1	argonaute RISC catalytic component 1	5.27	5.41	1	-0.14	-1.10	0.09	0.178
217856_at	RBM8A	RNA binding motif protein 8A	7.39	7.54	1	-0.15	-1.11	0.04	0.125
219653_at	LSM14B	LSM family member 14B	3.83	3.98	1	-0.15	-1.11	0.02	0.100
220946_s_at	SETD2	SET domain containing 2	6.20	6.35	1	-0.15	-1.11	0.05	0.132
211993_at	WNK1	WNK lysine deficient protein kinase 1	7.36	7.50	1	-0.14	-1.10	0.03	0.113
240626_at	---	---	2.38	2.48	1	-0.10	-1.07	0.05	0.140
235009_at	BOD1L1	biorientation of chromosomes in cell division 1-like 1	7.80	7.89	1	-0.09	-1.06	0.09	0.179
227152_at	KIAA1551	KIAA1551	10.82	10.90	1	-0.09	-1.06	0.02	0.095
205787_x_at	41155 /// ZC3	zinc finger CCCH-type domain-containing-like /// zinc finger CCCH-type containing 11A	6.92	7.06	1	-0.14	-1.10	0.03	0.116
221765_at	UGCG	UDP-glucose ceramide glucosyltransferase	7.46	7.58	1	-0.12	-1.09	0.03	0.115
212926_at	SMC5	structural maintenance of chromosomes 5	6.92	7.08	1	-0.16	-1.12	0.02	0.092
211242_x_at	KIR2DL4	killer cell immunoglobulin-like receptor, two domains, long cytoplasmic tail, 4	3.46	3.55	1	-0.09	-1.07	0.04	0.124
224231_at	PRO0471	uncharacterized LOC28994	2.13	2.22	1	-0.09	-1.07	0.03	0.118
223263_s_at	FGFR1OP2	FGFR1 oncogene partner 2	7.56	7.65	1	-0.09	-1.06	0.05	0.140
219159_s_at	SLAMF7	SLAM family member 7	10.05	10.15	1	-0.11	-1.08	0.03	0.113
218273_s_at	PDP1	pyruvate dehyrogenase phosphatase catalytic subunit 1	6.26	6.36	1	-0.10	-1.07	0.01	0.077
1569238_a_at	---	---	3.40	3.53	1	-0.14	-1.10	0.06	0.149
208303_s_at	CRLF2	cytokine receptor-like factor 2	7.30	7.48	1	-0.18	-1.13	0.00	0.056
211653_x_at	AKR1C2	aldo-keto reductase family 1, member C2	6.18	6.31	1	-0.14	-1.10	0.02	0.094
222909_s_at	BAG4	BCL2-associated athanogene 4	6.07	6.22	1	-0.16	-1.11	0.04	0.123
1562194_at	---	---	6.93	7.10	1	-0.18	-1.13	0.05	0.141
220983_s_at	NEAT1	nuclear paraspeckle assembly transcript 1 (non-protein coding)	2.59	2.78	1	-0.19	-1.14	0.01	0.088

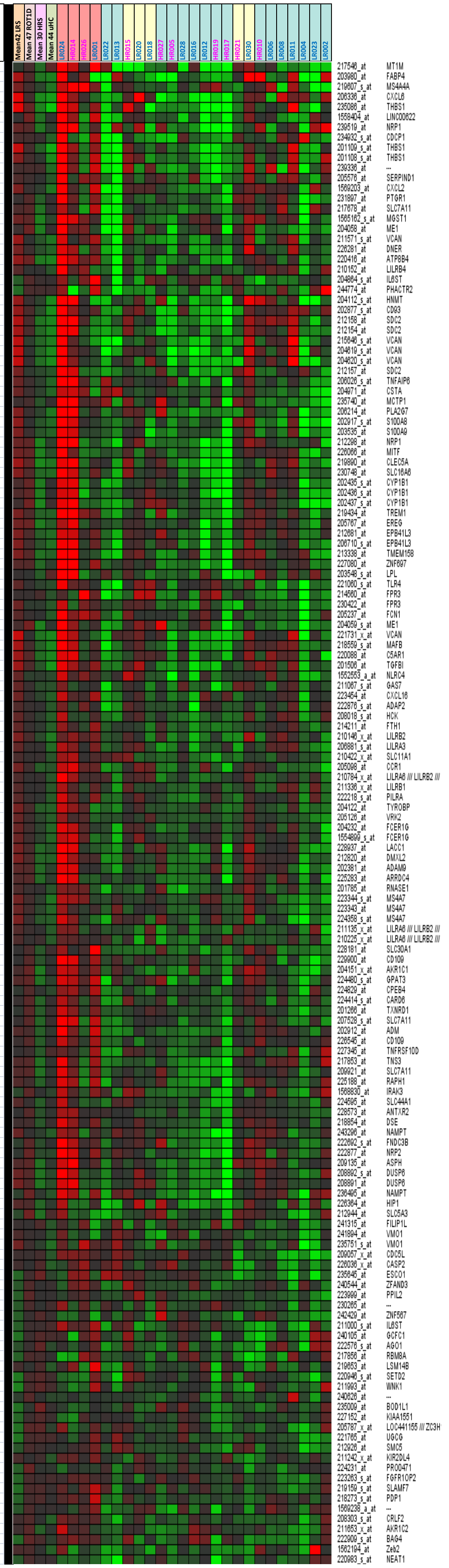

Probeset ID	Gene Symbol	Gene Title	Mean log2 intensity for 21 POST	Mean log2 intensity for 21 PRE	422 FDR <0.2 1=YES 0=NO	Log2 Ratio 21POST vs21PRE	Fold-Change 21POST vs21PRE	p value	FDR
244733_at	---	---	4.65	4.78	1	-0.12	-1.09	0.10	0.194
205966_at	TAF13	TAF13 RNA polymerase II, TATA box binding protein (TBP)-associated factor, 18kDa	3.67	3.92	1	-0.25	-1.19	0.01	0.086
225954_s_at	MIDN	midnolin	6.57	6.87	1	-0.31	-1.24	0.03	0.121
205731_s_at	NCOA2	nuclear receptor coactivator 2	4.50	4.71	1	-0.22	-1.16	0.08	0.175
224549_x_at	---	---	10.53	10.27	1	0.26	1.19	0.01	0.090
210800_at	TIMM8A	translocase of inner mitochondrial membrane 8 homolog A (yeast)	5.78	5.61	1	0.17	1.13	0.08	0.170
220771_at	LINC00328	long intergenic non-protein coding RNA 328	2.58	2.46	1	0.12	1.08	0.09	0.179
230064_at	.OC10537606(	uncharacterized LOC105376066	5.92	5.79	1	0.13	1.09	0.02	0.092
231886_at	.OC10013482:	uncharacterized LOC100134822	5.48	5.21	1	0.27	1.21	0.00	0.050
1561893_at	---	---	3.28	3.11	1	0.18	1.13	0.00	0.056
236650_at	---	---	4.78	4.66	1	0.12	1.08	0.11	0.198
1564430_at	---	---	5.60	5.46	1	0.14	1.10	0.05	0.132
1553185_at	RASEF	RAS and EF-hand domain containing	11.10	10.92	1	0.17	1.13	0.02	0.099
239963_at	---	---	3.45	3.30	1	0.15	1.11	0.04	0.129
1563461_at	---	---	3.15	3.04	1	0.11	1.08	0.08	0.170
1570505_at	ABCB4	ATP binding cassette subfamily B member 4	5.26	5.12	1	0.14	1.10	0.08	0.173
236728_at	LNPEP	leucyl/cystinyl aminopeptidase	5.13	5.00	1	0.13	1.09	0.04	0.124
239798_at	PDK1	pyruvate dehydrogenase kinase, isozyme 1	3.43	3.33	1	0.11	1.08	0.07	0.157
1564093_at	NEK1	NIMA-related kinase 1	4.48	4.24	1	0.24	1.18	0.01	0.085
208575_at	HIST1H3A	histone cluster 1, H3a	3.67	3.49	1	0.17	1.13	0.09	0.179
240015_at	PRKCQ-AS1	PRKCQ antisense RNA 1	4.86	4.76	1	0.10	1.07	0.07	0.167
1561092_at	---	---	3.10	2.98	1	0.12	1.09	0.03	0.113
215162_at	RALGAPA1	Ral GTPase activating protein, alpha subunit 1 (catalytic)	4.78	4.66	1	0.12	1.09	0.11	0.198
232161_x_at	PTPN4	protein tyrosine phosphatase, non-receptor type 4 (megakaryocyte)	3.11	2.94	1	0.17	1.13	0.02	0.100
1562457_at	NCKAP5L	NCK-associated protein 5-like	3.23	3.15	1	0.08	1.06	0.08	0.173
1556461_at	KLF12	---	2.55	2.48	1	0.07	1.05	0.09	0.178
1570115_at	PP12708	---	2.19	2.12	1	0.07	1.05	0.05	0.138
228056_s_at	NAPSB	napsin B aspartic peptidase, pseudogene	5.68	5.56	1	0.12	1.08	0.01	0.085
1560142_at	GRIK2	glutamate receptor, ionotropic, kainate 2	2.20	2.14	1	0.07	1.05	0.01	0.088
238393_at	---	---	2.42	2.31	1	0.11	1.08	0.00	0.072
237877_at	---	---	2.90	2.79	1	0.11	1.08	0.01	0.088
1560755_at	---	---	2.22	2.12	1	0.09	1.07	0.03	0.113
237784_at	SUB1	SUB1 homolog, transcriptional regulator	3.27	3.17	1	0.10	1.07	0.03	0.115
216373_at	TAPT1	transmembrane anterior posterior transformation 1	2.42	2.31	1	0.11	1.08	0.05	0.137
1561600_at	SSH2	---	2.13	2.02	1	0.11	1.08	0.05	0.137
219797_at	MGAT4A	mannosyl (alpha-1,3-)-glycoprotein beta-1,4-N-acetylglucosaminyltransferase, isozyme A	5.64	5.54	1	0.11	1.08	0.09	0.175
1553726_s_at	TBC1D32	TBC1 domain family, member 32	2.56	2.40	1	0.17	1.12	0.00	0.058
1557077_a_at	.OC10537296(	uncharacterized LOC105372966	3.01	2.91	1	0.10	1.07	0.03	0.113
201689_s_at	TPD52	tumor protein D52	6.22	6.12	1	0.09	1.07	0.04	0.125
206398_s_at	CD19	CD19 molecule	7.45	7.33	1	0.12	1.08	0.01	0.085
232181_at	PPARGC1B	peroxisome proliferator-activated receptor gamma, coactivator 1 beta	3.67	3.55	1	0.12	1.08	0.03	0.111
242228_at	BICD1	bicaudal D homolog 1 (Drosophila)	2.75	2.58	1	0.17	1.13	0.00	0.060
1564970_at	SETDB2	SET domain, bifurcated 2	3.84	3.73	1	0.11	1.08	0.10	0.192
224151_s_at	AK3	adenylate kinase 3	4.03	3.88	1	0.15	1.11	0.01	0.085
228055_at	NAPSB	napsin B aspartic peptidase, pseudogene	7.25	7.14	1	0.11	1.08	0.06	0.152
1570022_at	---	---	2.40	2.31	1	0.09	1.07	0.09	0.178
1552633_at	ZNF101	zinc finger protein 101	6.36	6.26	1	0.10	1.07	0.04	0.126
236985_at	---	---	2.94	2.81	1	0.13	1.09	0.04	0.125
1562161_at	---	---	2.69	2.57	1	0.12	1.09	0.07	0.169
232125_at	ZBTB20	zinc finger and BTB domain containing 20	5.31	5.15	1	0.16	1.12	0.11	0.198
229513_at	STRBP	spermatid perinuclear RNA binding protein	6.52	6.37	1	0.14	1.10	0.11	0.199
228905_at	PCM1	pericentriolar material 1	4.18	4.00	1	0.18	1.13	0.04	0.123
1564972_x_at	SETDB2	SET domain, bifurcated 2	4.06	3.83	1	0.23	1.18	0.01	0.092
239867_at	---	---	4.07	3.88	1	0.19	1.14	0.01	0.085
1560225_at	CNR1	cannabinoid receptor 1 (brain)	2.95	2.81	1	0.15	1.11	0.08	0.170
211934_x_at	GANAB	glucosidase, alpha; neutral AB	6.79	6.69	1	0.11	1.08	0.09	0.179
1565692_at	---	---	4.75	4.58	1	0.17	1.12	0.03	0.116
217701_x_at	---	---	3.26	3.04	1	0.22	1.17	0.02	0.098
241723_at	IQGAP2	IQ motif containing GTPase activating protein 2	3.12	3.01	1	0.12	1.08	0.11	0.198
1555317_at	POLK	polymerase (DNA directed) kappa	2.57	2.40	1	0.17	1.13	0.05	0.141
1555199_at	GOSR1	golgi SNAP receptor complex member 1	2.81	2.62	1	0.20	1.15	0.02	0.100
1563077_at	.OC10028905(	uncharacterized LOC100289058	4.13	3.99	1	0.14	1.10	0.06	0.146
1560662_s_at	WHAMMP2	WAS protein homolog associated with actin, golgi membranes and microtubules pseudogene 2	5.13	4.93	1	0.21	1.15	0.00	0.056
1559201_a_at	---	---	6.18	6.05	1	0.13	1.09	0.02	0.092
1566191_at	SUZ12	SUZ12 polycomb repressive complex 2 subunit	3.32	3.14	1	0.18	1.14	0.00	0.072
1560048_at	---	---	2.68	2.62	1	0.06	1.04	0.08	0.171
243910_x_at	CAND1	cullin-associated and neddylation-dissociated 1	5.52	5.39	1	0.12	1.09	0.04	0.126
1561154_at	---	---	2.37	2.21	1	0.16	1.12	0.01	0.077
244766_at	/ NPIPB5 /// S	putative uncharacterized protein LOC641298 /// putative uncharacterized protein LOC641298 /// putative uncharact	6.58	6.45	1	0.13	1.10	0.02	0.093
207115_x_at	MBTD1	mbt domain containing 1	4.02	3.82	1	0.20	1.15	0.00	0.050
232665_x_at	SMURF1	SMAD specific E3 ubiquitin protein ligase 1	4.57	4.34	1	0.23	1.17	0.00	0.056
215070_x_at	R21 /// RABG/	G protein-coupled receptor 21 /// RAB GTPase activating protein 1	3.57	3.41	1	0.15	1.11	0.02	0.095
1560209_at	---	---	2.10	1.94	1	0.16	1.11	0.00	0.056
1558698_at	ZNF264	zinc finger protein 264	3.19	3.07	1	0.12	1.09	0.03	0.114
1564640_at	MGA	MGA, MAX dimerization protein	2.18	2.08	1	0.09	1.07	0.03	0.118
238079_at	TPM3	tropomyosin 3	2.96	2.88	1	0.09	1.06	0.11	0.199
236060_at	METTL14	methyltransferase like 14	3.18	3.01	1	0.18	1.13	0.01	0.086
1559731_x_at	MACROD1	MACRO domain containing 1	2.85	2.69	1	0.16	1.11	0.03	0.115
234112_at	CBLB	---	2.93	2.80	1	0.13	1.09	0.07	0.166
233055_at	---	---	5.40	5.22	1	0.18	1.14	0.02	0.098
216527_at	HCG18	HLA complex group 18 (non-protein coding)(HCG18)	6.52	6.29	1	0.23	1.17	0.00	0.058
217653_x_at	---	---	5.97	5.69	1	0.28	1.21	0.00	0.050
207286_at	CEP135	centrosomal protein 135kDa	2.78	2.54	1	0.24	1.18	0.00	0.050
1569573_at	---	---	2.96	2.69	1	0.26	1.20	0.00	0.056
1570415_at	DDX52	DEAD (Asp-Glu-Ala-Asp) box polypeptide 52	2.86	2.59	1	0.27	1.20	0.00	0.064
215900_at	---	---	2.99	2.86	1	0.13	1.09	0.08	0.170
1558418_at	PIAS1	---	3.52	3.38	1	0.14	1.10	0.03	0.121
243899_at	L17A /// ARL1	ADP-ribosylation factor like GTPase 17A /// ADP-ribosylation factor like GTPase 17B	6.48	6.32	1	0.16	1.11	0.01	0.086
1560112_at	WDFY2	WD repeat and FYVE domain containing 2	3.13	2.98	1	0.16	1.11	0.01	0.092
1556828_at	---	---	2.52	2.39	1	0.13	1.10	0.01	0.092
1560349_at	---	---	2.75	2.55	1	0.21	1.15	0.00	0.064
1564438_at	---	---	2.95	2.84	1	0.11	1.08	0.09	0.179
1559580_at	LRRC39	leucine rich repeat containing 39	3.11	2.95	1	0.16	1.12	0.01	0.085
222319_at	---	---	3.91	3.78	1	0.13	1.09	0.09	0.175
1563364_at	---	---	3.22	2.96	1	0.26	1.20	0.00	0.058
217704_x_at	---	---	3.45	3.21	1	0.24	1.18	0.00	0.050
1559039_at	DHX36	DEAH (Asp-Glu-Ala-His) box polypeptide 36	5.19	5.02	1	0.17	1.13	0.01	0.085
215128_at	---	---	4.24	4.02	1	0.22	1.16	0.00	0.072
223036_at	FARSB	phenylalanyl-tRNA synthetase beta subunit	2.46	2.32	1	0.14	1.10	0.02	0.095
242126_at	---	---	2.93	2.77	1	0.16	1.12	0.01	0.085
235551_at	---	---	3.32	3.08	1	0.25	1.19	0.00	0.058
220216_at	C8orf44	chromosome 8 open reading frame 44	4.36	4.18	1	0.19	1.14	0.02	0.092
237377_at	---	---	3.64	3.50	1	0.14	1.10	0.04	0.121
1568858_at	---	---	2.77	2.63	1	0.14	1.10	0.03	0.121
1561128_at	---	---	3.40	3.24	1	0.16	1.12	0.05	0.132
211459_at	---	---	2.43	2.26	1	0.17	1.13	0.06	0.147
228304_at	RBM43	RNA binding motif protein 43	4.29	4.08	1	0.21	1.16	0.02	0.094
243655_x_at	---	---	2.69	2.54	1	0.15	1.11	0.04	0.126
233239_at	---	---	4.84	4.61	1	0.23	1.18	0.04	0.123
208077_at	---	---	3.78	3.61	1	0.17	1.12	0.09	0.177
206169_x_at	ZC3H7B	zinc finger CCCH-type containing 7B	8.89	8.57	1	0.32	1.25	0.00	0.072
1558792_x_at	AP2A1	adaptor-related protein complex 2, alpha 1 subunit	6.05	5.70	1	0.35	1.27	0.00	0.056
1569472_s_at	TTC3	tetratricopeptide repeat domain 3	5.80	5.53	1	0.27	1.20	0.04	0.126
1557197_a_at	LGALS3	lectin, galactoside-binding, soluble, 3	3.15	2.86	1	0.29	1.22	0.02	0.099
215175_at	PCNX	pecanex homolog (Drosophila)	5.17	4.92	1	0.25	1.19	0.08	0.172
215648_at	---	---	4.68	4.34	1	0.34	1.27	0.01	0.085
234148_at	---	---	4.53	4.19	1	0.34	1.27	0.02	0.099
1561733_at	---	---	3.74	3.42	1	0.32	1.25	0.00	0.072
206088_at	37A2 /// LRRC	leucine rich repeat containing 37, member A2 /// leucine rich repeat containing 37, member A3	4.14	3.78	1	0.36	1.29	0.00	0.072
240246_at	FRG1JP	FSHD region gene 1 family member J, pseudogene	4.54	4.26	1	0.27	1.21	0.01	0.085
237232_at	---	---	3.39	3.12	1	0.28	1.21	0.01	0.085
244010_at	---	---	5.34	5.04	1	0.30	1.23	0.03	0.113
233554_at	---	---	3.93	3.62	1	0.31	1.24	0.02	0.094
1554948_at	---	---	4.32	3.98	1	0.34	1.27	0.02	0.092
233824_at	---	---	4.31	4.04	1	0.27	1.21	0.04	0.123
1560800_at	---	---	2.91	2.67	1	0.23	1.17	0.03	0.111
232365_at	SIAH1	siah E3 ubiquitin protein ligase 1	2.78	2.55	1	0.23	1.17	0.01	0.085
220900_at	---	---	2.61	2.27	1	0.33	1.26	0.00	0.064
232535_at	RSBN1L	round spermatid basic protein 1-like	3.54	3.34	1	0.20	1.15	0.03	0.114
238723_at	ATXN3	ataxin 3	4.39	4.14	1	0.25	1.19	0.02	0.092
205727_at	TEP1	telomerase-associated protein 1	3.98	3.76	1	0.21	1.16	0.04	0.129
216121_at	---	---	4.34	4.07	1	0.27	1.21	0.01	0.085
233931_at	---	---	5.89	5.54	1	0.36	1.28	0.00	0.057
220338_at	RALGPS2	Ral GEF with PH domain and SH3 binding motif 2	4.43	4.12	1	0.31	1.24	0.00	0.064
216850_at	SNRPN	small nuclear ribonucleoprotein polypeptide N	3.77	3.51	1	0.27	1.20	0.00	0.056
215398_at	---	---	4.55	4.39	1	0.16	1.12	0.04	0.125
1562467_at	PPP3CA	---	3.44	3.17	1	0.27	1.20	0.00	0.064
238931_at	METTL16	methyltransferase like 16	3.84	3.67	1	0.17	1.13	0.08	0.171
244473_at	---	---	4.75	4.53	1	0.23	1.17	0.07	0.168
1569540_at	---	---	3.50	3.18	1	0.32	1.25	0.04	0.125
1562270_at	ARHGEF7	Rho guanine nucleotide exchange factor 7	2.75	2.52	1	0.23	1.17	0.04	0.125
1564820_at	---	---	4.12	3.84	1	0.29	1.22	0.05	0.132
239936_at	DLEU2	deleted in lymphocytic leukemia 2 (non-protein coding)	2.68	2.53	1	0.15	1.11	0.08	0.172
207686_s_at	CASP8	caspase 8, apoptosis-related cysteine peptidase	7.09	6.91	1	0.18	1.13	0.07	0.169
215252_at	---	---	3.77	3.54	1	0.23	1.17	0.03	0.116
1570249_x_at	---	---	4.43	4.25	1	0.18	1.13	0.05	0.138
239151_at	'5 /// LOC1027	BMS1 ribosome biogenesis factor pseudogene 5 /// uncharacterized LOC102724455	5.52	5.31	1	0.21	1.16	0.01	0.092
1560014_s_at	2724985 /// P	pyridoxal-dependent decarboxylase domain-containing protein 1 /// pyridoxal-dependent decarboxylase domain co	4.39	4.19	1	0.20	1.15	0.04	0.125

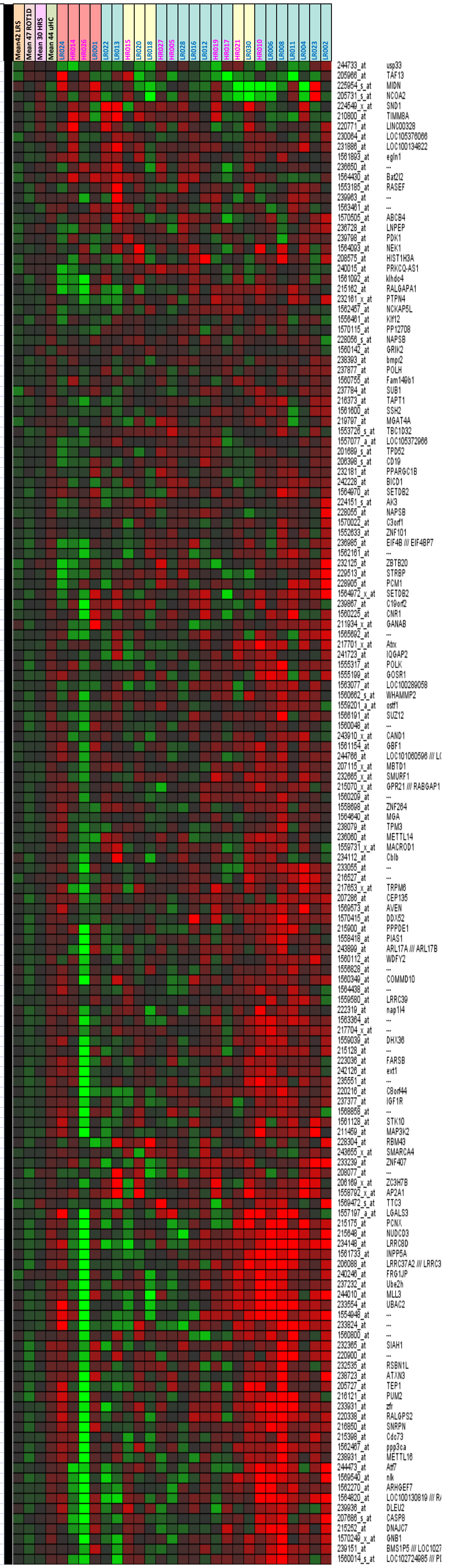

Probeset ID	Gene Symbol	Gene Title	Mean log2 intensity for 21 POST	Mean log2 intensity for 21 PRE	422 FDR <0.2 1=YES 0=NO	Log2 Ratio 21POST vs21PRE	Fold-Change 21POST vs21PRE	p value	FDR
1557409_at	---	---	5.20	5.03	1	0.16	1.12	0.05	0.137
215462_at	PLK3	polo-like kinase 3	5.73	5.50	1	0.23	1.18	0.01	0.086
215474_at	nsmce2	---	3.60	3.32	1	0.28	1.21	0.01	0.085
239008_at	---	---	4.17	3.90	1	0.27	1.20	0.03	0.116
233231_at	---	---	3.64	3.44	1	0.20	1.15	0.08	0.170
214740_at	POLR2J3	polymerase (RNA) II (DNA directed) polypeptide J3	4.33	4.13	1	0.20	1.15	0.09	0.179
242549_at	PRKD3	protein kinase D3	4.51	4.29	1	0.21	1.16	0.08	0.170
1559139_at	NOC2L	NOC2-like nucleolar associated transcriptional repressor	4.60	4.38	1	0.22	1.17	0.11	0.199
235061_at	PPM1K	protein phosphatase, Mg2+/Mn2+ dependent, 1K	7.12	6.85	1	0.28	1.21	0.00	0.072
213650_at	GA8A /// GOL	golgin A8 family, member A /// golgin A8 family, member B	7.50	7.29	1	0.21	1.16	0.10	0.187
208154_at	---	---	3.14	2.80	1	0.34	1.27	0.01	0.083
1570248_at	---	---	3.44	3.19	1	0.25	1.19	0.08	0.170
232330_at	COA1	cytochrome c oxidase assembly factor 1 homolog	6.84	6.46	1	0.39	1.31	0.01	0.085
1562255_at	SYTL3	synaptotagmin-like 3	4.91	4.72	1	0.19	1.14	0.10	0.193
244418_at	---	---	3.51	3.34	1	0.17	1.13	0.09	0.182
242176_at	MEF2A	myocyte enhancer factor 2A	3.47	3.32	1	0.15	1.11	0.06	0.146
234643_x_at	---	---	5.13	4.90	1	0.23	1.17	0.01	0.085
233400_at	---	---	4.63	4.45	1	0.19	1.14	0.10	0.185
236153_at	---	---	4.53	4.39	1	0.14	1.11	0.05	0.132
239661_at	---	---	4.16	3.98	1	0.18	1.13	0.04	0.121
233713_at	---	---	5.71	5.55	1	0.16	1.12	0.03	0.113
1555812_a_at	ARHGDIB	Rho GDP dissociation inhibitor (GDI) beta	9.00	8.87	1	0.13	1.09	0.08	0.170
215118_s_at	IGH	immunoglobulin heavy locus	6.19	6.02	1	0.17	1.13	0.03	0.113
219980_at	ABHD18	abhydrolase domain containing 18	5.12	4.91	1	0.21	1.16	0.05	0.132
232346_at	LOC388692	uncharacterized LOC388692	6.17	5.94	1	0.23	1.17	0.07	0.168
1568983_a_at	GABPB1-AS1	GABPB1 antisense RNA 1(GABPB1-AS1)	5.47	5.23	1	0.24	1.18	0.06	0.144
1560814_a_at	C15orf57	chromosome 15 open reading frame 57	4.16	3.90	1	0.26	1.20	0.06	0.146
220467_at	---	---	7.64	7.32	1	0.32	1.25	0.01	0.085
217671_at	---	---	6.49	6.31	1	0.19	1.14	0.05	0.138
242241_x_at	---	---	4.96	4.71	1	0.25	1.19	0.00	0.064
215626_at	---	---	3.45	3.29	1	0.16	1.12	0.09	0.179
215659_at	GSDMB	gasdermin B	3.09	2.88	1	0.20	1.15	0.02	0.103
1560391_at	PIGL	phosphatidylinositol glycan anchor biosynthesis class L	3.41	3.27	1	0.13	1.10	0.09	0.179
1557360_at	LRPPRC	leucine-rich pentatricopeptide repeat containing	6.37	6.24	1	0.13	1.09	0.09	0.179
243796_at	---	---	5.65	5.54	1	0.10	1.07	0.11	0.197
1555379_at	FAM159A	family with sequence similarity 159, member A	3.27	3.14	1	0.13	1.09	0.05	0.137
242423_x_at	ZNF782	zinc finger protein 782	3.32	3.16	1	0.16	1.12	0.03	0.117
1564753_at	---	---	4.35	4.14	1	0.21	1.16	0.02	0.100
1569041_at	---	---	4.92	4.70	1	0.22	1.17	0.04	0.126
233630_at	CDS2	CDP-diacylglycerol synthase 2	4.57	4.40	1	0.17	1.12	0.08	0.171
243876_at	---	---	5.06	4.86	1	0.20	1.15	0.06	0.145
1554769_at	ZNF785	zinc finger protein 785	3.75	3.54	1	0.21	1.16	0.06	0.143
239649_at	---	---	5.29	5.13	1	0.16	1.12	0.08	0.170
1563051_at	OSBP	oxysterol binding protein	3.82	3.64	1	0.18	1.13	0.07	0.168
1569392_at	TECR	trans-2,3-enoyl-CoA reductase	3.27	3.10	1	0.18	1.13	0.06	0.143
1556849_at	---	---	2.56	2.37	1	0.18	1.14	0.08	0.170
241207_at	---	---	2.74	2.54	1	0.20	1.15	0.04	0.130
242490_at	---	---	5.08	4.87	1	0.21	1.16	0.08	0.171
217594_at	ZCCHC11	zinc finger, CCHC domain containing 11	3.64	3.38	1	0.26	1.19	0.03	0.113
1560443_at	SMCHD1	---	6.38	6.08	1	0.31	1.24	0.02	0.092
232338_at	ZNF431	zinc finger protein 431	4.35	4.11	1	0.25	1.19	0.07	0.159
221191_at	G3L1 /// STAG	stromal antigen 3-like 1 (pseudogene) /// stromal antigen 3-like 3 (pseudogene)	5.76	5.54	1	0.22	1.16	0.08	0.171
1557996_at	---	---	6.84	6.54	1	0.30	1.23	0.03	0.113
1558220_at	---	---	5.62	5.30	1	0.32	1.25	0.03	0.121
241860_at	---	---	4.92	4.68	1	0.24	1.18	0.10	0.187
1560013_at	2724985 /// P	pyridoxal-dependent decarboxylase domain-containing protein 1 /// pyridoxal-dependent decarboxylase domain co	4.12	3.92	1	0.20	1.15	0.09	0.181
239453_at	---	---	6.24	5.99	1	0.25	1.19	0.08	0.172
1565889_at	---	---	6.03	5.77	1	0.26	1.20	0.06	0.152
1558802_at	TM2D1	TM2 domain containing 1	3.29	3.07	1	0.22	1.16	0.06	0.146
1559490_at	LRCH3	leucine-rich repeats and calponin homology (CH) domain containing 3	6.58	6.29	1	0.28	1.22	0.04	0.123
239937_at	ZNF207	zinc finger protein 207	6.99	6.76	1	0.23	1.17	0.04	0.125
215012_at	ZNF451	zinc finger protein 451	6.56	6.33	1	0.23	1.17	0.05	0.132
231848_x_at	ZNF207	zinc finger protein 207	8.16	7.91	1	0.25	1.19	0.04	0.125
238769_at	---	---	5.29	5.03	1	0.26	1.20	0.09	0.176
232521_at	PCSK7	proprotein convertase subtilisin/kexin type 7	6.15	5.90	1	0.25	1.19	0.09	0.179
1569527_at	---	---	4.09	3.81	1	0.29	1.22	0.06	0.152
242772_x_at	---	---	4.82	4.43	1	0.40	1.32	0.01	0.086
216983_s_at	ZNF224	zinc finger protein 224	5.40	5.16	1	0.24	1.18	0.08	0.170
1569180_at	---	---	5.80	5.43	1	0.37	1.29	0.02	0.092
218362_s_at	DIS3	DIS3 homolog, exosome endoribonuclease and 3'-5' exoribonuclease	4.86	4.61	1	0.25	1.19	0.02	0.111
1557580_at	---	---	3.42	3.15	1	0.28	1.21	0.01	0.088
242527_at	---	---	4.20	4.00	1	0.20	1.15	0.06	0.155
215375_x_at	LRRFIP1	leucine rich repeat (in FLII) interacting protein 1	5.15	4.95	1	0.20	1.15	0.05	0.141
1562412_at	GPR89B	G protein-coupled receptor 89B	4.30	4.05	1	0.25	1.19	0.03	0.117
232759_at	---	---	3.98	3.65	1	0.34	1.26	0.01	0.085
216765_at	MAP2K5	mitogen-activated protein kinase kinase 5	4.14	3.89	1	0.25	1.19	0.04	0.125
1562028_at	CCND3	cyclin D3	5.57	5.26	1	0.31	1.24	0.02	0.094
232002_at	---	---	4.84	4.58	1	0.26	1.20	0.01	0.092
240720_at	---	---	3.54	3.31	1	0.24	1.18	0.01	0.085
1562238_at	USPL1	ubiquitin specific peptidase like 1	3.16	2.89	1	0.28	1.21	0.00	0.062
1557581_x_at	---	---	2.88	2.62	1	0.26	1.20	0.02	0.095
231979_at	GABPB1-AS1	GABPB1 antisense RNA 1(GABPB1-AS1)	3.45	3.24	1	0.21	1.16	0.01	0.088
215170_s_at	CEP152	centrosomal protein 152kDa	3.44	3.16	1	0.28	1.22	0.01	0.079
210858_x_at	ATM	ATM serine/threonine kinase	7.12	6.91	1	0.21	1.16	0.05	0.141
208442_s_at	ATM	ATM serine/threonine kinase	5.78	5.42	1	0.36	1.28	0.03	0.113
242974_at	CD47	CD47 molecule(CD47)	5.79	5.45	1	0.34	1.27	0.00	0.074
205594_at	ZNF652	zinc finger protein 652	4.95	4.63	1	0.32	1.25	0.02	0.095
215029_at	AKIRIN1	akirin 1	4.58	4.27	1	0.31	1.24	0.10	0.191
239405_at	---	---	4.29	4.04	1	0.25	1.19	0.08	0.170
215558_at	UBR2	ubiquitin protein ligase E3 component n-recognin 2	4.26	3.87	1	0.39	1.31	0.01	0.088
242797_x_at	---	---	4.14	3.75	1	0.40	1.32	0.01	0.079
207474_at	SNRK	SNF related kinase	4.87	4.53	1	0.34	1.27	0.05	0.132
1557270_at	---	---	4.82	4.36	1	0.46	1.37	0.00	0.056
1570571_at	CCDC91	coiled-coil domain containing 91	5.04	4.61	1	0.43	1.34	0.01	0.079
219341_at	CLN8	ceroid-lipofuscinosis, neuronal 8	3.94	3.65	1	0.29	1.22	0.01	0.088
239258_at	---	---	5.47	5.18	1	0.29	1.22	0.07	0.161
1569312_at	---	---	4.69	4.38	1	0.30	1.23	0.06	0.141
207735_at	RNF125	ring finger protein 125, E3 ubiquitin protein ligase	6.05	5.75	1	0.31	1.24	0.07	0.167
243134_at	---	---	5.58	5.14	1	0.44	1.36	0.02	0.094
242691_at	---	---	4.36	4.10	1	0.27	1.20	0.08	0.173
232412_at	FBXL20	F-box and leucine-rich repeat protein 20	3.62	3.27	1	0.35	1.27	0.03	0.113
1565743_at	N4BP2L2	NEDD4 binding protein 2-like 2	4.24	3.70	1	0.54	1.45	0.00	0.056
1562283_at	---	---	2.98	2.58	1	0.39	1.31	0.01	0.085
222412_s_at	SSR3	signal sequence receptor, gamma (translocon-associated protein gamma)	6.30	5.95	1	0.35	1.28	0.05	0.136
1556658_a_at	---	---	6.19	5.77	1	0.42	1.34	0.01	0.085
1559449_a_at	ZNF254	zinc finger protein 254	4.83	4.32	1	0.51	1.42	0.00	0.056
1569538_at	---	---	3.77	3.34	1	0.43	1.35	0.00	0.056
235030_at	NXPE3	neurexophilin and PC-esterase domain family, member 3	4.71	4.40	1	0.30	1.24	0.03	0.111
1556657_at	---	---	5.32	5.03	1	0.30	1.23	0.01	0.092
1557737_s_at	NKTR	natural killer cell triggering receptor	4.68	4.22	1	0.46	1.37	0.00	0.064
1552867_at	---	---	3.13	2.82	1	0.31	1.24	0.06	0.153
216766_at	---	---	4.34	4.00	1	0.34	1.27	0.05	0.132
220458_at	---	---	3.64	3.28	1	0.36	1.28	0.02	0.092
232295_at	GFM1	G elongation factor, mitochondrial 1	3.09	2.81	1	0.28	1.21	0.01	0.083
221771_s_at	MPHOSPH8	M-phase phosphoprotein 8	6.94	6.66	1	0.28	1.22	0.11	0.199
239635_at	RBM14	RNA binding motif protein 14	5.85	5.51	1	0.34	1.26	0.05	0.141
216022_at	---	---	5.30	5.02	1	0.28	1.21	0.10	0.195
1568943_at	INPP5D/SHIP1	inositol polyphosphate-5-phosphatase D	6.00	5.56	1	0.44	1.36	0.01	0.077
215392_at	---	---	4.77	4.42	1	0.35	1.27	0.10	0.185
1557551_at	---	---	6.30	5.94	1	0.36	1.28	0.08	0.173
1558515_at	FTX	FTX transcript, XIST regulator (non-protein coding)	6.22	5.79	1	0.43	1.34	0.07	0.164
215123_at	IPIPA3 /// NPI	nuclear pore complex-interacting protein family member B4-like /// uncharacterized LOC105376851 /// nuclear pore	7.07	6.58	1	0.49	1.40	0.02	0.099
1558710_at	ARIH1	---	4.73	4.43	1	0.30	1.23	0.09	0.179
210556_at	NFATC3	nuclear factor of activated T-cells, cytoplasmic, calcineurin-dependent 3	5.03	4.62	1	0.41	1.33	0.04	0.125
216813_at	---	---	5.39	4.86	1	0.53	1.44	0.03	0.113
235028_at	---	---	7.11	6.67	1	0.44	1.36	0.10	0.187
216598_s_at	CCL2	chemokine (C-C motif) ligand 2	9.95	10.14	0	-0.19	-1.14	0.24	0.292
206569_at	IL24	interleukin 24	6.61	6.85	0	-0.25	-1.19	0.15	0.231
1553142_at	LACC1	laccase (multicopper oxidoreductase) domain containing 1	4.18	4.37	0	-0.19	-1.14	0.15	0.231
202878_s_at	CD93	CD93 molecule	8.40	8.56	0	-0.16	-1.12	0.23	0.286
201110_s_at	THBS1	thrombospondin 1	10.14	10.31	0	-0.17	-1.13	0.13	0.210
218856_at	TNFRSF21	tumor necrosis factor receptor superfamily, member 21	4.73	4.91	0	-0.18	-1.13	0.14	0.221
217513_at	MILR1	mast cell immunoglobulin-like receptor 1	3.27	3.40	0	-0.13	-1.09	0.18	0.256
228824_s_at	PTGR1	prostaglandin reductase 1	4.57	4.67	0	-0.10	-1.07	0.23	0.284
206025_s_at	TNFAIP6	tumor necrosis factor, alpha-induced protein 6	8.89	9.04	0	-0.15	-1.11	0.12	0.204
211924_s_at	PLAUR	plasminogen activator, urokinase receptor	8.91	9.05	0	-0.14	-1.10	0.13	0.216
207075_at	NLRP3	NLR family, pyrin domain containing 3	7.18	7.29	0	-0.11	-1.08	0.19	0.259
219558_at	ATP13A3	ATPase type 13A3	8.17	8.27	0	-0.10	-1.07	0.17	0.241
204924_at	TLR2	toll-like receptor 2	9.63	9.71	0	-0.08	-1.06	0.29	0.313
205067_at	IL1B	interleukin 1 beta	12.78	12.86	0	-0.08	-1.06	0.24	0.292
217996_at	PHLDA1	pleckstrin homology-like domain, family A, member 1	9.42	9.52	0	-0.10	-1.07	0.17	0.241
217997_at	PHLDA1	pleckstrin homology-like domain, family A, member 1	8.13	8.27	0	-0.14	-1.10	0.12	0.208
210896_s_at	ASPH	aspartate beta-hydroxylase	6.58	6.69	0	-0.11	-1.08	0.19	0.256
213524_s_at	G0S2	G0/G1 switch 2	9.50	9.57	0	-0.07	-1.05	0.25	0.292
202833_s_at	SERPINA1	serpin peptidase inhibitor, clade A (alpha-1 antiproteinase, antitrypsin), member 1	7.73	7.77	0	-0.04	-1.03	0.43	0.374
1552703_s_at	ARD16 /// CAS	caspase recruitment domain family, member 16 /// caspase 1	9.88	9.92	0	-0.04	-1.03	0.49	0.400
235518_at	SLC8A1	solute carrier family 8 (sodium/calcium exchanger), member 1	3.96	4.04	0	-0.08	-1.06	0.26	0.299
214681_at	GK	glycerol kinase	6.96	7.05	0	-0.09	-1.06	0.19	0.258

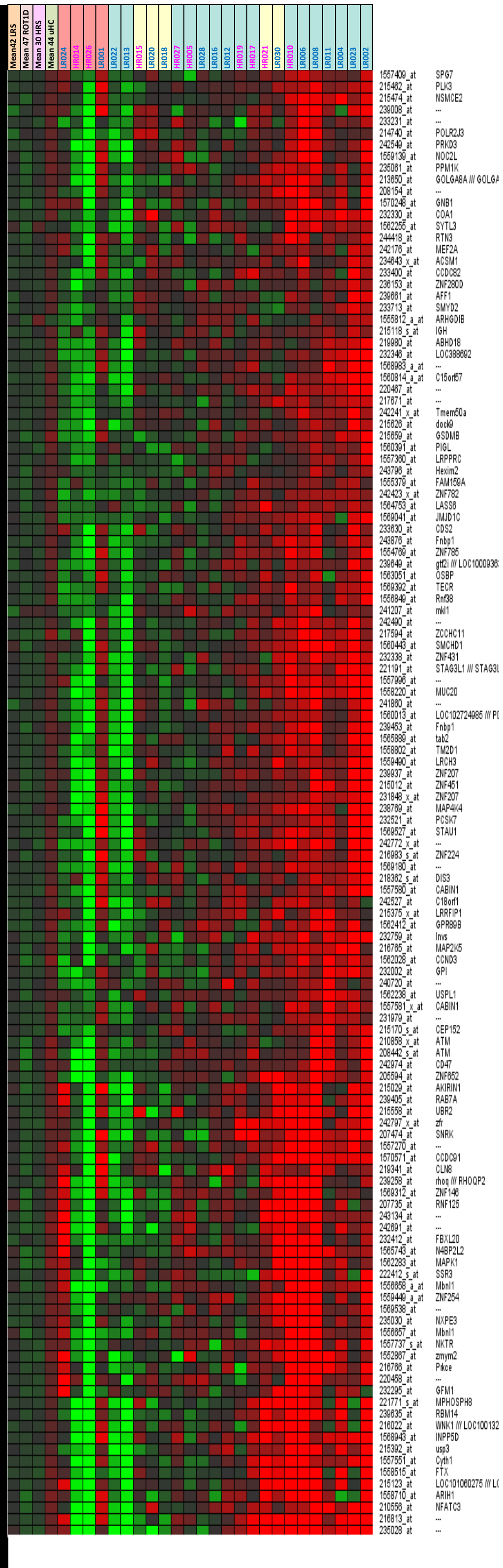

Probeset ID	Gene Symbol	Gene Title	Mean log2 intensity for 21 POST	Mean log2 intensity for 21 PRE	422 FDR <0.2 1=YES 0=NO	Log2 Ratio 21POST vs21PRE	Fold-Change 21POST vs21PRE	p value	FDR
222693_at	FNDC3B	fibronectin type III domain containing 3B	5.94	6.06	0	-0.12	-1.08	0.20	0.265
202600_s_at	NRIP1	nuclear receptor interacting protein 1	8.00	8.08	0	-0.08	-1.05	0.37	0.350
221802_s_at	SHTN1	shootin 1	5.68	5.81	0	-0.13	-1.10	0.15	0.228
227314_at	ITGA2	integrin, alpha 2 (CD49B, alpha 2 subunit of VLA-2 receptor)	5.20	5.31	0	-0.11	-1.08	0.29	0.311
1559910_at	---	---	8.97	9.14	0	-0.18	-1.13	0.11	0.201
1553313_s_at	SLC5A3	solute carrier family 5 (sodium/myo-inositol cotransporter), member 3	5.90	6.05	0	-0.15	-1.11	0.13	0.213
210873_x_at	C3A /// APOBI	apolipoprotein B mRNA editing enzyme, catalytic polypeptide-like 3A /// APOBEC3A and APOBEC3B deletion hybrid	7.68	7.80	0	-0.11	-1.08	0.11	0.201
206028_s_at	MERTK	MER proto-oncogene, tyrosine kinase	2.57	2.70	0	-0.12	-1.09	0.17	0.247
207697_x_at	LILRB2	leukocyte immunoglobulin-like receptor, subfamily B (with TM and ITIM domains), member 2	7.00	7.09	0	-0.09	-1.07	0.14	0.224
219788_at	PILRA	paired immunoglobin-like type 2 receptor alpha	6.03	6.13	0	-0.10	-1.07	0.14	0.220
201360_at	CST3	cystatin C	7.30	7.38	0	-0.09	-1.06	0.12	0.202
215767_at	ZNF804A	zinc finger protein 804A	3.57	3.64	0	-0.07	-1.05	0.38	0.351
227632_at	TBC1D24	TBC1 domain family, member 24	2.90	3.00	0	-0.10	-1.07	0.13	0.214
1569431_at	PAFAH1B2	platelet-activating factor acetylhydrolase 1b, catalytic subunit 2 (30kDa)	2.55	2.62	0	-0.07	-1.05	0.22	0.276
241955_at	HECTD1	HECT domain containing E3 ubiquitin protein ligase 1	7.48	7.55	0	-0.07	-1.05	0.15	0.231
200628_s_at	WARS	tryptophanyl-tRNA synthetase	8.78	8.85	0	-0.07	-1.05	0.35	0.339
218228_s_at	TNKS2	tankyrase, TRF1-interacting ankyrin-related ADP-ribose polymerase 2	7.80	7.83	0	-0.03	-1.02	0.45	0.378
206668_s_at	SCAMP1	secretory carrier membrane protein 1	5.52	5.47	0	0.05	1.03	0.19	0.256
212398_at	RDX	radixin	6.86	6.91	0	-0.05	-1.04	0.47	0.389
230466_s_at	RASSF3	Ras association (RalGDS/AF-6) domain family member 3	6.99	7.12	0	-0.13	-1.09	0.12	0.202
239871_at	CLTC	clathrin, heavy chain (Hc)	2.33	2.40	0	-0.08	-1.05	0.15	0.232
1554442_at	BEST1	bestrophin 1	5.87	5.94	0	-0.07	-1.05	0.28	0.305
200730_s_at	PTP4A1	protein tyrosine phosphatase type IVA, member 1	7.96	8.02	0	-0.06	-1.04	0.40	0.363
205809_s_at	WASL	Wiskott-Aldrich syndrome-like	6.26	6.39	0	-0.13	-1.09	0.14	0.219
237332_at	MON2	MON2 homolog, regulator of endosome-to-Golgi trafficking	4.34	4.47	0	-0.13	-1.09	0.13	0.215
238470_at	SYS1	Sys1 golgi trafficking protein	4.49	4.40	0	0.09	1.06	0.21	0.274
1565595_at	---	---	3.95	3.88	0	0.07	1.05	0.27	0.304
234459_at	PPHLN1	periphilin 1	3.41	3.33	0	0.08	1.05	0.32	0.328
1559762_at	---	---	2.26	2.19	0	0.07	1.05	0.21	0.271
1564639_at	.OC10029132:	uncharacterized LOC100291323	3.09	3.06	0	0.04	1.03	0.40	0.359
232257_s_at	---	---	4.20	4.05	0	0.15	1.11	0.14	0.219
241863_x_at	TTC14	tetratricopeptide repeat domain 14	4.10	4.02	0	0.08	1.06	0.21	0.270
203940_s_at	VASH1	vasohibin 1	4.96	4.85	0	0.11	1.08	0.22	0.276
239278_at	BCAS4	breast carcinoma amplified sequence 4	5.28	5.14	0	0.13	1.10	0.23	0.283
1559455_at	---	---	4.20	3.99	0	0.21	1.16	0.12	0.208
1569263_at	---	---	4.49	4.33	0	0.17	1.12	0.11	0.207
236778_at	---	---	4.85	4.76	0	0.08	1.06	0.20	0.265
206983_at	CCR6	chemokine (C-C motif) receptor 6	6.49	6.42	0	0.07	1.05	0.30	0.319
241680_at	---	---	4.50	4.42	0	0.08	1.05	0.27	0.302
205267_at	POU2AF1	POU class 2 associating factor 1	8.99	8.93	0	0.07	1.05	0.33	0.333
214705_at	INADL	InaD-like (Drosophila)	3.09	2.96	0	0.13	1.09	0.13	0.214
202940_at	)5369600 /// !	uncharacterized LOC105369600 /// WNK lysine deficient protein kinase 1	4.95	4.82	0	0.14	1.10	0.14	0.224
217534_at	FAM49B	family with sequence similarity 49, member B	5.24	5.04	0	0.21	1.15	0.20	0.265
224031_at	STK17A	---	4.06	3.92	0	0.15	1.11	0.12	0.203
215390_at	---	---	4.72	4.49	0	0.24	1.18	0.14	0.222
1405_i_at	CCL5	chemokine (C-C motif) ligand 5	10.63	10.61	0	0.02	1.02	0.67	0.454
1552417_a_at	NEDD1	neural precursor cell expressed, developmentally down-regulated 1	5.70	5.70	0	0.00	-1.00	0.98	0.540
1552621_at	.R2J2 /// POLF	polymerase (RNA) II (DNA directed) polypeptide J2 /// polymerase (RNA) II (DNA directed) polypeptide J3	8.10	7.80	0	0.30	1.23	0.12	0.202
1552627_a_at	ARHGAP5	Rho GTPase activating protein 5	4.05	4.14	0	-0.10	-1.07	0.13	0.216
1552787_at	HELB	helicase (DNA) B	4.54	4.40	0	0.14	1.10	0.23	0.281
1552788_a_at	HELB	helicase (DNA) B	5.44	5.39	0	0.05	1.03	0.44	0.377
1552978_a_at	SCAMP1	secretory carrier membrane protein 1	6.22	6.20	0	0.03	1.02	0.73	0.468
1553252_a_at	BRWD3	bromodomain and WD repeat domain containing 3	6.99	6.98	0	0.01	1.01	0.85	0.502
1553265_at	SLC23A3	solute carrier family 23, member 3	2.75	2.68	0	0.07	1.05	0.15	0.227
1553267_a_at	CNOT6L	CCR4-NOT transcription complex subunit 6-like	4.44	4.47	0	-0.03	-1.02	0.73	0.468
1553396_a_at	CCDC13	coiled-coil domain containing 13	4.46	4.58	0	-0.12	-1.09	0.19	0.259
1553575_at	ND6	NADH dehydrogenase, subunit 6 (complex I)	10.35	10.31	0	0.04	1.03	0.81	0.492
1554057_at	ASH1L-AS1	ASH1L antisense RNA 1	5.79	5.77	0	0.03	1.02	0.65	0.450
1554152_a_at	OGDH	oxoglutarate (alpha-ketoglutarate) dehydrogenase (lipoamide)	4.00	3.86	0	0.14	1.10	0.25	0.293
1554250_s_at	TRIM73	tripartite motif containing 73	6.54	6.39	0	0.16	1.11	0.24	0.292
1554306_at	ITPKB	inositol-trisphosphate 3-kinase B	2.95	2.91	0	0.03	1.02	0.51	0.409
1554478_a_at	HEATR3	HEAT repeat containing 3	3.61	3.63	0	-0.02	-1.01	0.66	0.451
1554503_a_at	OSCAR	osteoclast associated, immunoglobulin-like receptor	3.71	3.77	0	-0.05	-1.04	0.59	0.435
1554670_at	GGA1	golgi-associated, gamma adaptin ear containing, ARF binding protein 1	4.58	4.42	0	0.16	1.12	0.27	0.303
1554770_x_at	ZNF785	zinc finger protein 785	3.17	3.04	0	0.13	1.10	0.31	0.323
1554910_at	PRKD3	protein kinase D3	3.41	3.43	0	-0.02	-1.02	0.77	0.481
1554997_a_at	PTGS2	prostaglandin-endoperoxide synthase 2 (prostaglandin G/H synthase and cyclooxygenase)	8.36	8.44	0	-0.08	-1.06	0.65	0.450
1555116_s_at	SLC11A1	solute carrier family 11 (proton-coupled divalent metal ion transporter), member 1	2.89	2.83	0	0.06	1.04	0.26	0.299
1555167_s_at	NAMPT	nicotinamide phosphoribosyltransferase	8.88	8.95	0	-0.07	-1.05	0.29	0.315
1555594_a_at	MBNL1	muscleblind like splicing regulator 1	6.26	6.27	0	-0.01	-1.01	0.91	0.523
1555730_a_at	CFL1	cofilin 1 (non-muscle)	8.30	8.05	0	0.25	1.19	0.29	0.315
1555733_s_at	AP1S3	adaptor-related protein complex 1 sigma 3 subunit	5.21	5.23	0	-0.02	-1.01	0.85	0.504
1555977_at	MYL12A	myosin light chain 12A	6.03	6.00	0	0.04	1.03	0.43	0.374
1556202_at	SRGAP2	SLIT-ROBO Rho GTPase activating protein 2	4.11	4.23	0	-0.12	-1.09	0.41	0.365
1556226_at	---	---	4.88	4.82	0	0.06	1.04	0.69	0.457
1556318_s_at	CAND1	cullin-associated and neddylation-dissociated 1	3.45	3.31	0	0.14	1.10	0.22	0.276
1556336_at	RBMX	RNA binding motif protein, X-linked	5.76	5.77	0	-0.01	-1.01	0.88	0.512
1556423_at	VASH1	vasohibin 1	3.25	3.33	0	-0.08	-1.06	0.56	0.424
1556462_a_at	---	---	3.45	3.53	0	-0.08	-1.06	0.29	0.313
1556471_at	SCML4	sex comb on midleg-like 4 (Drosophila)	5.03	5.04	0	-0.01	-1.01	0.83	0.499
1556621_a_at	---	---	4.34	4.25	0	0.09	1.06	0.29	0.315
1556750_at	LOC153577	uncharacterized LOC153577	3.68	3.74	0	-0.06	-1.04	0.58	0.434
1556812_a_at	---	---	4.76	4.78	0	-0.02	-1.01	0.81	0.492
1556931_at	CDC42-IT1	CDC42 intronic transcript 1	4.72	4.64	0	0.08	1.06	0.34	0.338
1557065_at	YLPM1	YLP motif containing 1	6.20	6.16	0	0.04	1.03	0.69	0.457
1557113_at	83588 /// RPS	uncharacterized LOC283588 /// ribosomal protein S6 kinase, 90kDa, polypeptide 5	4.11	4.07	0	0.04	1.03	0.65	0.450
1557145_at	STK38	serine/threonine kinase 38	4.42	4.27	0	0.15	1.11	0.34	0.338
1557227_s_at	TPR	translocated promoter region, nuclear basket protein	6.80	6.69	0	0.11	1.08	0.20	0.265
1557236_at	APOL6	apolipoprotein L, 6	5.25	5.25	0	0.00	1.00	0.97	0.535
1557238_s_at	---	---	5.32	5.26	0	0.06	1.04	0.65	0.450
1557267_s_at	GEN1	GEN1 Holliday junction 5' flap endonuclease	4.69	4.77	0	-0.08	-1.06	0.62	0.445
1557278_s_at	TNPO1	transportin 1	4.21	4.18	0	0.03	1.02	0.68	0.456
1557289_s_at	IRD2 /// GTF2	GTF2I repeat domain containing 2 /// GTF2I repeat domain containing 2B	5.27	5.11	0	0.16	1.12	0.16	0.236
1557293_at	LINC00969	long intergenic non-protein coding RNA 969	5.72	5.46	0	0.26	1.20	0.13	0.216
1557436_at	XKR6	X-linked Kx blood group related 6	3.92	3.92	0	0.00	1.00	0.98	0.538
1557512_at	---	---	3.40	3.39	0	0.01	1.01	0.88	0.515
1557519_at	---	---	3.18	3.19	0	-0.01	-1.01	0.85	0.502
1557527_at	---	---	5.19	4.98	0	0.22	1.16	0.16	0.236
1557529_at	HECTD4	HECT domain containing E3 ubiquitin protein ligase 4	5.30	5.28	0	0.02	1.02	0.74	0.468
1557538_at	---	---	4.51	4.20	0	0.31	1.24	0.13	0.214
1557562_at	---	---	3.19	3.00	0	0.19	1.14	0.17	0.243
1557626_at	---	---	4.35	4.26	0	0.09	1.06	0.58	0.432
1557828_a_at	C5orf28	chromosome 5 open reading frame 28	6.00	5.77	0	0.24	1.18	0.12	0.204
1557910_at	HSP90AB1	heat shock protein 90kDa alpha (cytosolic), class B member 1	11.15	11.19	0	-0.03	-1.02	0.62	0.445
1557987_at	---	---	7.16	6.85	0	0.31	1.24	0.12	0.202
1558078_at	---	---	5.77	5.45	0	0.32	1.25	0.14	0.224
1558093_s_at	MATR3	matrin 3	8.06	8.14	0	-0.08	-1.06	0.19	0.256
1558105_a_at	SLC9A7	solute carrier family 9, subfamily A (NHE7, cation proton antiporter 7), member 7	5.14	5.14	0	0.00	1.00	0.97	0.535
1558111_at	MBNL1	muscleblind like splicing regulator 1	8.62	8.59	0	0.02	1.02	0.63	0.448
1558279_a_at	KDSR	3-ketodihydrosphingosine reductase	4.05	4.03	0	0.03	1.02	0.76	0.477
1558569_at	.OC10013154:	uncharacterized LOC100131541	7.39	7.13	0	0.26	1.20	0.18	0.252
1558579_at	:105371215 //	uncharacterized LOC642691 /// uncharacterized LOC105371215 /// uncharacterized LOC105379432	3.78	3.66	0	0.12	1.09	0.30	0.317
1558641_at	SPATA24	spermatogenesis associated 24	7.31	7.32	0	-0.01	-1.01	0.74	0.469
1558678_s_at	MALAT1	metastasis associated lung adenocarcinoma transcript 1 (non-protein coding)	12.67	12.66	0	0.02	1.01	0.65	0.450
1558691_a_at	DOCK4	dedicator of cytokinesis 4	4.40	4.49	0	-0.09	-1.06	0.63	0.449
1558702_at	TEX10	testis expressed 10	3.96	3.95	0	0.01	1.01	0.81	0.492
1558719_s_at	RPAIN	RPA interacting protein	3.27	3.10	0	0.17	1.12	0.14	0.219
1558747_at	SMCHD1	structural maintenance of chromosomes flexible hinge domain containing 1	8.88	8.86	0	0.02	1.01	0.79	0.484
1558922_at	---	---	5.59	5.49	0	0.11	1.08	0.53	0.415
1558971_at	THEMIS	thymocyte selection associated	5.56	5.56	0	0.00	1.00	1.00	0.544
1559006_at	---	---	3.51	3.52	0	-0.01	-1.01	0.85	0.502
1559025_at	SEPT9	septin 9	5.26	5.03	0	0.24	1.18	0.16	0.234
1559045_at	.OC10012828!	uncharacterized LOC100128288	2.03	2.04	0	-0.01	-1.01	0.82	0.496
1559161_at	---	---	3.70	3.51	0	0.19	1.14	0.12	0.206
1559289_at	TADA1	transcriptional adaptor 1	3.09	3.03	0	0.06	1.05	0.33	0.333
1559362_at	---	---	5.97	5.76	0	0.21	1.16	0.15	0.231
1559437_at	---	---	4.33	4.28	0	0.05	1.03	0.64	0.450
1559776_at	---	---	5.98	6.01	0	-0.03	-1.02	0.58	0.434
1559822_s_at	MTDH	metadherin	6.70	6.74	0	-0.04	-1.02	0.67	0.454
1559848_at	NSUN4	NOP2/Sun domain family, member 4	2.35	2.28	0	0.07	1.05	0.16	0.234
1559932_at	---	---	4.08	4.09	0	-0.01	-1.01	0.92	0.526
1559954_s_at	DDX42	DEAD (Asp-Glu-Ala-Asp) box helicase 42	6.42	6.39	0	0.03	1.02	0.86	0.507
1559987_at	MSI2	musashi RNA binding protein 2	3.30	3.22	0	0.08	1.06	0.41	0.368
1560144_at	---	---	4.48	4.28	0	0.20	1.15	0.27	0.303
1560172_at	INTS10	integrator complex subunit 10	2.92	2.83	0	0.09	1.06	0.25	0.292
1560177_at	CCDC66	coiled-coil domain containing 66	2.47	2.45	0	0.02	1.01	0.72	0.467
1560386_at	---	---	3.49	3.42	0	0.07	1.05	0.51	0.409
1560556_a_at	PLEKHA8	pleckstrin homology domain containing, family A (phosphoinositide binding specific) member 8	2.59	2.56	0	0.03	1.02	0.58	0.434
1560676_at	SIAH3	siah E3 ubiquitin protein ligase family member 3	2.55	2.48	0	0.07	1.05	0.32	0.329
1560696_x_at	QTRTD1	queuine tRNA-ribosyltransferase domain containing 1	2.27	2.26	0	0.02	1.01	0.69	0.456
1560762_at	LINC00996	long intergenic non-protein coding RNA 996	5.54	5.38	0	0.15	1.11	0.27	0.302
1560776_at	---	---	2.62	2.56	0	0.06	1.04	0.18	0.252
1560779_a_at	ZNF568	zinc finger protein 568	2.31	2.27	0	0.05	1.03	0.27	0.302
1560869_a_at	---	---	5.55	5.51	0	0.04	1.03	0.73	0.468
1561654_at	---	---	3.48	3.36	0	0.11	1.08	0.23	0.288
1561660_at	SLC25A53	solute carrier family 25, member 53	3.24	3.26	0	-0.02	-1.01	0.82	0.493
1561754_at	---	---	6.44	6.29	0	0.16	1.11	0.30	0.318
1561973_at	SMARCC2	SWI/SNF related, matrix associated, actin dependent regulator of chromatin, subfamily c, member 2	4.53	4.48	0	0.06	1.04	0.70	0.457

Mean42 LRS	Mean 47 ROT1D	Mean 30 HRS	Mean 44 uHC	LR024	HR014	HR026	LR001	LR022	LR013	HR015	LR020	LR018	HR027	HR005	LR028	LR016	LR012	HR019	HR017	HR021	LR030	HR010	LR006	LR008	LR011	LR004	LR023	LR002

Probeset ID	Gene Symbol	Gene Title	Mean log2 intensity for 21 POST	Mean log2 intensity for 21 PRE	422 FDR <0.2 1=YES 0=NO	Log2 Ratio 21POST vs21PRE	Fold-Change 21POST vs21PRE	p value	FDR
1562031_at	JAK2	Janus kinase 2	4.72	4.60	0	0.12	1.09	0.17	0.247
1562230_at	---	---	2.86	2.88	0	-0.02	-1.01	0.76	0.474
1562250_at	---	---	3.59	3.41	0	0.19	1.14	0.25	0.292
1562307_at	---	---	2.32	2.27	0	0.04	1.03	0.44	0.377
1562316_at	---	---	2.34	2.26	0	0.07	1.05	0.18	0.255
1562364_at	GVINP1	GTPase, very large interferon inducible pseudogene 1	4.74	4.62	0	0.12	1.09	0.36	0.343
1562442_a_at	SSBP1	single-stranded DNA binding protein 1, mitochondrial	5.16	5.22	0	-0.06	-1.04	0.38	0.354
1562481_at	---	---	2.46	2.45	0	0.01	1.01	0.92	0.526
1562527_at	LOC441666	zinc finger protein 91 pseudogene	2.92	2.88	0	0.04	1.03	0.71	0.463
1562836_at	DDX6	DEAD (Asp-Glu-Ala-Asp) box helicase 6	5.62	5.73	0	-0.11	-1.08	0.27	0.304
1563173_at	---	---	2.24	2.21	0	0.03	1.02	0.58	0.434
1563431_x_at	PTGIR	prostaglandin I2 (prostacyclin) receptor (IP)	4.66	4.74	0	-0.08	-1.05	0.23	0.280
1563445_x_at	CTSLP8	cathepsin L pseudogene 8	5.35	5.40	0	-0.04	-1.03	0.62	0.445
1563460_at	---	---	2.15	2.12	0	0.03	1.02	0.46	0.383
1563724_at	---	---	2.23	2.16	0	0.07	1.05	0.14	0.224
1564002_a_at	AK9	adenylate kinase 9	5.82	5.88	0	-0.06	-1.04	0.19	0.256
1564053_a_at	YTHDF3	YTH N(6)-methyladenosine RNA binding protein 3	7.07	7.09	0	-0.02	-1.01	0.75	0.472
1564424_at	.OC10537975	uncharacterized LOC105379752	5.27	5.18	0	0.09	1.06	0.46	0.384
1564789_at	---	---	2.85	2.82	0	0.03	1.02	0.61	0.445
1565436_s_at	KMT2A	lysine (K)-specific methyltransferase 2A	6.51	6.52	0	-0.01	-1.01	0.93	0.526
1565598_at	---	---	5.24	5.33	0	-0.09	-1.06	0.48	0.395
1565810_at	---	---	2.42	2.36	0	0.06	1.04	0.24	0.292
1565875_at	---	---	2.45	2.48	0	-0.02	-1.02	0.60	0.441
1565886_at	---	---	6.85	6.84	0	0.01	1.00	0.94	0.528
1565887_at	---	---	4.29	4.38	0	-0.09	-1.06	0.38	0.355
1565913_at	---	---	5.29	5.23	0	0.06	1.05	0.47	0.392
1566040_at	.OC10028865	ankyrin repeat domain 20 family, member A2 pseudogene	3.19	3.14	0	0.05	1.03	0.31	0.324
1566603_s_at	RPUSD3	RNA pseudouridylate synthase domain containing 3	3.06	2.97	0	0.09	1.06	0.41	0.364
1566825_at	---	---	5.57	5.49	0	0.08	1.05	0.62	0.445
1566966_at	---	---	3.07	3.03	0	0.04	1.02	0.69	0.457
1566980_at	---	---	4.17	4.11	0	0.05	1.04	0.51	0.408
1568865_at	FNTB	farnesyltransferase, CAAX box, beta	3.22	3.12	0	0.10	1.07	0.31	0.325
1569128_at	C3orf38	chromosome 3 open reading frame 38(C3orf38)	2.14	2.06	0	0.08	1.06	0.18	0.254
1569129_s_at	C3orf38	chromosome 3 open reading frame 38(C3orf38)	5.28	5.21	0	0.07	1.05	0.59	0.438
1569142_at	TRIM13	tripartite motif containing 13	4.68	4.54	0	0.13	1.10	0.35	0.342
1569189_at	TTC9C	tetratricopeptide repeat domain 9C	4.13	3.98	0	0.15	1.11	0.23	0.285
1569311_at	---	---	2.93	2.86	0	0.08	1.05	0.17	0.243
1569320_at	GPBP1L1	GC-rich promoter binding protein 1-like 1	5.74	5.57	0	0.17	1.12	0.30	0.319
1569352_at	FNIP2	folliculin interacting protein 2	6.56	6.53	0	0.03	1.02	0.77	0.481
1569578_at	---	---	4.52	4.39	0	0.13	1.09	0.24	0.290
1569583_at	EREG	epiregulin	5.69	5.78	0	-0.09	-1.06	0.48	0.399
1569706_at	MYSM1	Myb-like, SWIRM and MPN domains 1	2.41	2.38	0	0.03	1.02	0.61	0.443
1569773_at	ATP8A1	ATPase, aminophospholipid transporter (APLT), class I, type 8A, member 1	3.67	3.64	0	0.02	1.01	0.81	0.492
1569791_at	STK4	serine/threonine kinase 4	5.70	5.75	0	-0.05	-1.03	0.55	0.423
1569830_at	PTPRC	protein tyrosine phosphatase, receptor type, C	5.08	5.16	0	-0.08	-1.06	0.45	0.381
1569864_at	SERAC1	serine active site containing 1	2.64	2.57	0	0.08	1.05	0.30	0.320
1569948_at	---	---	2.65	2.60	0	0.05	1.04	0.54	0.418
1570021_at	---	---	3.72	3.81	0	-0.09	-1.07	0.44	0.375
1570213_at	---	---	2.44	2.40	0	0.03	1.02	0.57	0.428
1570255_s_at	ANKRD20A3 /	ankyrin repeat domain 20 family, member A1 /// ankyrin repeat domain 20 family, member A12, pseudogene /// ank	3.50	3.32	0	0.18	1.13	0.20	0.263
1570338_at	---	---	2.97	2.93	0	0.03	1.02	0.54	0.419
1570364_at	ZNF709	zinc finger protein 709	2.54	2.58	0	-0.04	-1.03	0.62	0.445
1570371_a_at	EPT1	ethanolaminephosphotransferase 1	3.45	3.47	0	-0.02	-1.01	0.82	0.495
1570409_x_at	---	---	2.86	2.83	0	0.03	1.02	0.74	0.469
1570507_at	SCAF11	SR-related CTD-associated factor 11	5.42	5.28	0	0.13	1.10	0.20	0.268
200607_s_at	RAD21	RAD21 cohesin complex component	8.60	8.62	0	-0.03	-1.02	0.44	0.375
200671_s_at	SPTBN1	spectrin, beta, non-erythrocytic 1	4.90	4.95	0	-0.05	-1.03	0.30	0.319
200806_s_at	HSPD1	heat shock 60kDa protein 1 (chaperonin)	10.39	10.36	0	0.02	1.02	0.33	0.333
200832_s_at	SCD	stearoyl-CoA desaturase (delta-9-desaturase)	5.35	5.37	0	-0.02	-1.01	0.74	0.471
200928_s_at	RAB14	RAB14, member RAS oncogene family	2.97	2.88	0	0.09	1.07	0.22	0.276
200951_s_at	CCND2	cyclin D2	6.28	6.27	0	0.01	1.01	0.83	0.499
201008_s_at	TXNIP	thioredoxin interacting protein	10.22	10.17	0	0.05	1.03	0.69	0.457
201043_s_at	ANP32A	acidic nuclear phosphoprotein 32 family member A	6.30	6.32	0	-0.03	-1.02	0.86	0.507
201065_s_at	/ GTF2IP1 ///	general transcription factor IIi /// general transcription factor IIi pseudogene 1 /// general transcription factor IIi, pse	8.36	8.31	0	0.05	1.03	0.40	0.363
201070_x_at	SF3B1	splicing factor 3b, subunit 1, 155kDa	9.44	9.46	0	-0.02	-1.01	0.67	0.454
201073_s_at	SMARCC1	SWI/SNF related, matrix associated, actin dependent regulator of chromatin, subfamily c, member 1	5.94	5.91	0	0.03	1.02	0.66	0.452
201083_s_at	BCLAF1	BCL2-associated transcription factor 1	8.07	8.07	0	0.00	1.00	0.99	0.540
201085_s_at	SON	SON DNA binding protein	9.59	9.56	0	0.02	1.02	0.43	0.375
201299_s_at	MOB1A	MOB kinase activator 1A	8.76	8.80	0	-0.05	-1.03	0.48	0.395
201324_at	EMP1	epithelial membrane protein 1	10.11	10.19	0	-0.07	-1.05	0.12	0.204
201325_s_at	EMP1	epithelial membrane protein 1	8.49	8.58	0	-0.09	-1.07	0.17	0.247
201340_s_at	ENC1	ectodermal-neural cortex 1 (with BTB domain)	5.77	5.87	0	-0.10	-1.07	0.12	0.205
201446_s_at	TIA1	TIA1 cytotoxic granule-associated RNA binding protein	8.77	8.73	0	0.03	1.02	0.54	0.419
201450_s_at	TIA1	TIA1 cytotoxic granule-associated RNA binding protein	8.31	8.21	0	0.09	1.07	0.24	0.290
201490_s_at	PPIF	peptidylprolyl isomerase F	10.05	10.00	0	0.04	1.03	0.70	0.457
201631_s_at	IER3	immediate early response 3	11.47	11.53	0	-0.06	-1.04	0.47	0.390
201635_s_at	FXR1	fragile X mental retardation, autosomal homolog 1	8.54	8.53	0	0.01	1.00	0.92	0.526
201668_x_at	MARCKS	myristoylated alanine-rich protein kinase C substrate	7.33	7.33	0	0.00	1.00	0.99	0.542
201694_s_at	EGR1	early growth response 1	7.95	7.98	0	-0.02	-1.02	0.78	0.483
201711_x_at	RANBP2	RAN binding protein 2	10.18	10.21	0	-0.03	-1.02	0.54	0.418
201732_s_at	CLCN3	chloride channel, voltage-sensitive 3	5.23	5.32	0	-0.10	-1.07	0.18	0.256
201743_at	CD14	CD14 molecule	7.14	7.40	0	-0.26	-1.20	0.13	0.216
201868_s_at	TBL1X	transducin (beta)-like 1X-linked	5.39	5.38	0	0.01	1.00	0.95	0.532
201879_at	ARIH1	ariadne RBR E3 ubiquitin protein ligase 1	6.86	6.88	0	-0.02	-1.02	0.63	0.449
202118_s_at	CPNE3	copine III	7.07	7.07	0	0.00	1.00	0.98	0.539
202195_s_at	TMED5	transmembrane p24 trafficking protein 5	7.28	7.33	0	-0.05	-1.04	0.40	0.361
202241_at	TRIB1	tribbles pseudokinase 1	8.02	8.05	0	-0.03	-1.02	0.65	0.450
202388_at	RGS2	regulator of G-protein signaling 2	10.83	10.88	0	-0.05	-1.03	0.31	0.325
202412_s_at	USP1	ubiquitin specific peptidase 1	7.42	7.47	0	-0.05	-1.03	0.31	0.323
202430_s_at	PLSCR1	phospholipid scramblase 1	7.90	7.88	0	0.02	1.01	0.79	0.488
202581_at	PA1A /// HSPA	heat shock 70kDa protein 1A /// heat shock 70kDa protein 1B	7.58	7.54	0	0.04	1.03	0.65	0.450
202601_s_at	HTATSF1	HIV-1 Tat specific factor 1	5.07	5.17	0	-0.10	-1.07	0.26	0.300
202609_at	EPS8	epidermal growth factor receptor pathway substrate 8	7.29	7.28	0	0.00	1.00	0.96	0.535
202759_s_at	2 /// PALM2-A	A kinase (PRKA) anchor protein 2 /// PALM2-AKAP2 readthrough	7.83	7.86	0	-0.03	-1.02	0.52	0.412
202760_s_at	2 /// PALM2-A	A kinase (PRKA) anchor protein 2 /// PALM2-AKAP2 readthrough	8.12	8.13	0	-0.01	-1.00	0.93	0.526
202814_s_at	HEXIM1	hexamethylene bis-acetamide inducible 1	8.03	7.95	0	0.08	1.06	0.30	0.317
202855_s_at	SLC16A3	solute carrier family 16 (monocarboxylate transporter), member 3	6.68	6.72	0	-0.04	-1.03	0.84	0.499
202856_s_at	SLC16A3	solute carrier family 16 (monocarboxylate transporter), member 3	7.76	7.84	0	-0.08	-1.05	0.49	0.401
202869_at	OAS1	2'-5'-oligoadenylate synthetase 1	8.44	8.45	0	-0.01	-1.01	0.85	0.502
202876_s_at	PBX2	pre-B-cell leukemia homeobox 2	6.47	6.64	0	-0.16	-1.12	0.18	0.252
202887_s_at	DDIT4	DNA damage inducible transcript 4	9.93	9.91	0	0.03	1.02	0.79	0.484
202888_s_at	ANPEP	alanyl (membrane) aminopeptidase	8.40	8.51	0	-0.11	-1.08	0.43	0.374
203231_s_at	ATXN1	ataxin 1	5.50	5.51	0	-0.02	-1.01	0.84	0.501
203394_s_at	HES1	hes family bHLH transcription factor 1	8.24	8.20	0	0.05	1.03	0.54	0.417
203607_at	INPP5F	inositol polyphosphate-5-phosphatase F	6.63	6.59	0	0.03	1.02	0.68	0.456
203887_s_at	THBD	thrombomodulin	5.76	5.88	0	-0.13	-1.09	0.36	0.345
203888_at	THBD	thrombomodulin	4.87	4.98	0	-0.11	-1.08	0.37	0.350
203889_at	SCG5	secretogranin V	5.82	5.92	0	-0.11	-1.08	0.45	0.379
203958_s_at	ZBTB40	zinc finger and BTB domain containing 40	6.53	6.57	0	-0.04	-1.03	0.67	0.454
204011_at	SPRY2	sprouty RTK signaling antagonist 2	5.78	5.92	0	-0.14	-1.10	0.17	0.241
204014_at	DUSP4	dual specificity phosphatase 4	8.66	8.62	0	0.04	1.03	0.60	0.441
204015_s_at	DUSP4	dual specificity phosphatase 4	8.39	8.42	0	-0.03	-1.02	0.41	0.365
204088_at	P2RX4	purinergic receptor P2X, ligand gated ion channel, 4	8.66	8.63	0	0.04	1.03	0.78	0.482
204103_at	CCL4	chemokine (C-C motif) ligand 4	11.96	12.08	0	-0.13	-1.09	0.22	0.279
204165_at	WASF1	WAS protein family, member 1	3.77	3.74	0	0.02	1.01	0.70	0.457
204180_s_at	ZBTB43	zinc finger and BTB domain containing 43	6.91	6.95	0	-0.03	-1.02	0.62	0.445
204192_at	CD37	CD37 molecule	7.33	7.09	0	0.24	1.18	0.22	0.278
204270_at	SKI	SKI proto-oncogene	7.63	7.53	0	0.10	1.07	0.39	0.356
204470_at	CXCL1	chemokine (C-X-C motif) ligand 1 (melanoma growth stimulating activity, alpha)	11.49	11.61	0	-0.11	-1.08	0.39	0.356
204475_at	MMP1	matrix metallopeptidase 1	7.81	7.80	0	0.02	1.01	0.92	0.526
204560_at	FKBP5	FK506 binding protein 5	5.47	5.43	0	0.04	1.03	0.83	0.498
204628_s_at	ITGB3	integrin beta 3	4.83	4.80	0	0.03	1.02	0.69	0.457
204686_at	IRS1	insulin receptor substrate 1	3.51	3.44	0	0.07	1.05	0.41	0.366
204748_at	PTGS2	prostaglandin-endoperoxide synthase 2 (prostaglandin G/H synthase and cyclooxygenase)	9.87	9.95	0	-0.07	-1.05	0.67	0.454
204794_at	DUSP2	dual specificity phosphatase 2	8.84	8.88	0	-0.05	-1.03	0.16	0.240
204858_s_at	TYMP	thymidine phosphorylase	7.13	7.05	0	0.08	1.06	0.63	0.448
204863_s_at	IL6ST	interleukin 6 signal transducer	7.13	7.24	0	-0.11	-1.08	0.20	0.265
204866_at	JADE3	jade family PHD finger 3	5.96	5.93	0	0.03	1.02	0.70	0.458
204956_at	MTAP	methylthioadenosine phosphorylase	2.66	2.73	0	-0.07	-1.05	0.35	0.342
204995_at	CDK5R1	cyclin-dependent kinase 5, regulatory subunit 1 (p35)	5.23	5.12	0	0.11	1.08	0.24	0.292
205010_at	GNL3L	guanine nucleotide binding protein-like 3 (nucleolar)-like	6.08	5.95	0	0.13	1.09	0.25	0.292
205114_s_at	// CCL3L1 /// (	chemokine (C-C motif) ligand 3 /// chemokine (C-C motif) ligand 3-like 1 /// chemokine (C-C motif) ligand 3-like 3	12.12	12.15	0	-0.04	-1.03	0.67	0.454
205119_s_at	FPR1	formyl peptide receptor 1	6.55	6.62	0	-0.07	-1.05	0.36	0.344
205174_s_at	QPCT	glutaminyl-peptide cyclotransferase	5.05	4.97	0	0.08	1.06	0.37	0.351
205197_s_at	ATP7A	ATPase, Cu++ transporting, alpha polypeptide	6.64	6.66	0	-0.01	-1.01	0.90	0.519
205238_at	TRMT2B	tRNA methyltransferase 2 homolog B	4.40	4.17	0	0.23	1.18	0.16	0.233
205285_s_at	FYB	FYN binding protein	7.69	7.59	0	0.10	1.07	0.39	0.357
205411_at	STK4	serine/threonine kinase 4	5.30	5.34	0	-0.04	-1.03	0.64	0.449
205476_at	CCL20	chemokine (C-C motif) ligand 20	11.01	11.03	0	-0.03	-1.02	0.82	0.494
205495_s_at	GNLY	granulysin	10.53	10.48	0	0.05	1.03	0.51	0.409
205596_s_at	SMURF2	SMAD specific E3 ubiquitin protein ligase 2	8.05	8.04	0	0.01	1.01	0.73	0.468
205684_s_at	DENND4C	DENN/MADD domain containing 4C	6.12	6.02	0	0.10	1.07	0.27	0.302
205822_s_at	HMGCS1	3-hydroxy-3-methylglutaryl-CoA synthase 1 (soluble)	5.66	5.70	0	-0.04	-1.03	0.56	0.424
205885_s_at	ITGA4	integrin alpha 4	6.60	6.61	0	-0.01	-1.01	0.81	0.492
205934_at	PLCL1	phospholipase C-like 1	3.92	3.93	0	-0.01	-1.01	0.92	0.526
206157_at	PTX3	pentraxin 3, long	5.75	5.83	0	-0.08	-1.05	0.33	0.333
206180_x_at	ZNF747	zinc finger protein 747	5.93	5.95	0	-0.02	-1.02	0.71	0.459
206241_at	KPNA5	karyopherin alpha 5 (importin alpha 6)	5.54	5.61	0	-0.07	-1.05	0.28	0.304

Mean42 LRS	Mean 47 ROT1D	Mean 30 HRS	Mean 44 uHC	LR024	HR014	HR026	LR001	LR022	LR013	HR015	LR020	LR018	HR027	HR005	LR028	LR016	LR012	HR019	HR017	HR021	LR030	HR010	LR006	LR008	LR011	LR004	LR023	LR002

Probeset ID	Gene Symbol	Gene Title	Mean log2 intensity for 21 POST	Mean log2 intensity for 21 PRE	422 FDR <0.2 1=YES 0=NO	Log2 Ratio 21POST vs21PRE	Fold-Change 21POST vs21PRE	p value	FDR
206279_at	PRKY	protein kinase, Y-linked, pseudogene	5.28	5.19	0	0.08	1.06	0.46	0.384
206341_at	IL2RA	interleukin 2 receptor, alpha	6.42	6.38	0	0.04	1.03	0.65	0.450
206366_x_at	XCL1	chemokine (C motif) ligand 1	7.33	7.36	0	-0.03	-1.02	0.61	0.441
206500_s_at	MIS18BP1	MIS18 binding protein 1	7.13	7.23	0	-0.10	-1.07	0.13	0.214
206548_at	---	---	4.39	4.30	0	0.09	1.06	0.63	0.448
206652_at	ZMYM5	zinc finger, MYM-type 5	6.96	6.98	0	-0.03	-1.02	0.59	0.435
206667_s_at	SCAMP1	secretory carrier membrane protein 1	5.35	5.38	0	-0.02	-1.02	0.72	0.466
206928_at	ZNF124	zinc finger protein 124	4.60	4.55	0	0.06	1.04	0.49	0.400
207038_at	SLC16A6	solute carrier family 16, member 6	5.69	5.78	0	-0.08	-1.06	0.32	0.330
207219_at	ZFP69B	ZFP69 zinc finger protein B	3.46	3.40	0	0.06	1.04	0.50	0.402
207237_at	KCNA3	potassium channel, voltage gated shaker related subfamily A, member 3	6.00	5.94	0	0.05	1.04	0.60	0.440
207316_at	HAS1	hyaluronan synthase 1	3.05	3.06	0	-0.01	-1.01	0.87	0.508
207334_s_at	TGFBR2	transforming growth factor beta receptor II	5.78	5.76	0	0.02	1.01	0.87	0.508
207388_s_at	PTGES	prostaglandin E synthase	5.14	5.17	0	-0.02	-1.02	0.74	0.469
207533_at	CCL1	chemokine (C-C motif) ligand 1	5.26	5.34	0	-0.08	-1.06	0.48	0.398
207545_s_at	)1928143 /// I	uncharacterized LOC101928143 /// numb homolog (Drosophila)	6.99	7.11	0	-0.11	-1.08	0.21	0.271
207549_x_at	CD46	CD46 molecule, complement regulatory protein	7.58	7.55	0	0.03	1.02	0.52	0.412
207630_s_at	CREM	cAMP responsive element modulator	10.96	10.99	0	-0.04	-1.03	0.36	0.344
207674_at	FCAR	Fc fragment of IgA receptor	6.32	6.44	0	-0.12	-1.09	0.50	0.402
207786_at	CYP2R1	cytochrome P450, family 2, subfamily R, polypeptide 1	4.31	4.26	0	0.04	1.03	0.61	0.441
207819_s_at	ABCB4	ATP binding cassette subfamily B member 4	5.02	4.95	0	0.07	1.05	0.21	0.269
207850_at	CXCL3	chemokine (C-X-C motif) ligand 3	11.07	11.15	0	-0.08	-1.06	0.47	0.391
207852_at	CXCL5	chemokine (C-X-C motif) ligand 5	4.64	4.66	0	-0.01	-1.01	0.91	0.524
207907_at	TNFSF14	tumor necrosis factor (ligand) superfamily, member 14	6.01	6.01	0	-0.01	-1.01	0.95	0.532
208067_x_at	UTY	ubiquitously transcribed tetratricopeptide repeat containing, Y-linked	7.26	7.35	0	-0.08	-1.06	0.24	0.290
208075_s_at	CCL7	chemokine (C-C motif) ligand 7	6.51	6.54	0	-0.04	-1.03	0.81	0.492
208178_x_at	TRIO	trio Rho guanine nucleotide exchange factor	5.51	5.53	0	-0.02	-1.01	0.78	0.484
208198_x_at	105379650 //,	killer cell immunoglobulin-like receptor, two domains, short cytoplasmic tail, 1 /// killer cell immunoglobulin-like rece	4.02	4.03	0	-0.01	-1.01	0.80	0.492
208601_s_at	TUBB1	tubulin, beta 1 class VI	6.44	6.50	0	-0.06	-1.04	0.51	0.408
208621_s_at	EZR	ezrin	9.77	9.76	0	0.01	1.01	0.85	0.504
208664_s_at	TTC3	tetratricopeptide repeat domain 3	6.54	6.55	0	0.00	-1.00	0.96	0.534
208797_s_at	GOLGA8A	golgin A8 family, member A	6.76	6.65	0	0.11	1.08	0.32	0.328
208930_s_at	ILF3	interleukin enhancer binding factor 3	7.76	7.77	0	-0.01	-1.01	0.89	0.516
208965_s_at	IFI16	interferon, gamma-inducible protein 16	10.54	10.50	0	0.04	1.03	0.36	0.343
209013_x_at	TRIO	trio Rho guanine nucleotide exchange factor	5.54	5.53	0	0.02	1.01	0.86	0.507
209060_x_at	NCOA3	nuclear receptor coactivator 3	7.40	7.43	0	-0.03	-1.02	0.36	0.343
209203_s_at	BICD2	bicaudal D homolog 2 (Drosophila)	6.25	6.21	0	0.04	1.03	0.65	0.450
209277_at	TFPI2	tissue factor pathway inhibitor 2	8.28	8.31	0	-0.03	-1.02	0.83	0.498
209321_s_at	ADCY3	adenylate cyclase 3	5.63	5.52	0	0.11	1.08	0.38	0.354
209446_s_at	---	---	5.92	5.87	0	0.05	1.03	0.74	0.469
209599_s_at	PRUNE	prune exopolyphosphatase	3.76	3.77	0	0.00	-1.00	0.95	0.531
209616_s_at	ES1 /// CES1P	carboxylesterase 1 /// carboxylesterase 1 pseudogene 1	7.04	7.09	0	-0.05	-1.03	0.63	0.449
209675_s_at	HNRNPUL1	heterogeneous nuclear ribonucleoprotein U-like 1	6.20	6.10	0	0.10	1.07	0.24	0.292
209699_x_at	AKR1C2	aldo-keto reductase family 1, member C2	6.51	6.62	0	-0.11	-1.08	0.14	0.219
209715_at	CBX5	chromobox homolog 5	5.27	5.30	0	-0.03	-1.02	0.57	0.428
209750_at	NR1D2	nuclear receptor subfamily 1, group D, member 2	7.79	7.83	0	-0.04	-1.03	0.36	0.344
209774_x_at	CXCL2	chemokine (C-X-C motif) ligand 2	12.80	12.85	0	-0.04	-1.03	0.53	0.416
209790_s_at	CASP6	caspase 6	5.83	5.79	0	0.04	1.03	0.42	0.372
209803_s_at	PHLDA2	pleckstrin homology-like domain, family A, member 2	6.82	6.88	0	-0.06	-1.04	0.44	0.377
209865_at	SLC35A3	solute carrier family 35 (UDP-N-acetylglucosamine (UDP-GlcNAc) transporter), member A3	3.74	3.73	0	0.00	1.00	0.99	0.540
209933_s_at	CD300A	CD300a molecule	7.65	7.65	0	-0.01	-1.01	0.88	0.512
209967_s_at	CREM	cAMP responsive element modulator	10.79	10.84	0	-0.05	-1.03	0.25	0.294
210057_at	SMG1	SMG1 phosphatidylinositol 3-kinase-related kinase	4.60	4.51	0	0.10	1.07	0.35	0.342
210118_s_at	IL1A	interleukin 1 alpha	10.03	10.11	0	-0.08	-1.06	0.55	0.422
210119_at	KCNJ15	potassium channel, inwardly rectifying subfamily J, member 15	3.91	3.95	0	-0.04	-1.03	0.59	0.438
210140_at	CST7	cystatin F (leukocystatin)	8.48	8.50	0	-0.02	-1.01	0.73	0.468
210218_s_at	SP100	SP100 nuclear antigen	8.23	8.27	0	-0.04	-1.03	0.44	0.377
210251_s_at	RUFY3	RUN and FYVE domain containing 3	5.98	5.95	0	0.03	1.02	0.70	0.457
210282_at	ZMYM2	zinc finger, MYM-type 2	6.48	6.36	0	0.12	1.09	0.29	0.315
210367_s_at	PTGES	prostaglandin E synthase	3.49	3.53	0	-0.04	-1.03	0.56	0.427
210436_at	CCT8	chaperonin containing TCP1, subunit 8 (theta)	3.09	3.09	0	0.01	1.01	0.92	0.526
210693_at	SPPL2B	signal peptide peptidase like 2B	5.26	5.24	0	0.02	1.01	0.84	0.499
210717_at	---	---	3.22	3.32	0	-0.10	-1.07	0.46	0.384
210772_at	FPR2	formyl peptide receptor 2	4.96	5.04	0	-0.09	-1.06	0.35	0.339
210773_s_at	FPR2	formyl peptide receptor 2	4.79	4.91	0	-0.12	-1.09	0.19	0.256
210807_s_at	SLC16A7	solute carrier family 16 (monocarboxylate transporter), member 7	4.24	4.14	0	0.09	1.07	0.32	0.329
210818_s_at	:H1 /// GRIK1-	BTB and CNC homology 1, basic leucine zipper transcription factor 1 /// GRIK1 antisense RNA 2	6.74	6.84	0	-0.10	-1.07	0.39	0.357
210867_at	CNOT4	CCR4-NOT transcription complex subunit 4	3.68	3.63	0	0.05	1.04	0.50	0.402
210942_s_at	ST3GAL6	ST3 beta-galactoside alpha-2,3-sialyltransferase 6	6.90	7.01	0	-0.11	-1.08	0.16	0.234
210965_x_at	CDK13	cyclin-dependent kinase 13	5.23	5.08	0	0.16	1.11	0.12	0.208
210985_s_at	SP100	SP100 nuclear antigen	4.42	4.29	0	0.13	1.09	0.22	0.278
211022_s_at	ATRX	alpha thalassemia/mental retardation syndrome X-linked	5.75	5.82	0	-0.07	-1.05	0.47	0.391
211074_at	---	---	8.39	8.35	0	0.04	1.03	0.62	0.445
211081_s_at	MAP4K5	mitogen-activated protein kinase kinase kinase kinase 5	6.06	6.04	0	0.02	1.02	0.72	0.465
211192_s_at	CD84	CD84 molecule	5.37	5.40	0	-0.03	-1.02	0.70	0.457
211307_s_at	FCAR	Fc fragment of IgA receptor	6.55	6.57	0	-0.03	-1.02	0.90	0.521
211310_at	EZH1	enhancer of zeste 1 polycomb repressive complex 2 subunit	4.57	4.64	0	-0.07	-1.05	0.65	0.450
211429_s_at	SERPINA1	serpin peptidase inhibitor, clade A (alpha-1 antiproteinase, antitrypsin), member 1	8.58	8.63	0	-0.05	-1.04	0.38	0.354
211538_s_at	HSPA2	heat shock 70kDa protein 2	4.49	4.49	0	0.00	1.00	0.97	0.536
211688_x_at	3DL1 /// KIR3I	killer cell immunoglobulin-like receptor, three domains, long cytoplasmic tail, 1 /// killer cell immunoglobulin-like rec	4.38	4.41	0	-0.04	-1.03	0.55	0.422
211721_s_at	ZNF551	zinc finger protein 551	5.46	5.31	0	0.15	1.11	0.20	0.262
211751_at	4 /// LOC6535	phosphodiesterase 4D interacting protein-like /// phosphodiesterase 4D interacting protein-like /// phosphodiestera	4.33	4.27	0	0.06	1.04	0.51	0.409
211794_at	FYB	FYN binding protein	6.08	6.12	0	-0.03	-1.02	0.52	0.412
211816_x_at	FCAR	Fc fragment of IgA receptor	6.71	6.92	0	-0.21	-1.16	0.22	0.279
211821_x_at	GYPA	glycophorin A (MNS blood group)	3.31	3.31	0	0.01	1.00	0.91	0.524
211899_s_at	TRAF4	TNF receptor-associated factor 4	5.22	5.27	0	-0.05	-1.03	0.73	0.468
211947_s_at	PRRC2C	proline-rich coiled-coil 2C	6.83	6.86	0	-0.03	-1.02	0.64	0.449
211965_at	ZFP36L1	ZFP36 ring finger protein-like 1	6.63	6.65	0	-0.02	-1.01	0.81	0.492
211973_at	NUDT3	nudix hydrolase 3	5.50	5.56	0	-0.06	-1.04	0.50	0.402
212079_s_at	KMT2A	lysine (K)-specific methyltransferase 2A	7.00	7.00	0	0.00	1.00	0.97	0.536
212105_s_at	DHX9	DEAH (Asp-Glu-Ala-His) box helicase 9	6.06	6.10	0	-0.04	-1.03	0.75	0.474
212107_s_at	DHX9	DEAH (Asp-Glu-Ala-His) box helicase 9	7.94	8.00	0	-0.06	-1.04	0.20	0.263
212172_at	AK2	adenylate kinase 2	3.83	3.78	0	0.05	1.03	0.53	0.415
212182_at	NUDT4	nudix hydrolase 4	2.46	2.37	0	0.08	1.06	0.12	0.202
212249_at	PIK3R1	phosphoinositide-3-kinase, regulatory subunit 1 (alpha)	7.16	7.18	0	-0.03	-1.02	0.78	0.483
212286_at	ANKRD12	ankyrin repeat domain 12	8.99	9.01	0	-0.02	-1.01	0.64	0.449
212332_at	RBL2	retinoblastoma-like 2	7.23	7.19	0	0.03	1.02	0.64	0.449
212392_s_at	)996724 /// PI	phosphodiesterase 4D interacting protein-like /// phosphodiesterase 4D interacting protein	5.31	5.37	0	-0.06	-1.04	0.70	0.457
212417_at	SCAMP1	secretory carrier membrane protein 1	6.86	6.88	0	-0.02	-1.02	0.59	0.438
212451_at	SECISBP2L	SECIS binding protein 2-like	7.13	7.21	0	-0.08	-1.06	0.18	0.256
212592_at	JCHAIN	joining chain of multimeric IgA and IgM	8.50	8.44	0	0.06	1.04	0.36	0.345
212650_at	EHBP1	EH domain binding protein 1	5.83	5.91	0	-0.08	-1.06	0.24	0.291
212720_at	PAPOLA	poly(A) polymerase alpha	6.50	6.47	0	0.03	1.02	0.65	0.450
212758_s_at	ZEB1	zinc finger E-box binding homeobox 1	7.96	7.88	0	0.08	1.06	0.13	0.209
212762_s_at	TCF7L2	transcription factor 7-like 2 (T-cell specific, HMG-box)	5.46	5.49	0	-0.04	-1.03	0.73	0.468
212979_s_at	TCAF1	TRPM8 channel-associated factor 1	5.18	5.06	0	0.12	1.09	0.17	0.243
213005_s_at	KANK1	KN motif and ankyrin repeat domains 1	6.53	6.60	0	-0.07	-1.05	0.49	0.402
213286_at	ZFR	zinc finger RNA binding protein	6.75	6.85	0	-0.10	-1.07	0.19	0.256
213355_at	ST3GAL6	ST3 beta-galactoside alpha-2,3-sialyltransferase 6	7.08	7.15	0	-0.07	-1.05	0.37	0.350
213430_at	RUFY3	RUN and FYVE domain containing 3	3.69	3.71	0	-0.02	-1.02	0.81	0.492
213436_at	CNR1	cannabinoid receptor 1 (brain)	4.33	4.19	0	0.13	1.10	0.13	0.216
213457_at	MFHAS1	malignant fibrous histiocytoma amplified sequence 1	6.79	6.83	0	-0.04	-1.03	0.66	0.450
213472_at	HNRNPH1	heterogeneous nuclear ribonucleoprotein H1 (H)	5.22	5.15	0	0.08	1.06	0.67	0.454
213566_at	RNASE6	ribonuclease, RNase A family, k6	6.43	6.40	0	0.03	1.02	0.75	0.474
213638_at	PHACTR1	phosphatase and actin regulator 1	8.02	7.99	0	0.03	1.02	0.66	0.452
213875_x_at	C6orf62	chromosome 6 open reading frame 62	7.77	7.83	0	-0.05	-1.04	0.55	0.422
213956_at	CEP350	centrosomal protein 350kDa	6.66	6.72	0	-0.06	-1.04	0.52	0.412
213971_s_at	Z12 /// SUZ12	SUZ12 polycomb repressive complex 2 subunit /// SUZ12 polycomb repressive complex 2 subunit pseudogene 1	7.51	7.50	0	0.01	1.01	0.84	0.499
214034_at	ERAP1	endoplasmic reticulum aminopeptidase 1	4.61	4.71	0	-0.09	-1.07	0.15	0.228
214041_x_at	RPL37A	ribosomal protein L37a	5.68	5.73	0	-0.05	-1.03	0.78	0.484
214149_s_at	ATP6V0E1	ATPase, H+ transporting, lysosomal 9kDa, V0 subunit e1	5.97	6.06	0	-0.09	-1.06	0.60	0.439
214183_s_at	TKTL1	transketolase-like 1	5.16	5.29	0	-0.13	-1.09	0.32	0.329
214184_at	NPFF	neuropeptide FF-amide peptide precursor	3.97	3.96	0	0.01	1.01	0.93	0.527
214250_at	NUMA1	nuclear mitotic apparatus protein 1	6.59	6.45	0	0.14	1.10	0.27	0.304
214251_s_at	NUMA1	nuclear mitotic apparatus protein 1	4.66	4.59	0	0.07	1.05	0.46	0.384
214305_s_at	SF3B1	splicing factor 3b, subunit 1, 155kDa	9.83	9.88	0	-0.05	-1.03	0.26	0.300
214352_s_at	KRAS	Kirsten rat sarcoma viral oncogene homolog	7.21	7.21	0	0.00	-1.00	0.99	0.542
214483_s_at	ARFIP1	ADP-ribosylation factor interacting protein 1	5.91	5.94	0	-0.03	-1.02	0.53	0.412
214499_s_at	BCLAF1	BCL2-associated transcription factor 1	8.94	8.91	0	0.03	1.02	0.55	0.422
214508_x_at	CREM	cAMP responsive element modulator	9.99	10.03	0	-0.04	-1.03	0.39	0.356
214567_s_at	XCL1 /// XCL2	chemokine (C motif) ligand 1 /// chemokine (C motif) ligand 2	7.04	7.06	0	-0.02	-1.01	0.77	0.481
214615_at	P2RY10	purinergic receptor P2Y, G-protein coupled, 10	6.88	6.81	0	0.06	1.04	0.25	0.293
214724_at	DIXDC1	DIX domain containing 1	2.91	2.87	0	0.04	1.03	0.43	0.374
214786_at	MAP3K1	mitogen-activated protein kinase kinase kinase 1, E3 ubiquitin protein ligase	7.32	7.29	0	0.02	1.02	0.82	0.495
214806_at	BICD1	bicaudal D homolog 1 (Drosophila)	4.27	4.34	0	-0.07	-1.05	0.44	0.376
214815_at	TRIM33	tripartite motif containing 33	5.67	5.56	0	0.11	1.08	0.21	0.275
214833_at	TMEM63A	transmembrane protein 63A	5.31	5.25	0	0.06	1.04	0.61	0.441
214834_at	PWAR5	Prader Willi/Angelman region RNA 5	2.79	2.81	0	-0.03	-1.02	0.64	0.449
214843_s_at	USP33	ubiquitin specific peptidase 33	7.75	7.77	0	-0.02	-1.01	0.72	0.465
214881_s_at	UBTF	upstream binding transcription factor, RNA polymerase I	4.22	4.18	0	0.04	1.03	0.66	0.451
214918_at	HNRNPM	heterogeneous nuclear ribonucleoprotein M	3.81	3.78	0	0.04	1.03	0.76	0.475
214974_x_at	CXCL5	chemokine (C-X-C motif) ligand 5	11.51	11.51	0	0.00	1.00	1.00	0.544
214982_at	SNRNP200	small nuclear ribonucleoprotein, U5 200kDa subunit	2.78	2.67	0	0.11	1.08	0.28	0.304
215043_s_at	100653061 ///	glucuronidase, beta pseudogene 3 /// beta-glucuronidase-like protein SMA4 /// glucuronidase, beta pseudogene ///	4.80	4.65	0	0.15	1.11	0.17	0.243
215049_x_at	CD163	CD163 molecule	7.53	7.67	0	-0.13	-1.10	0.15	0.232
215092_s_at	NFAT5	nuclear factor of activated T-cells 5, tonicity-responsive	6.53	6.58	0	-0.05	-1.04	0.50	0.405
215101_s_at	CXCL5	chemokine (C-X-C motif) ligand 5	10.76	10.77	0	-0.01	-1.01	0.94	0.528
215137_at	---	---	5.79	5.65	0	0.15	1.11	0.19	0.256

Mean42 LRS	Mean 47 ROT1D	Mean 30 HRS	Mean 44 uHC	LR024	HR014	HR026	LR001	LR022	LR013	HR015	LR020	LR018	HR027	HR005	LR028	LR016	LR012	HR019	HR017	HR021	LR030	HR010	LR006	LR008	LR011	LR004	LR023	LR002

Probeset ID	Gene Symbol	Gene Title	Mean log2 intensity for 21 POST	Mean log2 intensity for 21 PRE	422 FDR <0.2 1=YES 0=NO	Log2 Ratio 21POST vs21PRE	Fold-Change 21POST vs21PRE	p value	FDR
215188_at	STK24	serine/threonine kinase 24	5.37	5.22	0	0.15	1.11	0.21	0.269
215191_at	---	---	4.96	4.75	0	0.21	1.16	0.20	0.265
215220_s_at	TPR	translocated promoter region, nuclear basket protein	5.70	5.59	0	0.11	1.08	0.38	0.351
215284_at	---	---	4.86	4.86	0	0.01	1.00	0.96	0.535
215308_at	XRCC6	X-ray repair complementing defective repair in Chinese hamster cells 6	2.56	2.52	0	0.03	1.02	0.52	0.412
215318_at	MINOS1P1	mitochondrial inner membrane organizing system 1 pseudogene 1	5.05	4.80	0	0.25	1.19	0.12	0.206
215330_at	---	---	6.61	6.39	0	0.22	1.17	0.19	0.256
215339_at	NKTR	natural killer cell triggering receptor	4.75	4.74	0	0.01	1.01	0.92	0.526
215342_s_at	RABGAP1L	RAB GTPase activating protein 1-like	6.33	6.27	0	0.06	1.04	0.33	0.333
215352_at	GIMAP5	GTPase, IMAP family member 5	4.82	4.89	0	-0.07	-1.05	0.55	0.422
215434_x_at	/ NBPF1 /// N	neuroblastoma breakpoint family member 1 /// neuroblastoma breakpoint family, member 1 /// neuroblastoma bre	9.46	9.53	0	-0.07	-1.05	0.39	0.357
215555_at	---	---	4.88	4.65	0	0.23	1.17	0.23	0.283
215586_at	PPP3CB	---	3.32	3.17	0	0.15	1.11	0.21	0.271
215589_at	SMURF1	---	3.62	3.68	0	-0.06	-1.04	0.42	0.371
215698_at	KDM5A	lysine (K)-specific demethylase 5A	6.16	6.19	0	-0.03	-1.02	0.64	0.450
215761_at	DMXL2	Dmx-like 2	5.17	5.11	0	0.06	1.04	0.74	0.469
215866_at	---	---	3.32	3.21	0	0.11	1.08	0.14	0.225
215889_at	SKIL	SKI-like proto-oncogene	4.44	4.43	0	0.02	1.01	0.86	0.506
215986_at	---	---	2.57	2.45	0	0.11	1.08	0.22	0.276
215991_s_at	EMC1	ER membrane protein complex subunit 1	2.80	2.76	0	0.04	1.03	0.50	0.402
216015_s_at	NLRP3	NLR family, pyrin domain containing 3	4.79	4.97	0	-0.18	-1.13	0.24	0.291
216016_at	NLRP3	NLR family, pyrin domain containing 3	2.83	2.73	0	0.09	1.07	0.21	0.275
216069_at	PRMT2	protein arginine methyltransferase 2	6.73	6.69	0	0.04	1.03	0.73	0.468
216101_at	---	---	4.65	4.64	0	0.00	1.00	0.99	0.540
216117_at	EXOSC2	exosome component 2	2.78	2.76	0	0.02	1.02	0.60	0.440
216248_s_at	NR4A2	nuclear receptor subfamily 4, group A, member 2	10.09	10.12	0	-0.03	-1.02	0.33	0.333
216361_s_at	KAT6A	K(lysine) acetyltransferase 6A	5.20	5.22	0	-0.02	-1.02	0.88	0.515
216370_s_at	TKTL1	transketolase-like 1	4.38	4.50	0	-0.11	-1.08	0.39	0.356
216433_s_at	PRDM2	PR domain containing 2, with ZNF domain	3.61	3.66	0	-0.05	-1.04	0.65	0.450
216594_x_at	AKR1C1	aldo-keto reductase family 1, member C1	6.37	6.45	0	-0.08	-1.06	0.16	0.234
216595_at	FAM186A	family with sequence similarity 186, member A	3.16	3.02	0	0.14	1.10	0.16	0.234
216683_at	---	---	3.00	2.93	0	0.07	1.05	0.42	0.369
216901_s_at	IKZF1	IKAROS family zinc finger 1	5.98	5.84	0	0.14	1.11	0.42	0.372
217234_s_at	EZR	ezrin	10.23	10.27	0	-0.05	-1.03	0.39	0.357
217507_at	SLC11A1	solute carrier family 11 (proton-coupled divalent metal ion transporter), member 1	3.17	3.15	0	0.03	1.02	0.74	0.469
217529_at	ORAI2	ORAI calcium release-activated calcium modulator 2	6.46	6.28	0	0.18	1.13	0.21	0.275
217540_at	NXPE3	neurexophilin and PC-esterase domain family, member 3	3.85	3.92	0	-0.07	-1.05	0.26	0.301
217576_x_at	SOS2	SOS Ras/Rho guanine nucleotide exchange factor 2	5.74	5.72	0	0.02	1.01	0.76	0.477
217602_at	PPIA	peptidylprolyl isomerase A (cyclophilin A)	3.84	3.81	0	0.03	1.02	0.84	0.500
217644_s_at	SOS2	SOS Ras/Rho guanine nucleotide exchange factor 2	5.03	4.91	0	0.13	1.09	0.33	0.332
217656_at	---	---	5.35	5.29	0	0.06	1.04	0.59	0.438
217813_s_at	SPIN1	spindlin 1	4.99	5.13	0	-0.14	-1.10	0.27	0.304
217878_s_at	CDC27	cell division cycle 27	7.66	7.68	0	-0.02	-1.02	0.66	0.451
217951_s_at	PHF3	PHD finger protein 3	6.83	6.87	0	-0.03	-1.02	0.65	0.450
218166_s_at	RSF1	remodeling and spacing factor 1	6.51	6.57	0	-0.06	-1.04	0.33	0.334
218404_at	SNX10	sorting nexin 10	9.05	9.09	0	-0.04	-1.03	0.44	0.375
218430_s_at	RFX7	regulatory factor X, 7	5.06	4.96	0	0.10	1.07	0.37	0.349
218469_at	GREM1	gremlin 1, DAN family BMP antagonist	4.96	5.07	0	-0.10	-1.07	0.41	0.365
218748_s_at	EXOC5	exocyst complex component 5	5.64	5.71	0	-0.08	-1.06	0.34	0.337
218839_at	HEY1	hes-related family bHLH transcription factor with YRPW motif 1	6.46	6.59	0	-0.12	-1.09	0.19	0.256
218930_s_at	TMEM106B	transmembrane protein 106B	5.65	5.68	0	-0.02	-1.02	0.68	0.456
218967_s_at	PTER	phosphotriesterase related	5.72	5.73	0	0.00	-1.00	0.96	0.533
219403_s_at	HPSE	heparanase	6.64	6.69	0	-0.05	-1.04	0.59	0.438
219518_s_at	ELL3	elongation factor RNA polymerase II-like 3	5.75	5.73	0	0.02	1.01	0.73	0.468
219574_at	MARCH1	membrane associated ring finger 1	6.92	6.88	0	0.04	1.03	0.62	0.445
219730_at	MED18	mediator complex subunit 18	2.40	2.31	0	0.08	1.06	0.24	0.292
219844_at	CCDC186	coiled-coil domain containing 186	5.39	5.45	0	-0.06	-1.04	0.43	0.374
219858_s_at	MFSD6	major facilitator superfamily domain containing 6	4.99	5.02	0	-0.02	-1.02	0.84	0.501
219864_s_at	RCAN3	RCAN family member 3	4.28	4.27	0	0.01	1.00	0.95	0.533
219922_s_at	LTBP3	latent transforming growth factor beta binding protein 3	5.86	5.67	0	0.19	1.14	0.39	0.357
219969_at	TXLNG	taxilin gamma	4.76	4.75	0	0.02	1.01	0.83	0.499
220038_at	f44-SGK3 /// S	C8orf44-SGK3 readthrough /// serum/glucocorticoid regulated kinase family, member 3	4.85	4.91	0	-0.06	-1.04	0.60	0.441
220068_at	VPREB3	pre-B lymphocyte 3	5.67	5.69	0	-0.02	-1.01	0.87	0.507
220220_at	371804 /// LRF	leucine-rich repeat-containing protein 37A3 pseudogene /// leucine rich repeat containing 37, member A4, pseudog	3.61	3.57	0	0.04	1.03	0.68	0.455
220253_s_at	LRP12	LDL receptor related protein 12	5.50	5.52	0	-0.01	-1.01	0.89	0.518
220586_at	CHD9	chromodomain helicase DNA binding protein 9	2.54	2.43	0	0.11	1.08	0.13	0.215
220643_s_at	FAIM	Fas apoptotic inhibitory molecule	5.44	5.45	0	-0.01	-1.01	0.82	0.495
220704_at	IKZF1	IKAROS family zinc finger 1	5.51	5.28	0	0.23	1.17	0.22	0.276
221092_at	IKZF3	IKAROS family zinc finger 3	5.16	4.99	0	0.17	1.12	0.45	0.379
221231_s_at	NRDE2	NRDE-2, necessary for RNA interference, domain containing	2.51	2.49	0	0.03	1.02	0.65	0.450
221345_at	FFAR2	free fatty acid receptor 2	5.08	5.15	0	-0.07	-1.05	0.56	0.424
221496_s_at	TOB2	transducer of ERBB2, 2	5.82	5.87	0	-0.06	-1.04	0.50	0.406
221695_s_at	MAP3K2	mitogen-activated protein kinase kinase kinase 2	7.13	7.15	0	-0.02	-1.02	0.75	0.473
221705_s_at	SIKE1	suppressor of IKBKE 1	5.79	5.72	0	0.06	1.04	0.42	0.369
221986_s_at	KLHL24	kelch-like family member 24	7.31	7.27	0	0.04	1.03	0.44	0.376
222313_at	---	---	7.39	7.39	0	0.00	1.00	0.99	0.540
222343_at	BCL2L11	BCL2-like 11 (apoptosis facilitator)	5.88	5.85	0	0.04	1.03	0.64	0.449
222387_s_at	VPS35	VPS35 retromer complex component	7.59	7.66	0	-0.07	-1.05	0.17	0.247
222406_s_at	PNRC2	proline-rich nuclear receptor coactivator 2	9.01	8.96	0	0.05	1.04	0.38	0.354
222473_s_at	ERBB2IP	erbb2 interacting protein	8.00	8.00	0	0.01	1.00	0.89	0.516
222508_s_at	ARGLU1	arginine and glutamate rich 1	7.53	7.45	0	0.07	1.05	0.57	0.429
222527_s_at	RBM22	RNA binding motif protein 22	7.49	7.55	0	-0.06	-1.04	0.51	0.409
222538_s_at	APPL1	adaptor protein, phosphotyrosine interaction, PH domain and leucine zipper containing 1	6.65	6.62	0	0.03	1.02	0.69	0.457
222562_s_at	TNKS2	tankyrase, TRF1-interacting ankyrin-related ADP-ribose polymerase 2	8.37	8.44	0	-0.07	-1.05	0.22	0.278
222600_s_at	UBA6	ubiquitin-like modifier activating enzyme 6	7.23	7.31	0	-0.08	-1.06	0.15	0.231
222634_s_at	TBL1XR1	transducin (beta)-like 1 X-linked receptor 1	5.90	5.90	0	-0.01	-1.00	0.89	0.518
222670_s_at	MAFB	v-maf avian musculoaponeurotic fibrosarcoma oncogene homolog B	9.28	9.32	0	-0.04	-1.03	0.49	0.401
222679_s_at	DCUN1D1	DCN1, defective in cullin neddylation 1, domain containing 1	6.74	6.77	0	-0.04	-1.03	0.43	0.374
222714_s_at	LACTB2	lactamase, beta 2	5.24	5.22	0	0.02	1.02	0.80	0.489
222880_at	AKT3	v-akt murine thymoma viral oncogene homolog 3	4.60	4.62	0	-0.02	-1.01	0.89	0.517
222906_at	FLVCR1	feline leukemia virus subgroup C cellular receptor 1	5.16	5.02	0	0.15	1.11	0.22	0.277
223038_s_at	FAM60A	family with sequence similarity 60, member A	7.95	7.99	0	-0.03	-1.02	0.62	0.445
223079_s_at	GLS	glutaminase	7.79	7.81	0	-0.02	-1.01	0.73	0.468
223254_s_at	G2E3	G2/M-phase specific E3 ubiquitin protein ligase	6.20	6.20	0	0.00	-1.00	0.94	0.528
223314_at	TSPAN14	tetraspanin 14	6.56	6.49	0	0.07	1.05	0.31	0.324
223465_at	COL4A3BP	collagen, type IV, alpha 3 (Goodpasture antigen) binding protein	6.98	7.00	0	-0.02	-1.01	0.73	0.468
223578_x_at	MALAT1	metastasis associated lung adenocarcinoma transcript 1 (non-protein coding)(MALAT1)	5.72	5.75	0	-0.03	-1.02	0.87	0.507
223681_s_at	INADL	InaD-like (Drosophila)	3.76	3.72	0	0.04	1.03	0.67	0.454
223701_s_at	USP47	ubiquitin specific peptidase 47	8.46	8.45	0	0.01	1.01	0.80	0.489
223771_at	TMEM87A	transmembrane protein 87A	3.18	3.14	0	0.04	1.03	0.67	0.454
223814_at	TRNT1	tRNA nucleotidyl transferase, CCA-adding, 1	4.07	3.98	0	0.09	1.07	0.34	0.338
223890_at	PRO1082	uncharacterized protein PRO1082	3.06	3.10	0	-0.04	-1.03	0.57	0.428
223940_x_at	MALAT1	metastasis associated lung adenocarcinoma transcript 1 (non-protein coding)	9.99	9.80	0	0.19	1.14	0.27	0.302
223984_s_at	NUP58	nucleoporin 58kDa	4.44	4.38	0	0.06	1.04	0.64	0.450
224012_at	20A2 /// ANKF	ankyrin repeat domain 20 family, member A1 /// ankyrin repeat domain 20 family, member A2 /// ankyrin repeat do	2.36	2.29	0	0.06	1.05	0.36	0.345
224098_at	---	---	3.91	3.79	0	0.13	1.09	0.15	0.227
224152_s_at	PBRM1	polybromo 1	4.40	4.46	0	-0.06	-1.04	0.50	0.402
224227_s_at	BDP1	B double prime 1, subunit of RNA polymerase III transcription initiation factor IIIB	5.90	5.93	0	-0.03	-1.02	0.72	0.467
224310_s_at	BCL11B	B-cell CLL/lymphoma 11B (zinc finger protein)	3.82	3.97	0	-0.16	-1.12	0.29	0.312
224311_s_at	CAB39	calcium binding protein 39	6.71	6.76	0	-0.06	-1.04	0.33	0.333
224435_at	FAM213A	family with sequence similarity 213, member A	3.66	3.60	0	0.07	1.05	0.54	0.421
224568_x_at	MALAT1	metastasis associated lung adenocarcinoma transcript 1 (non-protein coding)	9.84	9.64	0	0.20	1.15	0.17	0.243
224580_at	SLC38A1	solute carrier family 38, member 1	6.63	6.71	0	-0.09	-1.06	0.25	0.293
224618_at	PTBP3	polypyrimidine tract binding protein 3	7.12	7.03	0	0.09	1.06	0.22	0.276
224775_at	IWS1	IWS1 homolog (S. cerevisiae)	7.16	7.21	0	-0.06	-1.04	0.15	0.228
224875_at	C5orf24	chromosome 5 open reading frame 24	5.80	5.70	0	0.10	1.07	0.44	0.376
224940_s_at	PAPPA	pregnancy-associated plasma protein A, pappalysin 1	2.94	2.81	0	0.12	1.09	0.36	0.345
224989_at	SMIM14	small integral membrane protein 14	6.95	7.01	0	-0.06	-1.04	0.55	0.423
224996_at	ASPH	aspartate beta-hydroxylase	6.35	6.47	0	-0.13	-1.09	0.17	0.247
225046_at	724441 /// LO	uncharacterized LOC102723360 /// uncharacterized LOC102724219 /// uncharacterized LOC102724441 /// uncharac	4.65	4.55	0	0.10	1.07	0.32	0.331
225237_s_at	MSI2	musashi RNA binding protein 2	5.18	5.21	0	-0.03	-1.02	0.79	0.484
225662_at	ZAK	sterile alpha motif and leucine zipper containing kinase AZK	7.04	7.13	0	-0.09	-1.06	0.37	0.349
225746_at	RAB11FIP4	RAB11 family interacting protein 4 (class II)	3.14	3.08	0	0.05	1.04	0.61	0.441
225800_at	JAZF1	JAZF zinc finger 1	7.03	7.08	0	-0.05	-1.03	0.32	0.329
225955_at	METRNL	meteorin, glial cell differentiation regulator-like	8.14	8.18	0	-0.03	-1.02	0.47	0.390
226034_at	DUSP4	dual specificity phosphatase 4	7.78	7.78	0	0.01	1.00	0.92	0.526
226184_at	FMNL2	formin like 2	4.93	5.02	0	-0.09	-1.06	0.41	0.368
226367_at	KDM5A	lysine (K)-specific demethylase 5A	4.29	4.38	0	-0.09	-1.06	0.25	0.294
226404_at	RBM39	RNA binding motif protein 39	7.15	7.17	0	-0.02	-1.01	0.81	0.492
226550_at	SLC9A7	solute carrier family 9, subfamily A (NHE7, cation proton antiporter 7), member 7	6.56	6.57	0	-0.02	-1.01	0.81	0.492
226663_at	)10 /// ANKRD	ankyrin repeat domain 10 /// ANKRD10 intronic transcript 1	7.89	7.69	0	0.20	1.15	0.20	0.265
226694_at	2 /// PALM2-A	A kinase (PRKA) anchor protein 2 /// PALM2-AKAP2 readthrough	8.74	8.72	0	0.02	1.01	0.67	0.454
226975_at	RNPC3	RNA binding region (RNP1, RRM) containing 3	7.41	7.44	0	-0.03	-1.02	0.58	0.433
227062_at	NEAT1	nuclear paraspeckle assembly transcript 1 (non-protein coding)	7.44	7.43	0	0.01	1.01	0.92	0.526
227101_at	ZNF800	zinc finger protein 800	8.34	8.42	0	-0.08	-1.06	0.13	0.216
227168_at	MIAT	myocardial infarction associated transcript (non-protein coding)	7.93	7.79	0	0.13	1.10	0.24	0.292
227195_at	ZNF503	zinc finger protein 503	3.50	3.52	0	-0.02	-1.01	0.80	0.489
227259_at	CD47	CD47 molecule	6.68	6.70	0	-0.03	-1.02	0.74	0.469
227497_at	SOX6	SRY box 6	3.19	3.15	0	0.04	1.03	0.66	0.451
227510_x_at	MALAT1	metastasis associated lung adenocarcinoma transcript 1 (non-protein coding)(MALAT1)	5.95	5.86	0	0.09	1.06	0.64	0.450
227613_at	ZNF331	zinc finger protein 331	7.72	7.79	0	-0.07	-1.05	0.17	0.243
227719_at	SMAD9	SMAD family member 9	3.70	3.62	0	0.09	1.06	0.25	0.292
227740_at	UHMK1	U2AF homology motif (UHM) kinase 1	9.10	9.14	0	-0.04	-1.03	0.61	0.441
227777_at	---	---	5.73	5.71	0	0.02	1.02	0.75	0.473
227884_at	TAF15	TATA box binding protein associated factor 15	5.95	6.01	0	-0.06	-1.04	0.67	0.454
227891_s_at	TAF15	TATA box binding protein associated factor 15	6.99	6.80	0	0.19	1.14	0.25	0.292
227948_at	FGD4	FYVE, RhoGEF and PH domain containing 4	4.23	4.27	0	-0.04	-1.03	0.42	0.369
228155_at	FAM213A	family with sequence similarity 213, member A	4.63	4.68	0	-0.04	-1.03	0.50	0.402

Mean42 LRS	Mean 47 ROT1D	Mean 30 HRS	Mean 44 uHC	LR024	HR014	HR026	LR001	LR022	LR013	HR015	LR020	LR018	HR027	HR005	LR028	LR016	LR012	HR019	HR017	HR021	LR030	HR010	LR006	LR008	LR011	LR004	LR023	LR002

Probeset ID	Gene Symbol	Gene Title	Mean log2 intensity for 21 POST	Mean log2 intensity for 21 PRE	422 FDR <0.2 1=YES 0=NO	Log2 Ratio 21POST vs21PRE	Fold-Change 21POST vs21PRE	p value	FDR
228170_at	OLIG1	oligodendrocyte transcription factor 1	3.76	3.78	0	-0.03	-1.02	0.76	0.477
228188_at	FOSL2	FOS-like antigen 2	8.24	8.28	0	-0.05	-1.03	0.31	0.324
228250_at	FNIP1	folliculin interacting protein 1	6.49	6.52	0	-0.03	-1.02	0.69	0.457
228478_at	SPPL2A	signal peptide peptidase like 2A	7.20	7.17	0	0.03	1.02	0.69	0.457
228549_at	TMEM63A	transmembrane protein 63A	6.83	6.70	0	0.12	1.09	0.27	0.303
228654_at	SPIN4	spindlin family, member 4	4.74	4.80	0	-0.06	-1.04	0.25	0.292
228986_at	OSBPL8	oxysterol binding protein-like 8	8.22	8.28	0	-0.06	-1.05	0.41	0.365
228988_at	ZNF711	zinc finger protein 711	4.65	4.58	0	0.07	1.05	0.25	0.292
229075_at	SPATA5	spermatogenesis associated 5	2.68	2.77	0	-0.09	-1.07	0.17	0.243
229115_at	DYNC1H1	dynein, cytoplasmic 1, heavy chain 1	6.44	6.35	0	0.09	1.06	0.30	0.319
229274_at	GNAS	GNAS complex locus	7.25	7.24	0	0.01	1.01	0.86	0.506
229389_at	ATG16L2	autophagy related 16-like 2	6.38	6.42	0	-0.04	-1.03	0.69	0.457
229399_at	CCDC186	coiled-coil domain containing 186	6.99	7.07	0	-0.07	-1.05	0.36	0.344
229413_s_at	---	---	4.42	4.34	0	0.08	1.06	0.43	0.374
229437_at	MIR155HG	MIR155 host gene	11.40	11.38	0	0.02	1.01	0.68	0.455
229553_at	PGM2L1	phosphoglucomutase 2-like 1	7.01	7.01	0	0.00	-1.00	0.99	0.542
229706_at	TCERG1	transcription elongation regulator 1	3.37	3.47	0	-0.10	-1.07	0.15	0.231
229732_at	ZNF823	zinc finger protein 823	3.95	3.92	0	0.03	1.02	0.63	0.448
229841_at	---	---	5.91	6.00	0	-0.09	-1.06	0.28	0.305
229850_at	KDSR	3-ketodihydrosphingosine reductase	5.69	5.56	0	0.14	1.10	0.18	0.252
229869_at	---	---	4.61	4.59	0	0.02	1.01	0.78	0.483
229881_at	KLF12	Kruppel-like factor 12	5.71	5.71	0	-0.01	-1.00	0.96	0.533
229966_at	EWSR1	EWS RNA binding protein 1	6.68	6.64	0	0.04	1.03	0.75	0.472
230000_at	RNF213	ring finger protein 213	7.43	7.33	0	0.10	1.07	0.34	0.337
230014_at	---	---	4.84	4.89	0	-0.06	-1.04	0.44	0.377
230028_at	KIAA0907	KIAA0907	5.31	5.26	0	0.06	1.04	0.49	0.402
230106_at	ZXDC	ZXD family zinc finger C	5.69	5.73	0	-0.04	-1.03	0.61	0.441
230127_at	---	---	5.66	5.65	0	0.01	1.01	0.92	0.526
230180_at	DDX17	DEAD (Asp-Glu-Ala-Asp) box helicase 17	8.24	8.19	0	0.05	1.03	0.70	0.457
230323_s_at	TMEM45B	transmembrane protein 45B	4.14	4.22	0	-0.08	-1.05	0.57	0.428
230352_at	PRPS2	phosphoribosyl pyrophosphate synthetase 2	4.05	4.06	0	-0.01	-1.01	0.91	0.523
230434_at	PHOSPHO2	phosphatase, orphan 2	4.53	4.59	0	-0.05	-1.04	0.46	0.382
230511_at	CREM	cAMP responsive element modulator	8.45	8.48	0	-0.03	-1.02	0.42	0.368
230607_at	---	---	5.29	5.43	0	-0.14	-1.11	0.12	0.204
230681_at	TBRG1	transforming growth factor beta regulator 1	5.56	5.53	0	0.02	1.02	0.85	0.504
230742_at	RBM5	RNA binding motif protein 5	7.23	7.18	0	0.05	1.03	0.74	0.469
230820_at	SMURF2	SMAD specific E3 ubiquitin protein ligase 2	5.57	5.67	0	-0.11	-1.08	0.28	0.306
230896_at	BEND4	BEN domain containing 4	4.35	4.27	0	0.08	1.05	0.31	0.323
231108_at	FUS	FUS RNA binding protein	7.18	6.90	0	0.28	1.22	0.12	0.208
231109_at	---	---	6.31	6.24	0	0.07	1.05	0.51	0.409
231235_at	NKTR	natural killer cell triggering receptor	5.64	5.58	0	0.07	1.05	0.53	0.413
231370_at	PPM1A	protein phosphatase, Mg2+/Mn2+ dependent, 1A	4.98	5.02	0	-0.05	-1.03	0.58	0.433
231597_x_at	---	---	8.21	8.06	0	0.15	1.11	0.22	0.276
231644_at	---	---	3.30	3.20	0	0.10	1.07	0.27	0.303
231697_s_at	---	---	5.80	5.99	0	-0.19	-1.14	0.13	0.209
231829_at	MAVS	mitochondrial antiviral signaling protein	3.35	3.34	0	0.02	1.01	0.83	0.499
231862_at	CBX5	chromobox homolog 5	4.85	4.78	0	0.08	1.05	0.34	0.338
231873_at	BMPR2	bone morphogenetic protein receptor type II	5.80	5.82	0	-0.02	-1.01	0.88	0.514
231934_at	TRIM14	tripartite motif containing 14	4.59	4.62	0	-0.03	-1.02	0.66	0.452
231956_at	RNF213	ring finger protein 213	6.86	6.64	0	0.22	1.16	0.19	0.258
231995_at	CAAP1	caspase activity and apoptosis inhibitor 1	3.87	3.82	0	0.05	1.03	0.34	0.338
232000_at	TTC39B	tetratricopeptide repeat domain 39B	4.07	4.01	0	0.06	1.04	0.45	0.378
232020_at	SMURF2	SMAD specific E3 ubiquitin protein ligase 2	3.83	3.72	0	0.11	1.08	0.40	0.361
232179_at	---	---	3.92	3.88	0	0.04	1.03	0.74	0.469
232204_at	EBF1	early B-cell factor 1	7.12	7.12	0	0.00	-1.00	0.97	0.535
232319_at	---	---	3.87	3.77	0	0.09	1.07	0.31	0.323
232386_at	VPS13C	vacuolar protein sorting 13 homolog C (S. cerevisiae)	3.93	3.97	0	-0.04	-1.03	0.71	0.463
232466_at	CUL4A	cullin 4A	4.87	4.66	0	0.22	1.16	0.19	0.256
232472_at	---	---	4.66	4.56	0	0.10	1.07	0.54	0.419
232584_at	---	---	6.09	5.82	0	0.27	1.21	0.12	0.204
232614_at	---	---	6.79	6.58	0	0.21	1.15	0.26	0.297
232648_at	PSMA3	proteasome subunit alpha 3	4.20	4.20	0	0.00	1.00	0.95	0.532
232653_at	---	---	5.85	5.87	0	-0.02	-1.01	0.79	0.488
232688_at	BMP2K	BMP2 inducible kinase	3.19	3.11	0	0.08	1.06	0.25	0.292
232740_at	MCM3AP-AS1	MCM3AP antisense RNA 1	2.88	2.87	0	0.01	1.01	0.81	0.492
232757_at	MTSS1	---	4.33	4.25	0	0.07	1.05	0.68	0.456
232861_at	PDP2	pyruvate dehyrogenase phosphatase catalytic subunit 2	2.66	2.58	0	0.07	1.05	0.31	0.326
232879_at	CRTC3	CREB regulated transcription coactivator 3	4.27	3.98	0	0.30	1.23	0.14	0.219
232885_at	BBIP1	BBSome interacting protein 1	4.59	4.39	0	0.21	1.15	0.25	0.292
232896_at	ERBB2IP	erbb2 interacting protein	2.43	2.36	0	0.06	1.04	0.29	0.315
232980_at	LMBRD1	LMBR1 domain containing 1	2.80	2.82	0	-0.02	-1.02	0.68	0.455
233011_at	ANXA1	annexin A1	6.76	6.96	0	-0.20	-1.15	0.24	0.290
233121_at	---	---	5.08	4.89	0	0.19	1.14	0.25	0.294
233248_at	---	---	4.49	4.37	0	0.12	1.09	0.16	0.234
233251_at	STRBP	spermatid perinuclear RNA binding protein	4.71	4.64	0	0.07	1.05	0.56	0.424
233283_at	---	---	3.46	3.47	0	-0.01	-1.01	0.82	0.495
233309_at	---	---	5.80	5.72	0	0.08	1.06	0.33	0.333
233314_at	PTEN	---	4.00	4.09	0	-0.09	-1.06	0.58	0.434
233387_s_at	PCNT	pericentrin	4.16	4.10	0	0.05	1.04	0.44	0.377
233405_at	---	---	4.30	4.13	0	0.17	1.13	0.17	0.243
233419_at	---	---	2.16	2.14	0	0.02	1.02	0.63	0.449
233515_at	KRR1	KRR1, small subunit (SSU) processome component, homolog (yeast)	4.02	3.96	0	0.05	1.04	0.43	0.374
233543_s_at	FAM175A	family with sequence similarity 175, member A	4.77	4.75	0	0.02	1.02	0.77	0.479
233559_s_at	WDFY1	WD repeat and FYVE domain containing 1	8.34	8.41	0	-0.07	-1.05	0.14	0.219
233664_at	---	---	3.81	3.73	0	0.08	1.05	0.25	0.292
233724_at	ARNT	aryl hydrocarbon receptor nuclear translocator	3.33	3.26	0	0.07	1.05	0.32	0.329
233819_s_at	LTN1	listerin E3 ubiquitin protein ligase 1	6.34	6.42	0	-0.08	-1.06	0.40	0.362
233866_at	KLHL5	kelch-like family member 5	4.28	4.14	0	0.14	1.10	0.19	0.256
233957_at	---	---	2.88	2.93	0	-0.05	-1.04	0.51	0.408
234149_at	---	---	3.18	3.03	0	0.15	1.11	0.13	0.216
234150_at	---	---	4.64	4.64	0	0.01	1.00	0.94	0.528
234306_s_at	SLAMF7	SLAM family member 7	9.16	9.19	0	-0.03	-1.02	0.57	0.428
234632_x_at	---	---	3.50	3.30	0	0.20	1.15	0.17	0.243
234645_at	---	---	5.09	4.91	0	0.18	1.13	0.32	0.329
235003_at	UHMK1	U2AF homology motif (UHM) kinase 1	8.24	8.33	0	-0.08	-1.06	0.32	0.331
235008_at	---	---	6.18	6.19	0	-0.01	-1.01	0.95	0.532
235011_at	MAP3K2	mitogen-activated protein kinase kinase kinase 2	4.22	4.31	0	-0.09	-1.06	0.32	0.329
235052_at	ZNF792	zinc finger protein 792	4.38	4.43	0	-0.05	-1.03	0.43	0.374
235058_at	GPN2	GPN-loop GTPase 2	4.93	4.89	0	0.05	1.03	0.67	0.454
235060_at	.OC10019098(	uncharacterized LOC100190986	7.73	7.55	0	0.18	1.13	0.44	0.377
235172_at	GABPB1-AS1	GABPB1 antisense RNA 1(GABPB1-AS1)	3.99	3.91	0	0.08	1.06	0.49	0.400
235180_at	STYX	serine/threonine/tyrosine interacting protein	4.08	4.03	0	0.05	1.04	0.39	0.357
235220_at	YIPF4	Yip1 domain family member 4	3.10	2.99	0	0.11	1.08	0.15	0.229
235274_at	GABPB1-AS1	GABPB1 antisense RNA 1(GABPB1-AS1)	4.11	3.94	0	0.17	1.12	0.19	0.256
235430_at	ELMSAN1	ELM2 and Myb/SANT-like domain containing 1	4.80	4.80	0	0.00	1.00	0.97	0.536
235442_at	CXorf56	chromosome X open reading frame 56	2.83	2.64	0	0.20	1.15	0.16	0.234
235451_at	SMAD5	SMAD family member 5	5.90	5.95	0	-0.05	-1.04	0.46	0.384
235469_at	AM133DP ///	family with sequence similarity 133, member B /// family with sequence similarity 133, member A pseudogene /// fa	4.85	4.87	0	-0.02	-1.01	0.81	0.492
235476_at	TRIM59	tripartite motif containing 59	6.47	6.38	0	0.10	1.07	0.35	0.342
235537_at	OCIAD1	OCIA domain containing 1	5.60	5.61	0	-0.01	-1.01	0.91	0.524
235567_at	RORA	RAR-related orphan receptor A	6.44	6.48	0	-0.03	-1.02	0.63	0.448
235568_at	MCEMP1	mast cell-expressed membrane protein 1	5.10	5.17	0	-0.07	-1.05	0.50	0.402
235574_at	GBP4	guanylate binding protein 4	7.10	7.15	0	-0.05	-1.03	0.56	0.427
235577_at	ZNF652	zinc finger protein 652	4.25	4.31	0	-0.07	-1.05	0.45	0.380
235611_at	SREK1	splicing regulatory glutamine/lysine-rich protein 1	4.98	4.93	0	0.05	1.03	0.62	0.445
235643_at	SAMD9L	sterile alpha motif domain containing 9-like	9.20	9.11	0	0.09	1.06	0.35	0.339
235652_at	SCML1	sex comb on midleg-like 1 (Drosophila)	7.42	7.41	0	0.01	1.01	0.88	0.512
235653_s_at	THAP6	THAP domain containing 6	4.23	4.11	0	0.12	1.09	0.30	0.317
235743_at	---	---	6.77	6.73	0	0.04	1.03	0.80	0.489
235765_at	TLE4	transducin-like enhancer of split 4	6.46	6.52	0	-0.06	-1.05	0.33	0.333
235803_at	---	---	5.40	5.28	0	0.13	1.09	0.55	0.422
235811_at	---	---	7.16	6.98	0	0.18	1.13	0.21	0.269
236000_s_at	---	---	7.12	7.19	0	-0.07	-1.05	0.54	0.419
236005_at	---	---	5.04	4.96	0	0.08	1.06	0.27	0.302
236023_at	CDK9	cyclin-dependent kinase 9	5.04	5.08	0	-0.03	-1.02	0.78	0.483
236032_at	---	---	3.66	3.68	0	-0.03	-1.02	0.63	0.448
236125_at	---	---	6.02	6.05	0	-0.02	-1.02	0.70	0.458
236202_at	.OC10537445(	uncharacterized LOC105374450	4.10	4.01	0	0.09	1.06	0.30	0.320
236237_at	---	---	3.97	3.93	0	0.03	1.02	0.71	0.463
236327_at	CEPT1	choline/ethanolamine phosphotransferase 1(CEPT1)	2.99	3.05	0	-0.06	-1.04	0.49	0.400
236356_at	NDUFS1	NADH dehydrogenase (ubiquinone) Fe-S protein 1, 75kDa (NADH-coenzyme Q reductase)	3.82	3.97	0	-0.15	-1.11	0.26	0.299
236386_at	SUZ12P1	SUZ12 polycomb repressive complex 2 subunit pseudogene 1(SUZ12P1)	3.52	3.52	0	-0.01	-1.01	0.93	0.528
236417_at	---	---	6.62	6.59	0	0.03	1.02	0.70	0.457
236505_at	NUP62	nucleoporin 62kDa	3.44	3.45	0	-0.01	-1.01	0.94	0.528
236558_at	---	---	5.37	5.38	0	-0.01	-1.01	0.92	0.526
236645_at	HBP1	HMG-box transcription factor 1	3.52	3.57	0	-0.05	-1.03	0.57	0.428
236723_at	---	---	4.66	4.77	0	-0.11	-1.08	0.42	0.372
236876_at	H1FNT	H1 histone family, member N, testis-specific	3.48	3.46	0	0.02	1.01	0.77	0.479
236887_at	KIN	Kin17 DNA and RNA binding protein	3.30	3.25	0	0.05	1.03	0.51	0.409
236944_at	---	---	3.04	2.98	0	0.07	1.05	0.53	0.415
236946_at	---	---	4.04	3.99	0	0.05	1.04	0.55	0.423
236962_at	---	---	5.95	6.06	0	-0.11	-1.08	0.29	0.313
236971_at	---	---	3.24	3.17	0	0.07	1.05	0.37	0.349
237118_at	---	---	4.97	4.98	0	-0.01	-1.01	0.90	0.521
237184_at	---	---	4.82	4.74	0	0.07	1.05	0.37	0.351
237252_at	THBD	thrombomodulin	3.83	3.88	0	-0.05	-1.03	0.45	0.379
237318_at	---	---	4.23	4.33	0	-0.10	-1.07	0.33	0.333
237333_at	RBBP4	retinoblastoma binding protein 4	5.30	5.26	0	0.04	1.03	0.73	0.468

Mean42 LRS	Mean 47 ROT1D	Mean 30 HRS	Mean 44 uHC	LR024	HR014	HR026	LR001	LR022	LR013	HR015	LR020	LR018	HR027	HR005	LR028	LR016	LR012	HR019	HR017	HR021	LR030	HR010	LR006	LR008	LR011	LR004	LR023	LR002

Probeset ID	Gene Symbol	Gene Title	Mean log2 intensity for 21 POST	Mean log2 intensity for 21 PRE	422 FDR <0.2 1=YES 0=NO	Log2 Ratio 21POST vs21PRE	Fold-Change 21POST vs21PRE	p value	FDR
237376_at	---	---	4.23	4.28	0	-0.05	-1.04	0.70	0.457
237412_at	---	---	4.77	4.69	0	0.08	1.05	0.38	0.354
237502_at	CRLS1	cardiolipin synthase 1	3.47	3.46	0	0.01	1.00	0.95	0.532
237554_at	---	---	4.73	4.68	0	0.05	1.03	0.61	0.444
237577_at	PCNP	PEST proteolytic signal containing nuclear protein	4.22	4.06	0	0.17	1.12	0.12	0.204
237586_at	---	---	4.22	4.07	0	0.15	1.11	0.19	0.256
237588_at	---	---	4.29	4.37	0	-0.08	-1.06	0.37	0.349
237625_s_at	---	---	5.53	5.44	0	0.09	1.06	0.34	0.338
237689_at	---	---	4.49	4.38	0	0.12	1.09	0.43	0.374
237741_at	SLC25A36	solute carrier family 25 (pyrimidine nucleotide carrier), member 36	3.90	3.99	0	-0.10	-1.07	0.27	0.302
237747_at	---	---	6.60	6.48	0	0.12	1.09	0.28	0.305
237813_at	PCBP2	poly(rC) binding protein 2	4.58	4.63	0	-0.05	-1.03	0.34	0.338
237878_at	---	---	4.12	4.06	0	0.06	1.04	0.47	0.390
237890_at	.OC10537494	uncharacterized LOC105374947	4.68	4.75	0	-0.07	-1.05	0.35	0.342
238159_at	---	---	4.99	5.05	0	-0.06	-1.04	0.44	0.376
238220_at	KDM6A	lysine (K)-specific demethylase 6A	3.26	3.31	0	-0.05	-1.04	0.43	0.374
238281_at	---	---	3.52	3.60	0	-0.08	-1.06	0.28	0.304
238317_x_at	---	---	4.93	4.98	0	-0.05	-1.03	0.58	0.432
238342_at	---	---	4.99	4.88	0	0.12	1.08	0.26	0.300
238350_at	UBN2	ubinuclein 2	5.15	5.23	0	-0.08	-1.06	0.35	0.342
238356_at	DOCK11	dedicator of cytokinesis 11	6.19	6.12	0	0.07	1.05	0.42	0.369
238525_at	DDX56	DEAD (Asp-Glu-Ala-Asp) box helicase 56	4.45	4.45	0	0.00	-1.00	0.96	0.533
238563_at	---	---	6.59	6.66	0	-0.07	-1.05	0.57	0.428
238644_at	MYSM1	Myb-like, SWIRM and MPN domains 1	4.79	4.79	0	0.00	-1.00	0.96	0.534
238706_at	PAPD4	PAP associated domain containing 4	6.38	6.35	0	0.03	1.02	0.70	0.458
238785_at	---	---	3.99	3.98	0	0.01	1.00	0.92	0.526
238831_at	TMEM33	transmembrane protein 33	4.60	4.59	0	0.01	1.01	0.93	0.526
238848_at	OTUD4	OTU deubiquitinase 4	4.47	4.52	0	-0.05	-1.04	0.40	0.362
238884_at	---	---	4.66	4.66	0	0.00	1.00	0.98	0.540
238899_at	---	---	3.02	2.91	0	0.12	1.08	0.21	0.274
238913_at	---	---	6.57	6.44	0	0.13	1.09	0.11	0.201
238940_at	KLF12	Kruppel-like factor 12	3.06	3.07	0	-0.01	-1.01	0.80	0.490
238954_at	---	---	3.75	3.79	0	-0.03	-1.02	0.76	0.476
238982_at	DENR	density-regulated protein	4.55	4.42	0	0.13	1.10	0.30	0.319
239014_at	CCAR1	cell division cycle and apoptosis regulator 1	6.85	6.87	0	-0.02	-1.01	0.86	0.507
239042_at	TSR1	TSR1, 20S rRNA accumulation, homolog (S. cerevisiae)	5.29	5.16	0	0.13	1.10	0.26	0.299
239131_at	---	---	5.12	5.03	0	0.09	1.07	0.42	0.369
239351_at	---	---	3.19	3.19	0	0.00	1.00	0.99	0.540
239487_at	---	---	3.22	3.21	0	0.01	1.01	0.92	0.526
239504_at	---	---	6.06	5.96	0	0.10	1.07	0.29	0.315
239614_x_at	---	---	3.50	3.44	0	0.06	1.04	0.63	0.449
239651_at	ANAPC5	anaphase promoting complex subunit 5	5.14	5.05	0	0.09	1.07	0.52	0.409
239660_at	RALGAPA2	Ral GTPase activating protein, alpha subunit 2 (catalytic)	4.72	4.73	0	-0.02	-1.01	0.82	0.494
239674_at	---	---	3.04	3.12	0	-0.07	-1.05	0.21	0.269
239692_at	---	---	5.44	5.32	0	0.12	1.08	0.45	0.382
239742_at	TULP4	tubby like protein 4	7.13	7.22	0	-0.09	-1.06	0.21	0.269
239751_at	---	---	2.97	2.92	0	0.05	1.03	0.35	0.342
239771_at	CAND1	cullin-associated and neddylation-dissociated 1	5.38	5.32	0	0.06	1.04	0.57	0.428
239778_x_at	---	---	2.98	2.90	0	0.08	1.06	0.22	0.276
239809_at	---	---	3.80	3.78	0	0.02	1.01	0.89	0.517
239819_at	---	---	4.41	4.45	0	-0.05	-1.03	0.57	0.428
239893_at	---	---	4.91	4.84	0	0.07	1.05	0.61	0.441
239959_x_at	---	---	5.73	5.76	0	-0.03	-1.02	0.73	0.468
239978_at	---	---	7.53	7.57	0	-0.04	-1.03	0.73	0.468
240019_at	---	---	5.70	5.64	0	0.06	1.04	0.64	0.450
240062_at	FAM3C	family with sequence similarity 3, member C	2.46	2.49	0	-0.03	-1.02	0.57	0.428
240078_at	SFSWAP	splicing factor, suppressor of white-apricot family	4.13	4.13	0	0.00	-1.00	0.98	0.538
240141_at	---	---	5.44	5.39	0	0.05	1.04	0.60	0.441
240165_at	---	---	7.29	7.16	0	0.12	1.09	0.39	0.357
240248_at	---	---	4.14	4.10	0	0.04	1.03	0.64	0.449
240265_at	TRAF3IP3	TRAF3 interacting protein 3	5.75	5.63	0	0.11	1.08	0.40	0.363
240271_at	---	---	6.27	6.20	0	0.06	1.05	0.53	0.416
240401_at	---	---	2.51	2.47	0	0.04	1.03	0.52	0.412
240498_at	---	---	5.62	5.72	0	-0.10	-1.07	0.24	0.289
240602_at	HBS1L	HBS1-like translational GTPase	4.18	4.21	0	-0.03	-1.02	0.68	0.455
240613_at	JAK1	Janus kinase 1	3.92	3.89	0	0.04	1.02	0.68	0.456
240636_at	---	---	3.45	3.36	0	0.09	1.06	0.24	0.292
240652_at	---	---	5.07	5.08	0	-0.01	-1.01	0.93	0.526
240773_at	---	---	4.27	4.42	0	-0.15	-1.11	0.27	0.302
240789_at	---	---	3.84	3.77	0	0.07	1.05	0.55	0.422
240941_at	---	---	5.26	5.34	0	-0.08	-1.06	0.33	0.334
240951_at	RORA	RAR-related orphan receptor A	2.79	2.79	0	0.01	1.00	0.92	0.526
240957_at	---	---	3.41	3.50	0	-0.09	-1.06	0.16	0.236
240990_at	RAB5A	RAB5A, member RAS oncogene family	5.25	5.31	0	-0.06	-1.04	0.64	0.450
241091_at	AGO3	argonaute RISC catalytic component 3	5.36	5.32	0	0.04	1.03	0.63	0.448
241155_at	---	---	6.83	6.79	0	0.04	1.03	0.68	0.456
241263_at	RAB21	RAB21, member RAS oncogene family	3.07	3.01	0	0.06	1.04	0.38	0.354
241275_at	CAPZA1	capping protein (actin filament) muscle Z-line, alpha 1	4.28	4.20	0	0.08	1.06	0.40	0.363
241345_at	R186 /// ZRAN	microRNA 186 /// zinc finger, RAN-binding domain containing 2	4.74	4.71	0	0.03	1.02	0.55	0.422
241347_at	RNF213	ring finger protein 213	8.93	8.92	0	0.01	1.01	0.90	0.519
241403_at	CLK4	CDC like kinase 4	5.65	5.68	0	-0.03	-1.02	0.65	0.450
241462_at	.OC10537473	uncharacterized LOC105374738	5.29	5.18	0	0.11	1.08	0.37	0.349
241508_at	---	---	6.91	6.92	0	0.00	-1.00	0.95	0.533
241532_at	---	---	2.27	2.26	0	0.01	1.01	0.83	0.499
241620_at	SMCHD1	structural maintenance of chromosomes flexible hinge domain containing 1	4.61	4.64	0	-0.03	-1.02	0.79	0.484
241683_at	HECTD1	HECT domain containing E3 ubiquitin protein ligase 1	3.90	3.92	0	-0.02	-1.01	0.77	0.479
241685_x_at	PURA	purine-rich element binding protein A	3.82	3.81	0	0.00	1.00	0.97	0.536
241716_at	HSPD1	heat shock 60kDa protein 1 (chaperonin)	3.00	2.74	0	0.26	1.19	0.16	0.236
241732_at	---	---	3.75	3.66	0	0.08	1.06	0.35	0.342
241755_at	UQCRC2	ubiquinol-cytochrome c reductase core protein II	3.51	3.38	0	0.13	1.09	0.26	0.296
241788_x_at	---	---	2.82	2.84	0	-0.02	-1.01	0.76	0.477
241859_at	PLCL1	phospholipase C-like 1	2.98	3.06	0	-0.08	-1.06	0.27	0.301
241866_at	SLC16A7	solute carrier family 16 (monocarboxylate transporter), member 7	3.54	3.62	0	-0.08	-1.06	0.20	0.265
241879_at	LPP	LIM domain containing preferred translocation partner in lipoma	3.97	3.85	0	0.12	1.09	0.25	0.293
241885_at	---	---	5.29	5.11	0	0.18	1.13	0.41	0.366
241889_at	NFKBID	nuclear factor of kappa light polypeptide gene enhancer in B-cells inhibitor, delta	6.87	6.77	0	0.10	1.07	0.33	0.332
241947_at	PCED1B-AS1	PCED1B antisense RNA 1	5.68	5.62	0	0.05	1.04	0.52	0.412
241954_at	FDFT1	farnesyl-diphosphate farnesyltransferase 1	5.91	5.75	0	0.17	1.12	0.14	0.219
242037_at	ASPH	aspartate beta-hydroxylase	5.10	5.17	0	-0.06	-1.05	0.43	0.374
242099_at	---	---	3.60	3.62	0	-0.02	-1.01	0.77	0.478
242163_at	THRAP3	thyroid hormone receptor associated protein 3	4.23	4.14	0	0.09	1.06	0.51	0.409
242182_x_at	ITCH	---	2.71	2.65	0	0.06	1.05	0.13	0.209
242234_at	XAF1	XIAP associated factor 1	6.45	6.27	0	0.18	1.13	0.34	0.338
242248_at	PHKB	phosphorylase kinase, beta	3.26	3.27	0	-0.01	-1.00	0.94	0.529
242261_at	IREB2	iron responsive element binding protein 2	5.45	5.26	0	0.20	1.15	0.20	0.263
242289_at	MRPL42	mitochondrial ribosomal protein L42	3.15	3.09	0	0.07	1.05	0.37	0.349
242306_at	---	---	4.10	4.15	0	-0.05	-1.03	0.56	0.424
242408_at	STYX	serine/threonine/tyrosine interacting protein	4.08	4.00	0	0.08	1.05	0.31	0.323
242422_at	G3BP1	GTPase activating protein (SH3 domain) binding protein 1	5.49	5.54	0	-0.04	-1.03	0.78	0.482
242427_at	WAC	WW domain containing adaptor with coiled-coil	6.15	6.18	0	-0.03	-1.02	0.84	0.499
242432_at	9774 /// LOC1	uncharacterized LOC101929774 /// uncharacterized LOC105376844	2.81	2.90	0	-0.09	-1.07	0.12	0.202
242433_at	ZBTB11	zinc finger and BTB domain containing 11	5.11	5.12	0	-0.01	-1.01	0.92	0.526
242440_at	---	---	5.19	4.95	0	0.24	1.18	0.24	0.292
242496_at	ART4	ADP-ribosyltransferase 4 (Dombrock blood group)	3.16	3.04	0	0.12	1.09	0.28	0.304
242509_at	---	---	4.10	4.19	0	-0.09	-1.06	0.47	0.390
242540_at	DNHD1	dynein heavy chain domain 1	3.02	3.06	0	-0.04	-1.03	0.60	0.441
242563_at	---	---	5.85	5.76	0	0.10	1.07	0.34	0.338
242576_x_at	---	---	5.01	4.92	0	0.09	1.07	0.45	0.381
242588_at	---	---	2.86	2.92	0	-0.06	-1.04	0.38	0.355
242672_at	---	---	3.94	3.93	0	0.01	1.01	0.94	0.529
242732_at	---	---	4.64	4.78	0	-0.14	-1.10	0.27	0.302
242740_at	---	---	3.65	3.60	0	0.05	1.04	0.40	0.362
242774_at	SYNE2	spectrin repeat containing, nuclear envelope 2	7.08	6.97	0	0.10	1.07	0.24	0.290
242859_at	---	---	5.81	5.64	0	0.16	1.12	0.27	0.303
242871_at	PAQR5	progestin and adipoQ receptor family member V	2.72	2.72	0	0.00	1.00	0.97	0.536
242879_x_at	AKT3	v-akt murine thymoma viral oncogene homolog 3	4.79	4.62	0	0.18	1.13	0.16	0.233
242924_at	---	---	3.71	3.72	0	-0.01	-1.01	0.91	0.525
242929_at	---	---	3.67	3.58	0	0.09	1.06	0.42	0.368
242970_at	DIP2B	disco-interacting protein 2 homolog B	4.60	4.58	0	0.03	1.02	0.69	0.457
242975_s_at	---	---	5.61	5.51	0	0.11	1.08	0.28	0.308
242989_at	STRN	striatin, calmodulin binding protein	4.99	4.93	0	0.06	1.04	0.38	0.351
243023_at	---	---	3.87	3.84	0	0.03	1.02	0.68	0.455
243031_at	---	---	3.55	3.51	0	0.04	1.03	0.67	0.454
243064_at	---	---	3.95	3.97	0	-0.02	-1.02	0.73	0.468
243067_at	---	---	4.02	3.97	0	0.06	1.04	0.39	0.356
243107_at	CCR7	chemokine (C-C motif) receptor 7	5.59	5.53	0	0.06	1.04	0.60	0.441
243182_at	---	---	3.21	3.27	0	-0.05	-1.04	0.59	0.438
243229_at	---	---	3.29	3.35	0	-0.06	-1.04	0.33	0.332
243264_s_at	44 /// C8orf44	chromosome 8 open reading frame 44 /// C8orf44-SGK3 readthrough	4.09	4.00	0	0.09	1.07	0.26	0.299
243293_at	---	---	4.59	4.68	0	-0.09	-1.06	0.15	0.231
243299_at	---	---	5.64	5.68	0	-0.03	-1.02	0.65	0.450
243304_at	AGO2	argonaute RISC catalytic component 2	4.58	4.58	0	0.00	1.00	0.98	0.540
243319_at	---	---	3.18	3.22	0	-0.04	-1.03	0.63	0.449
243332_at	.OC10537961	uncharacterized LOC105379610	5.57	5.50	0	0.08	1.05	0.33	0.333
243360_at	---	---	2.73	2.73	0	0.00	1.00	0.99	0.542
243375_at	---	---	3.80	3.84	0	-0.04	-1.03	0.67	0.454
243473_at	---	---	2.50	2.55	0	-0.05	-1.03	0.34	0.338
243537_at	---	---	4.19	4.05	0	0.14	1.10	0.18	0.252

Mean42 LRS	Mean 47 ROT1D	Mean 30 HRS	Mean 44 uHC	LR024	HR014	HR026	LR001	LR022	LR013	HR015	LR020	LR018	HR027	HR005	LR028	LR016	LR012	HR019	HR017	HR021	LR030	HR010	LR006	LR008	LR011	LR004	LR023	LR002

Probeset ID	Gene Symbol	Gene Title	Mean log2 intensity for 21 POST	Mean log2 intensity for 21 PRE	422 FDR <0.2 1=YES 0=NO	Log2 Ratio 21POST vs21PRE	Fold-Change 21POST vs21PRE	p value	FDR
243552_at	MBTD1	mbt domain containing 1	3.99	3.90	0	0.09	1.07	0.12	0.204
243570_at	SPCS2	signal peptidase complex subunit 2	2.70	2.71	0	-0.01	-1.01	0.85	0.504
243631_at	---	---	5.82	5.87	0	-0.05	-1.03	0.39	0.357
243648_at	ZBED6	zinc finger, BED-type containing 6	6.35	6.46	0	-0.12	-1.08	0.26	0.295
243673_at	---	---	3.43	3.39	0	0.04	1.03	0.69	0.457
243739_at	---	---	4.84	4.83	0	0.02	1.01	0.84	0.500
243751_at	CHD2	chromodomain helicase DNA binding protein 2	7.08	6.95	0	0.12	1.09	0.34	0.338
243767_at	---	---	3.80	3.74	0	0.06	1.04	0.49	0.402
243835_at	ZDHHC21	zinc finger, DHHC-type containing 21	5.00	5.00	0	0.00	-1.00	0.97	0.535
243940_at	TSHZ2	teashirt zinc finger homeobox 2	2.70	2.70	0	0.00	-1.00	0.96	0.533
243959_at	---	---	3.11	3.15	0	-0.04	-1.03	0.54	0.419
243996_at	NPR2	natriuretic peptide receptor 2	2.94	2.84	0	0.10	1.07	0.24	0.292
244035_at	---	---	6.27	6.23	0	0.04	1.03	0.52	0.412
244055_at	---	---	4.98	4.90	0	0.09	1.06	0.19	0.258
244125_at	---	---	5.44	5.38	0	0.05	1.04	0.52	0.409
244153_at	ZNF207	zinc finger protein 207	5.10	5.15	0	-0.05	-1.03	0.51	0.409
244171_at	MKLN1	muskelin 1, intracellular mediator containing kelch motifs	2.91	2.93	0	-0.02	-1.01	0.86	0.506
244219_at	WTAP	Wilms tumor 1 associated protein	5.02	5.01	0	0.01	1.01	0.93	0.526
244263_at	---	---	3.29	3.36	0	-0.07	-1.05	0.39	0.356
244396_at	G3BP1	GTPase activating protein (SH3 domain) binding protein 1	3.57	3.69	0	-0.12	-1.09	0.11	0.201
244443_at	LINC01578	long intergenic non-protein coding RNA 1578(LINC01578)	4.33	4.34	0	-0.01	-1.00	0.94	0.528
244508_at	SEPT7	septin 7	5.30	5.21	0	0.09	1.07	0.15	0.229
244546_at	CYCS	cytochrome c, somatic	4.37	4.25	0	0.13	1.09	0.22	0.278
244568_at	---	---	4.63	4.64	0	-0.01	-1.01	0.92	0.526
244579_at	---	---	7.11	7.19	0	-0.08	-1.06	0.37	0.349
244669_at	SNORD50A ///	small nucleolar RNA host gene 5 /// small nucleolar RNA, C/D box 50A /// small nucleolar RNA, C/D box 50B	8.41	8.42	0	0.00	-1.00	0.95	0.533
244674_at	---	---	4.62	4.78	0	-0.15	-1.11	0.23	0.286
244737_at	---	---	3.95	3.93	0	0.03	1.02	0.68	0.455
244763_at	MTRF1	mitochondrial translational release factor 1	2.69	2.67	0	0.03	1.02	0.69	0.457
244780_at	SGPP2	sphingosine-1-phosphate phosphatase 2	4.36	4.21	0	0.15	1.11	0.32	0.328
244791_at	---	---	4.82	4.77	0	0.05	1.04	0.64	0.449
244794_at	LOC10537487	uncharacterized LOC105374872	5.13	5.07	0	0.06	1.04	0.55	0.423
244804_at	SQSTM1	sequestosome 1	9.11	9.07	0	0.05	1.03	0.65	0.450
244840_x_at	DOCK4	dedicator of cytokinesis 4	5.76	5.85	0	-0.09	-1.06	0.45	0.380
244846_at	---	---	4.70	4.83	0	-0.13	-1.10	0.33	0.333
244847_at	---	---	3.71	3.65	0	0.06	1.04	0.37	0.349
244872_at	RBBP4	retinoblastoma binding protein 4	4.05	3.92	0	0.13	1.10	0.16	0.236
33148_at	ZFR	zinc finger RNA binding protein	5.86	5.89	0	-0.02	-1.02	0.70	0.458
37145_at	GNLY	granulysin	10.72	10.67	0	0.05	1.04	0.49	0.400
39402_at	IL1B	interleukin 1 beta	12.38	12.45	0	-0.07	-1.05	0.35	0.342
60794_f_at	ZNF814	zinc finger protein 814	2.94	2.90	0	0.04	1.03	0.66	0.450
60815_at	POLR2J4	polymerase (RNA) II (DNA directed) polypeptide J4, pseudogene	5.24	5.13	0	0.10	1.07	0.52	0.412

Mean42 LRS	Mean 47 ROT1D	Mean 30 HRS	Mean 44 uHC	LR024	HR014	HR026	LR001	LR022	LR013	HR015	LR020	LR018	HR027	HR005	LR028	LR016	LR012	HR019	HR017	HR021	LR030	HR010	LR006	LR008	LR011	LR004	LR023	LR002
